# Unveiling genomic regions that underlie differences between Afec-Assaf sheep and its parental Awassi breed

**DOI:** 10.1186/s12711-017-0296-3

**Published:** 2017-02-10

**Authors:** Eyal Seroussi, Alexander Rosov, Andrey Shirak, Alon Lam, Elisha Gootwine

**Affiliations:** Institute of Animal Science, ARO, The Volcani Center, PO Box 15159, 7528809 Rishon LeZion, Israel

## Abstract

**Background:**

Sheep production in Israel has improved by crossing the fat-tailed local Awassi breed with the East Friesian and later, with the Booroola Merino breed, which led to the formation of the highly prolific Afec-Assaf strain. This strain differs from its parental Awassi breed in morphological traits such as tail and horn size, coat pigmentation and wool characteristics, as well as in production, reproductive and health traits. To identify major genes associated with the formation of the Afec-Assaf strain, we genotyped 41 Awassi and 141 Afec-Assaf sheep using the Illumina Ovine SNP50 BeadChip array, and analyzed the results with PLINK and EMMAX software. The detected variable genomic regions that differed between Awassi and Afec-Assaf sheep (variable genomic regions; VGR) were compared to selection signatures that were reported in 48 published genome-wide association studies in sheep. Because the Afec-Assaf strain, but not the Awassi breed, carries the Booroola mutation, association analysis of *BMPR1B* used as the test gene was performed to evaluate the ability of this study to identify a VGR that includes such a major gene.

**Results:**

Of the 20 detected VGR, 12 were novel to this study. A ~7-Mb VGR was identified on *Ovies aries* chromosome OAR6 where the Booroola mutation is located. Similar to other studies, the most significant VGR was detected on OAR10, in a region that contains candidate genes affecting horn type (*RXFP2*), climate adaptation (*ALOX5AP*), fiber diameter (*KATNAl1*), coat pigmentation (*FRY*) and genes associated with fat distribution. The VGR on OAR2 included *BNC2*, which is also involved in controlling coat pigmentation in sheep. Six other VGR contained genes that were shown to be involved in coat pigmentation by analyzing their mammalian orthologues. Genes associated with fat distribution in humans, including *GRB14* and *COBLL1*, were located in additional VGR. Sequencing DNA from Awassi and Afec-Assaf individuals revealed non-synonymous mutations in some of these candidate genes.

**Conclusions:**

Our results highlight VGR that differentiate the Awassi breed from the Afec-Assaf strain, some of which may include genes that confer an advantage to Afec-Assaf and Assaf over Awassi sheep with respect to intensive sheep production under Mediterranean conditions.

**Electronic supplementary material:**

The online version of this article (doi:10.1186/s12711-017-0296-3) contains supplementary material, which is available to authorized users.

## Background

In Israel, within-breed selection, crossbreeding and gene introgression have contributed to the transition of the sheep industry from traditional extensive production using the native fat-tailed local Awassi breed to highly intensive production with the Assaf and Afec-Assaf sheep [1]. Selection within the local Awassi sheep that began in the 1930s led to the formation of the improved Awassi dairy strain [2]. The Assaf dairy breed was formed about 30 years later by crossing the improved Awassi with the European East Friesian (EF) breed [3]. In 1986, breeding of the highly prolific Afec-Assaf strain was initiated by introgressing the Booroola mutation (*B*) at the *BMPR1B* gene into the Assaf breed by crossing Assaf ewes with Booroola Merino rams [4–6]. The high prolificacy of the Afec-Assaf strain is due to the presence of the *B* allele, which is inherited in an almost completely dominant mode [7], since *BB* and *B*+ ewes have similar prolificacy. However, since the perinatal lamb survival rate is higher in *B*+ than in *BB* ewes, *B*+ is the recommended genotype for females in commercial flocks [7]. The *B* allele segregates in the Afec-Assaf population, thus genotyping lambs for the Booroola mutation and selecting only the *B*+ ewe lambs as replacements is a common practice in Afec-Assaf flocks to maintain high prolificacy in the long term. Afec-Assaf sheep differ widely from the Awassi sheep in a number of phenotypic characteristics, such as the presence of horns, tail shape, coat color pigmentation and wool quality, as well as seasonality, prolificacy, and milk production [8, 9] (Table 1).

**Table 1:** Phenotypic, production and reproductive traits of local Awassi, improved Awassi, Assaf and Afec-Assaf sheep

Traits	Local Awassi	Improved Awassi	Assaf	Afec-Assaf
Body size	Small^a^	Large^a^	Large^a^	Large^a^
Tail shape	Fat tail	Fat tail	Partial fat tail	Partial fat tail
Horn shape	Most rams and ewes are horned or carry scurs		Most males and females are polled, but some carry short knobs or scurs	
Wool type	Coarse	Coarse	Medium-coarse	Medium-coarse
Coat pigmentation	Most sheep have brown face and white fleece		Most sheep have white face and white fleece	
Seasonality	Seasonal	Seasonal	Moderately seasonal	Moderately seasonal
Adaptation to Middle-Eastern climate	High	High	Intermediate	Intermediate
Milk production	Low	High	High	High
Prolificacy (lambs born/ewe lambing)	1.2	1.3	1.6	2.5

^a^Small: ram body weight ~70 kg; large: ram body weight ~110 kg

In sheep, genome-wide association studies (GWAS) have contributed to better understanding of their domestication [10, 11], as well as to the identification of specific genomic regions that are associated with differences in various traits between breeds (see Additional file 1: Table S1). GWAS have made it possible to identify candidate genes that underlie various phenotypic differences, for instance in Texel sheep *RXFP2* for horn type [12] and disease-resistance genes such as *PITX3* and *DMP1* which are involved in microphthalmia [13] and in Corriedale sheep for rickets [14].

Cryptic relatedness, which occurs when there is unknown kinship within the sample, and population stratification due to random genetic drift in the sample’s subpopulations are two major confounding effects that cause spurious associations in GWAS analyses [15]. Hence, incorporating a known genetic marker that is located within a major gene that segregates within the tested population into the GWAS analysis may assist in verifying the power of the study for detecting selection signatures. This has been the case in several sheep GWAS, in which the *MC1R* gene that affects coat pigmentation [16] was used as the ‘test’ gene, and polymorphism at the *GDF8* gene associated with muscle hypertrophy [17] was used to establish criteria to detect selection signatures.

By using the ovine single nucleotide polymorphism (SNP) 50 BeadChip array (Illumina Inc., San Diego, CA) and *BMPR1B* as a test gene, the aims of our study were to: (1) compare the Awassi and Afec-Assaf genomes, searching for variable genomic regions (VGR) that differ between the two breeds; and (2) link these VGR to genes and selection signatures that were previously described in sheep GWAS.

## Methods

### Ethics statement

Experimental protocols were approved (Approval No. IL 415-12) by the Volcani Center Institutional Animal Care and Use Committee.

### Animals

Local Awassi sheep are raised by Bedouin growers in small unconnected flocks that are maintained under extensive traditional management with no records. The Awassi cohort consisted of 24 local Awassi rams that included all of the rams from five flocks, and 17 improved Awassi ewes from the Ein-Harod flock. Since the degree of relationship between animals from the same flock is unknown, the effective number of local Awassi individuals might be smaller than sampled. However, this was not the case, since a genomic relationship analysis (see Additional file 2: Table S2) showed no strong kinship between individuals within or among flocks. The Afec-Assaf cohort consisted of 22 males and 119 females from the experimental flock of the Volcani Center at Bet Dagan and two commercial farms. Genotyping indicated that 35, 95 and 11 animals of the Afec-Assaf cohort were homozygous *BB*, heterozygous *B*+ or non-carrier ++ for the Booroola mutation, respectively. As expected [7], the average prolificacy of the *BB* and *B*+ females (based on 3 to 9 parity records per animal) was high, i.e. 3.1 and 3.0 lambs born/lambing, respectively. No lambing records were available for non-carrier ++ females.

### Genotyping using the ovine 50 K beadchip

DNA was extracted from blood samples using the DNeasy Blood & Tissue Kit (Qiagen, Valencia, CA) according to the manufacturer’s instructions. DNA samples were genotyped by the Ovine SNP50 BeadChip assay using standard procedures (http://www.illumina.com). All the Awassi and some of the Afec-Assaf samples were genotyped as part of the International Sheep Genomic Consortium initiative (http://www.sheephapmap.org). Other Afec-Assaf samples were genotyped at the Centre for Reproduction and Genomics (CRG), Invermay, New Zealand.

Of the 59,454 SNPs on the ovine SNP50 BeadChip, 46,563 SNPs that were randomly distributed across the genome passed the quality-control filter criteria (call rate higher than 99%, genotyping frequency higher than 95%, minor allele frequency lower than 0.05 and Hardy–Weinberg equilibrium *P* > 0.001), and were used for further analyses.

### Genotyping at the *RXFP2* gene

Phenotypic variation in horn appearance includes the presence of normal horns (in females they are smaller than in males), deformed horns (scurs), short knobs at the site of horn growth, and a polled-non-horned growth phenotype, which may include a concave depression in the skull bone at the horn site [18]. DNA from Awassi and Afec-Assaf sheep with different horn phenotypes was extracted from blood samples or buccal swabs using standard DNA-extraction protocols. Primers, PCR conditions and separation of PCR products on an agarose gel to genotype animals that carry a 1833-bp genomic insertion located in the 3′-UTR of *RXFP2*, were performed according to Wiedemar and Drogemuller [19]. Association between the genotype at the *RXFP2* gene and horn appearance was tested by Chi square analysis.

### DNA sequencing

To obtain preliminary information on variation of the coding regions of genes that are included in VGR, DNA of one Afec-Assaf individual and one Awassi individual, selected randomly, was sequenced using the Illumina 100-bp paired-end technology (HiSeq 2000, Illumina) with one or two lanes, respectively. The sequences were deposited in the European Nucleotide Archive (ENA) under accession number PRJEB12018. Achieved coverage was 11- and 26-fold for the Afec-Assaf and Awassi individuals, respectively.

### Identification of candidate polymorphisms

Exon sequences of the candidate genes were downloaded from NCBI (https://www.ncbi.nlm.nih.gov) and genomic reads were mapped to these templates using GAP5 software [20]. BWA options for this mapping were set to bam bwasw -t 8 -T 30 [21]. Genetic markers were identified and called by comparing the consensus contig sequences of the Awassi and Assaf individuals with the GAP5 contig comparator and the heterozygosity search option (value = 10).

### Scoring amino acid substitutions

Amino acid substitutions were evaluated using PROVEAN analysis v1.1 [22] which scores the effect of a variation in protein sequence on the function of that protein.

### Statistical analysis

Various methods have been applied to verify selection signatures in cattle [23] as well as in sheep. To minimize the risk of detecting spurious associations in our GWAS analysis, we applied two tools: PLINK, which is suitable for calculating either *F*_st_ values or probability values for genetic association test and EMMAX that calculates these probability values using a relationship matrix that accounts for relatedness within the sample. Association between SNPs and breed (Awassi or Afec-Assaf) were evaluated using PLINK v1.90b3.38 64-bit [24]. Probability (Fisher’s exact test of significance) and *F*_st_ values for differences in allele frequency were estimated with the following options on the command line: –sheep –hwe 0.001 –geno 0.1 –maf 0.05 –fst –assoc fisher. *F*_st_ estimates were obtained only for the autosomes and were computed using the method introduced by Weir and Cockerham. The Efficient Mixed-Model Association eXpedited (EMMAX) program [25] was used to identify genomic regions on autosomes and on the X chromosome. For the EMMAX analysis, the following options were used on the command lines: emmax-kin -v -h -s -d 10; emmax -v -d 10, for the creation of the identity by state (IBS) relationship matrix and for the association test, respectively.

Genomic inflation factors were equal to 9.35 and 1.34 for the unadjusted PLINK Fisher and EMMAX analyses, respectively. Using each of the programs, SNPs were ranked according to their association with one of the breeds by chance. The top ~0.1% (n = 50) significant SNPs and their adjacent neighboring SNPs, which ranked in the top 0.5% and were found within 1.5 Mb upstream or downstream of the top marker, were used to define VGR. Neighboring genomic regions were combined into one region if they overlapped. The Bonferroni-corrected significance threshold of 1.07 × 10^−6^ (0.05/46,563) was used to correct for multiple comparisons and the threshold for significance was set at *P* < 0.05. The position of regions was determined according to version 3.1 of the sheep genome assembly.

### Bioinformatics

Candidate regions were examined using the sheep genome browsers Build v3.1 [26–28]. We used the following databases: NCBI [29], GeneCards^®^ [30], and SheepQTL [31], as well as literature searches, to obtain gene annotations and functions.

## Results and discussion

**Fig. 1:**
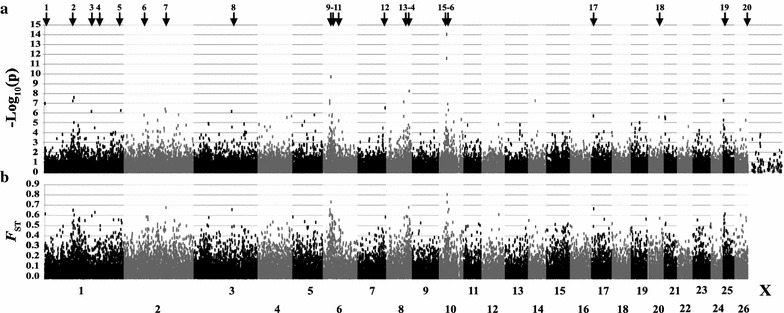
Manhattan plot of GWAS comparing Awassi and Afec-Assaf sheep

PLINK analysis showed a correlation of 0.98 between the values of *F*_st_ and the minus logarithm of the probability according to Fisher’s exact test (data not shown). Thus, since both parameters yielded similar estimates for allele frequency differences, we chose to consider only the *F*_st_ values in the further analyses. PLINK and EMMAX-based GWAS revealed 36 and 32 VGR, respectively (see Additional file 3: Table S3). *F*_st_ values for the most significant SNPs in each region in the PLINK analysis ranged from 0.46 to 0.80, and the corresponding *P* values in the EMMAX analysis ranged from <10^−5^ to <10^−15^. The 20 VGR that were detected by both PLINK and EMMAX analyses were further analyzed (Table 2 and Fig. 1).

**Table 2:** Variable genomic regions detected in Awassi and Afec-Assaf sheep according to both PLINK and EMMAX analyses

Variable genomic region number	OAR number	Region position (Mb)	PLINK - Peak SNP position (Mb)	EMMAX -Peak SNP position (Mb)	PLINK *F*_st_	EMMAX P value
1	1	2.72	2,721,556		0.61	1.0e−07
2		99.83–101.28	99,832,319		0.65	5.4e−08
3		103.40–103.45	103,452,513		0.60	2.5e−08
4		164.60	164,604,667		0.59	6.7e−07
5		266.24	266,241,278		0.55	5.3e−07
6	2	83.11–84.53	83,214,641	84,054,194	0.59	1.0e−05
7		145.65–148.41	148,406,594	145,648,041	0.68	3.9e−07
8	3	134.23–134.49	134,234,489		0.66	6.7e−07
9	6	20.99–24.00	22,323,737		0.66	5.1e−08
10		24.97–32.04	26,058,630		0.73	1.8e−10
11		54.26–55.67	54,261,462		0.55	5.8e−06
12	7	96.99	96,995,379		0.58	2.9e−07
13	8	62.62–62.83	62,735,336		0.57	6.8e−08
14		78.36–80.60	78,364,891	80,605,889	0.67	5.2e−09
15	10	27.65–31.64	30,700,853		0.80	8.4e−15
16		33.75–36.24	36,238,012	33,752,924	0.66	1.2e−07
17	17	8.13–8.74	8,739,357		0.66	1.9e−06
18	20	37.51	37,515,740		0.57	2.5e−06
19	25	6.65–8.66	7,392,689		0.59	4.7e−08
20	26	36.58	36,580,518		0.58	5.4e−06

### Variable genomic regions on OAR6 (between 24.97 and 32.04 Mb)

As expected, a VGR (#10) that spanned the *BMPR1B* gene (between positions 29.36 and 29.45 Mb) was identified on OAR6 (Table 2), with the most significant SNP, s24937.1, being located about 3.0 Mb upstream of the gene. None of the four SNPs of the Illumina Ovine SNP50 BeadChip that are located within non-coding regions of *BMPR1B* namely: OAR6_33210385.1, OAR6_33229928.1, OAR6_33291928.1 or s21306, reached significance (*P* > 0.05). DNA sequencing showed that the Afec-Assaf individual was heterozygous for the Booroola mutation, while the Awassi individual did not carry the mutation.

A similar level of precision in the detection of a point mutation was obtained in a GWAS applied in Texel sheep to map the causative mutation for microphthalmia, which revealed a region of about 2.6 Mb [13]. In the case of the autosomal recessive lethal disorder brachygnathia, cardiomegaly and renal hypoplasia syndrome in polled Merino sheep, SNPs that are associated with this syndrome are spread across 1.1 Mb [32]. However, the precision that was achieved in a GWAS for detecting the causative mutation for dominant pigmentation in the *MC1R* gene was higher [16], with only one significant SNP that was 25 kb away from *MC1R*, being identified for the Manchega sheep population, in which this trait segregates.

The VGR on OAR6 that includes the *BMPR1B* gene also emerged in a GWAS that searched for genomic differences between dairy and non-dairy sheep breeds [17], and in another study that focused on quantitative trait loci (QTL) for carcass traits [33]. Since the Booroola mutation was introgressed into the Assaf breed from the small-size non-dairy Booroola Merino breed, the genomic difference that was found between the Awassi and Assaf breeds at around 29 Mb on OAR6 might also be related to the difference in allelic frequency of genes that control these traits. One relevant gene was *ABCG2*, which has a major effect on milk production, and is mapped to OAR6 at 36.5 Mb, about 3.0 Mb downstream to the 3′ end of that VGR [17].

### Variable genomic regions on OAR10 that span 30 Mb

A major VGR (#15) was found on OAR10 (between positions 27.65 and 31.64 Mb, Table 2). Significant selection signatures in this chromosomal region have been detected in several GWAS in which sheep breeds were compared for horn phenotype, climate adaptation, fiber diameter, coat pigmentation or fat distribution [10–12, 18, 34–39].

### Horn phenotype

Awassi rams are mostly horned, as well as some of the ewes, whereas most Assaf sheep are polled, similar to the situation observed in the EF parental breed. Indeed, the *RXFP2* gene (on OAR10 between 29.45 and 29.50 Mb), which is involved in horn inheritance [12], was identified in the middle of this OAR10 VGR (Table 2). To further corroborate this, we genotyped 61 horned and non-horned Awassi and Afec-Assaf sheep (see Additional file 4: Figure S1), to identify those that carried a 1833-bp genomic insertion located in the 3′-UTR of *RXFP2.* It has been shown that this insertion is linked to horn inheritance in Swiss sheep breeds [19]. Our results (see Additional file 5: Table S4) show that in Awassi and Afec-Assaf sheep, there is also an association between the *RXFP2* polymorphism in the 3′-UTR and the hornness-polledness phenotype (*P* < 0.0001), with absence of the insertion being associated with the horned phenotype. However, the range of phenotypes observed in the group of heterozygous animals indicated that other genetic modifications may also underlie the horned phenotype. A recent study of 38 sheep breeds [40] reached similar conclusions i.e. that the 1.78-kb insertion in the 3′-UTR of *RXFP2* cannot be considered as the only cause of polledness in sheep.

### Climate adaptation

The EF and Awassi breeds—the parental breeds of the Afec-Assaf—differ in their adaptability to the hot and humid Mediterranean climate. Susceptibility to pneumonia in the summer is a major death threat in Israel for pure EF but not Awassi lambs [41]. A GWAS that investigated environmental adaptive selection in sheep [36] suggested *ALOX5AP* (on OAR10 between 30.36 and 30.38 Mb) as a candidate gene for climate adaptation. Located in the distal part of VGR #15 on OAR10, this gene encodes a protein that is required for the synthesis of leukotrienes, which are biologically active lipid mediators involved in various types of inflammatory responses. In humans, polymorphism in *ALOX5AP* has been associated with lung function [42, 43].

### Fiber diameter

In a GWAS that included Chinese sheep breeds, differences in the VGR on OAR10 were associated with differences in the coefficient of variation of fiber diameter [37]. Indeed, the Awassi and Assaf breeds differ in their variation in fiber diameter, with the Awassi’s fibers being more uniform [44]. The *KATNAL1* gene (on OAR10 between 30.77 and 30.78 Mb) was the closest gene mapped to the most significant SNP (OAR10: 30,700,856).

### Coat pigmentation

As indicated in Table 1, the coat color of most Afec-Assaf sheep is white, while most Awassi sheep have reddish-brown pigmentation on their head, neck and legs. The mode of inheritance of coat pigmentation pattern in sheep is complex, as already shown for the piebald phenotype in Merino sheep [45]. Of the 11 genes that have been suggested as candidates that affect sheep coat pigmentation (see Additional file 6: Table S5), *FRY* (on OAR10 between 28.98 and 29.19 Mb) was located in the middle of VGR #15 on OAR10. *FRY* is a key candidate gene for the piebald phenotype in Merino sheep [45] and for coat color differences between Rambouillet (white coat color) and Suffolk (black head and legs) sheep [35], and is associated with the black spot phenotype in Valley-type Tibetan sheep [38]. Thus, polymorphism associated with the *FRY* gene may contribute to the differences in coat color pigmentation distribution between the Awassi and Afec-Assaf sheep.

### Sequence analysis

Comparing the coding sequences of the three candidate genes, namely: *ALOX5AP, KATNAL1* and *FRY*, of Awassi and Assaf individuals revealed two synonymous mutations, no sequence variation and three conservative amino-acid substitutions: p.V383I, p.V1207I and p.A1722S, respectively (see Additional file 7).

### Fat distribution

While the Awassi sheep have a relatively large fat tail, which usually hangs down to the hock, most Assaf and Afec-Assaf sheep carry a reduced-size fat tail which varies in size (see Additional file 8: Figure S2). Archeological evidence has documented the presence of fat-tailed sheep in the Fertile Crescent as early as 4000 years BC. In this region, sheep were the major source of animal fat, which was consumed as a delicacy and used for industrial purposes. Selection for fat tail was driven, among other reasons, by the convenience of harvesting the fat tissue from the carcasses. A study of body and carcass conformation of Awassi and Merino–Awassi crossbred sheep [46] demonstrated that the reduction in fat-tail size in Awassi crosses is associated with a change in body fat distribution: carrying a fat tail was associated with a decrease in the relative amount of body fat (omental, mesenteric, kidney, channel and scrotal fat), as well as a decrease in the relative amount of subcutaneous and intramuscular fat.

GWAS comparing fat- and thin-tailed sheep [38, 39, 47, 48] or studies on differential gene expression in such breeds [49–51] revealed several chromosomal regions that are associated with the presence of a fat tail (see Additional file 9: Table S6). Only two of these regions, i.e. regions numbered 14 and 15, both on OAR10, overlapped with VGR that distinguish Awassi from Afec-Assaf sheep. These two regions were identified in a study that compared Cyprus fat-tailed sheep, a breed related to the Awassi breed, and Laticauda sheep, to several Italian thin-tailed breeds [39].

Taken together, the VGR on OAR10 that spans a region between 27.65 and 31.64 Mb and detected in the current study, has been associated in various sheep GWAS with several phenotypic traits that differ between Awassi and Afec**-**Assaf sheep.

### Additional variable genomic regions associated with differences in coat pigmentation

Two of the coat pigmentation-related genes known in sheep: *BNC2* and *TYRP1* (see Additional file 6: Table S5) were localized, respectively, within or close to VGR #6 that spans a region between 83.11 and 84.53 Mb on OAR2 (Table 2). *BNC2* (on OAR2 between 84.29 and 84.69 Mb) was identified as a candidate gene for coat pigmentation in Italian breeds [11] with the most differentiated breed being the Comisana, which resembles the Awassi breed with a white body and brick-red face. The other breeds that contrasted with the Comisana were the Altamurana and Leccese white-coat breeds, and the Sardinian ancestral black sheep. Notably, the *TYRP1* gene (on OAR2 between 80.60 and 80.62 Mb), which is associated with skin and fiber pigmentation differences between Awassi and Merino sheep [52], and with pigmentation polymorphism in Soay sheep [53] and in Finnsheep [54], was mapped closely to VGR #6 (on OAR2 between 83.11 and 84.53). Comparing sequences of Awassi and Afec-Assaf *BNC2*- and *TYRP1*-coding regions (see Additional file 7) yielded one neutral substitution (p.W70R) and one conservative replacement, respectively.

To further search for possible candidate genes associated with coat pigmentation differences between Awassi and Afec-Assaf sheep, we compiled a list of 172 genes known to affect coat pigmentation in humans [55–57], cattle [58] and other mammals [59] (see Additional file 10: Table S7). Nine of these genes namely: *NTRK1*, *RPL24*, *SEMA4A*, *KRT1*, *KRT4*, *KRT75*, *BMPR1B*, *MAB21L2*, and *EDARADD* were located within, or close to one of the VGR that were identified on six ovine chromosomes.

Taken together, our results indicate that two of the coat pigmentation-related genes known in sheep: *FRY* and *BNC2,* and probably *TYRP1,* as well as some other genes have possible roles in the differences in coat pigmentation between Awassi and Afec-Assaf sheep.

### Additional variable genomic regions associated with differences in fat distribution

Fat distribution between subcutaneous and visceral adipose tissue in humans is genetically controlled [60]. After annotating 107 genes associated with fat distribution in humans [60–64] (see Additional file 11: Table S8), we found that the *DCST2* gene was annotated close to VGR #3 on OAR1 (between 103.40 and 103.45 Mb), and that two genes, *GRB14* and *COBLL1*, were both located close to VGR #7 on OAR2 (between 145.65 and 148.41 Mb). Only a few synonymous and conservative substitutions were identified in *DCST2* and *GRB14*, respectively, when the corresponding coding sequences of Awassi and Afec-Assaf sheep were compared (see Additional file 7). However, in the case of *COBLL1*, four non-synonymous substitutions were found, one of which, p.I448S (ref XP_012011424.1), was deleterious according to the PROVEN analysis [22]. If *COBLL1* is indeed involved in the control of differences in fat deposition in sheep, fat-tailed sheep could serve as a natural large animal model for studying the possible adverse metabolic and physiological outcomes of body fat distribution.

### Variable genomic regions not associated with a specific trait

Our analysis revealed additional VGR that were previously identified in other GWAS. However, these were not associated with a specific production, reproductive or health trait. VGR #13 on OAR8 (between 62.62 and 62.83 Mb) was detected in a GWAS that included multiple worldwide breeds [10], while VGR #16 on OAR10 (between 33.75 and 36.24 Mb) was detected in a GWAS that comprised five American breeds [35]. VGR #19 on OAR25 (between 6.65 and 8.66 Mb) was characterized as an “ancestral signature of selection” that differentiates European from Mediterranean breeds early in domestication [11]. It is worth noting that the EF and Awassi breeds, which are the parental breeds of the Afec-Assaf strain, belong to these two groups, respectively. This VGR on OAR25 was also identified in several studies in which different breeds were compared [10, 23, 38]. Finally, VGR #20 on OAR26 (36.58 Mb) was identified in a GWAS that analyzed the highly prolific Afec-Assaf ewes with different perinatal lamb viability rates [65].

Twelve of the 20 VGR identified in the current study, namely VGR #1, 2, 3, 4, 5, 7, 8, 9, 11, 12, 17 and 18 (Table 2) were not described in any of the 48 GWAS sheep studies listed in Table S1 (see Additional file 1: Table S1). These VGR, which may have arisen due to either selection or random genomic drift, may contain chromosomal regions that contribute to the genetic differences between the Awassi breed and its derived Afec-Assaf strain.

## Conclusions

Using the Illumina Ovine SNP50 BeadChip array, we revealed 20 VGR that are associated with differences in morphological, production and reproductive traits between the Afec-Assaf strain and the Awassi breed, a hardy Middle Eastern fat-tailed breed. Focusing on *BMPR1B* as a test gene allowed us to evaluate the ability of our study to identify VGR that harbor major genes. We compared our results to those of 48 GWAS in sheep, and GWAS in other domestic animals and in humans. Thus, we were able to highlight candidate genes that are involved in differences between Awassi and Afec-Assaf sheep regarding morphological traits, such as horn appearance, presence of a fat tail and coat pigmentation. In most cases, a comparison between the exon sequences of the candidate genes from Awassi and Afec-Assaf individuals revealed synonymous or neutral variations.

It is of interest that 60% of the VGR identified in the current study were not described in previous sheep GWAS that included a variety of sheep breeds. Bearing in mind that VGR may also arise from random drift, selection for genes that are included in these VGR may have further improved the Assaf breed and contributed to establish it as the major dairy breed in Israel and Spain, where it outperforms the local dairy breeds [66].

## Additional files

**Additional file 1: Table S1. **Genome-wide association studies in sheep reviewed in the current study [10–14, 17, 18, 32–39, 47, 48, 54, 65, 67–95].

**Additional file 2: Table S2. **Genomic relationship within and between local Awassi flocks.

**Additional file 3: Table S3. **Variable genomic regions (VGR) that differ between the Awassi and the Afec-Assaf according to PLINK and EMMAX analyses. SNPs were ranked according to their *P*-values, and the selected regions included the top 0.1% markers and their neighboring (1.5 Mb upstream and 1.5 Mb downstream) markers ranked in the top 1% of the markers. Two or more genomic regions were combined if they overlapped. Region position was determined using sheep genome assembly version 3.1. Regions identified by both analyses are marked in gray.

**Additional file 4: Figure S1. **Different horn phenotypes in Awassi (A to D) and Afec-Assaf (E to H) sheep. (A) Polled, female. (B) Knobs, female. (C) Horns, female. (D) Horns, male. (E) Polled, female. (F) Knobs, male. (G) Knobs, male. (H) Scurs, male.

**Additional file 5: Table S4. **Association between presence of a 1.8-kb insertion at the 3′-UTR of *RXFP2* and horn phenotype in Awassi and Afec-Assaf sheep. A 428-bp PCR product indicates the presence of the insertion [19].

**Additional file 6: Table S5. **Candidate genes for sheep coat colors and patterns [10, 11, 16, 34, 35, 38, 45, 52–54, 96, 97].

**Additional file 7. **Candidate gene sequences. This file contains sequences of candidate genes in a FASTA-like format. Each sequence header consists of the gene symbol followed by its reference GenBank accession number and the breed name. Polymorphic nucleotide positions are indicated in blue and red font colors.

**Additional file 8: Figure S2. **Variability in fat-tail phenotypes in Assaf sheep. Awassi-like fat tail (A). Fat tails with different amounts of fat deposition and different lengths (B to H).

**Additional file 9: Table S6. **Candidate genes and SNP markers associated with carrying fat tail in sheep [38, 39, 47, 49–51].

**Additional file 10: Table S7. **Genes known to affect coat pigmentation in humans, cattle and other mammals [55–58].

**Additional file 11: Table S8. **Human genes associated with fat distribution [61, 63, 64, 98–100].
