# Supplementary material for: Unveiling genomic regions that underlie differences between Afec-Assaf sheep and its parental Awassi breed
Source: Genet Sel Evol. 2017 Feb 10;49:19. doi: 10.1186/s12711-017-0296-3 (PMC5301402; doi:10.1186/s12711-017-0296-3)
Supplement: Supplementary file 1 — Additional file 1: Table S1. Genome-wide association studies in sheep reviewed in the current study [10–14, 17, 18, 32–39, 47, 48, 54, 65, 67–95]. [file 12711_2017_296_MOESM1_ESM.docx]

**Table S1.** Genome-wide association studies in sheep reviewed in the current study

| Breed comparison/Trait investigated | | Reference |
| --- | --- | --- |
| Breed comparison | |  |
| Suffolk – Rambouillet  Rambouillet, Columbia, Polypay, Targhee | | [35] |
| Adaptations to climate-mediated selective pressures | | [36] |
| Worldwide sheep populations | | [11] |
| Worldwide sheep populations | | [10] |
| Dairy vs. non-dairy breeds | | [17] |
| Northern Europeans breeds | | [67] |
| Chinese indigenous sheep breeds | | [38] |
| Italian indigenous sheep breeds | | [68] |
| Three domestic sheep breeds | | [48] |
| Three breeds | | [69] |
|  |  |  |
| Quantitative traits | |  |
| Milk yield | | [70] |
| Milk trait | | [71] |
| Mastitis susceptibility | | [72] |
| Greenhouse gas emission | | [73] |
| Growth and meat-production traits | | [74] |
| Response to gastrointestinal nematode infection | | [75] |
| Nematode resistance | | [76] |
| Body weight and nematode resistance | | [77] |
| Fat deposition | | [47] |
| Tail shape | | [39] |
| Wool-production traits | | [37] |
| Post-weaning body weight | | [78] |
| Eye muscle depth | | [79] |
| Heat stress | | [80] |
| Twinning rate | | [81] |
| Growth traits | | [82] |
| Body weight | | [78] |
| Prenatal lamb viability | | [65] |
| Carcass traits | | [33] |
|  | |  |
| Qualitative traits | |  |
| Double muscling | | [34] |
| Horn phenotype | | [34] |
| Horn phenotype | | [12] |
| Horn phenotype | | [18] |
| Horn phenotype – polycerate | | [83] |
| White vs. non-white coat color | | [54] |
| Dominant pigmentation | | [84] |
| Pigmentation in Merino sheep | | [85] |
| Prolificacy | | [86] |
| Behavioral traits | | [87] |
|  | |  |
| Health traits | |  |
| Susceptibility to ovine lentivirus infection | | [88] |
| Affected by microphthalmia | | [13] |
| Affected by BCRHS syndrome | | [32] |
| Affected by epidermolysis bullosa | | [89] |
| Affected by rickets | | [14] |
| Dagginess and host internal parasite resistance | | [90] |
| Affected by foot rot | | [91] |
| Paratuberculosis resistance | | [92] |
| Carrying junctional epidermolysis bullosa | | [93] |
| Affected by chondrodysplasia | | [94] |
| Resistance to nematodes | | [95] |
