# Supplementary material for: Unveiling genomic regions that underlie differences between Afec-Assaf sheep and its parental Awassi breed
Source: Genet Sel Evol. 2017 Feb 10;49:19. doi: 10.1186/s12711-017-0296-3 (PMC5301402; doi:10.1186/s12711-017-0296-3)
Supplement: Supplementary file 2 — Additional file 2: Table S2. Genomic relationship within and between local Awassi flocks. [file 12711_2017_296_MOESM2_ESM.docx]

**Table S2**. Genomic relationship within and between Local Awassi flocks

| Flock No. | Number of sheep | Type of comparison | Number of pairs compared | Mean | Min | Max |
| --- | --- | --- | --- | --- | --- | --- |
| 1 | 5 | Within flock | 10 | 0.14 | 0.13 | 0.17 |
|  |  | With other flocks | 95 | 0.13 | 0.10 | 0.23 |
| 2 | 4 | Within flock | 6 | 0.31 | 0.13 | 0.46 |
|  |  | With other flocks | 80 | 0.12 | 0.09 | 0.24 |
| 3 | 7 | Within flock | 21 | 0.26 | 0.11 | 0.64 |
|  |  | With other flocks | 119 | 0.13 | 0.09 | 0.34 |
| 4 | 2 | Within flock | 1 | 0.13 | 0.13 | 0.13 |
|  |  | With other flocks | 44 | 0.12 | 0.10 | 0.14 |
| 5 | 6 | Within flock | 15 | 0.15 | 0.08 | 0.44 |
|  |  | With other flocks | 108 | 0.13 | 0.09 | 0.34 |

Genomic relationship matrix (GRM) was created using Plink version 1.9 and the following options on the command line: --sheep --hwe 0.001 --geno --maf --make-rel.

The overall average coefficient value for within and between flocks genomic relationships were 0.20 and 0.13, respectively.
