# Supplementary material for: Unveiling genomic regions that underlie differences between Afec-Assaf sheep and its parental Awassi breed
Source: Genet Sel Evol. 2017 Feb 10;49:19. doi: 10.1186/s12711-017-0296-3 (PMC5301402; doi:10.1186/s12711-017-0296-3)
Supplement: Supplementary file 3 — Additional file 3: Table S3. Variable genomic regions (VGR) that differ between the Awassi and the Afec-Assaf according to PLINK and EMMAX analyses. SNPs were ranked according to their P-values, and the selected regions included the top 0.1% markers and their neighboring (1.5 Mb upstream and 1.5 Mb downstream) markers ranked in the top 1% of the markers. Two or more genomic regions were combined if they overlapped. Region position was determined using sheep genome assembly version 3.1. Regions identified by both analyses are marked in gray. [file 12711_2017_296_MOESM3_ESM.docx]

**Table S3.** Variable genomic regions that differ between the Awassi and the Afec-Assaf according to PLINK and EMMAX analyses

| OAR | **PLINK** | | | | | | **EMMAX** | | | | |
| --- | --- | --- | --- | --- | --- | --- | --- | --- | --- | --- | --- |
|  | **Position (Mb) According to OAR v3.1** | **No. of SNPs*** | **Peak SNP** | | **Position of peak SNP according to OAR v3.1** | **F*st*** | **Position (Mb) According to OAR v3.1** | **No. of SNPs*** | **Peak SNP** | **Position of peak SNP according to OAR v3.1** | ***P*-value** |
| 1 | 2.72 | 1 | | s01629.1 | 2,721,556 | 0.61 | 2.72 | 1 | s01629.1 | 2,721,556 | 1.0e-07 |
| 1 | 99.83-101.28 | 6 | | OAR1_107139545.1 | 99,832,319 | 0.65 | 99.83-101.29 | 4 | OAR1_107139545.1 | 99,832,319 | 5.4e-08 |
| 1 | 103.40-103.45 | 2 | | OAR1_111159099.1 | 103,452,513 | 0.60 | 103.40-103.45 | 2 | OAR1_111159099.1 | 103,452,513 | 2.5e-08 |
| 1 | 117.50-121.28 | 11 | | OAR1_130613822.1 | 120,440,651 | 0.57 |  |  |  |  |  |
| 1 | 164.60 | 1 | | s01145.1 | 164,604,667 | 0.59 | 164.60 | 1 | s01145.1 | 164,604,667 | 6.7e-07 |
| 1 | 175.56 | 1 | | s30872.1 | 175,562,170 | 0.46 |  |  |  |  |  |
| 1 | 239.61–239.72 | 2 | | OAR1_239608701.1 | 239,608,701 | 0.56 |  |  |  |  |  |
| 1 | 257.94 | 1 | | OAR1_278750895.1 | 257,942,931 | 0.55 |  |  |  |  |  |
| 1 | 266.24 | 1 | | OAR1_288212148.1 | 266,241,278 | 0.55 | 266.24 | 1 | OAR1_288212148.1 | 266,241,278 | 5.3e-07 |
| 2 |  |  | |  |  |  | 70.70 | 1 | OAR2_75333424.1 | 70,705,987 | 1.5e-06 |
| 2 | 76.33-77.82 | 2 | | OAR2_82988163.1 | 77,817,637 | 0.59 |  |  |  |  |  |
| 2 | 83.11-84.53 | 4 | | OAR2_88441803.1 | 83,214,641 | 0.59 | 83.21-84.53 | 4 | s14873.1 | 84,054,194 | 1.0e-05 |
| 2 |  |  | |  |  |  | 117.60–118.18 | 2 | OAR2_126354465.1 | 118,180,362 | 5.2e-06 |
| 2 | 148.40 | 1 | | s40283.1 | 148,406,594 | 0.68 | 145.65-148.41 | 2 | OAR2_154645235.1 | 145,648,041 | 3.9e-07 |
| 2 | 178.04-179.12 | 4 | | OAR2_189183764_X | 178,361,652 | 0.55 |  |  |  |  |  |
| 2 |  |  | |  |  |  | 194.08 | 1 | OAR2_205643974.1 | 194,084,442 | 1.4e-06 |
| 3 | 52.97 | 1 | | OAR3_56021384.1 | 52,976,986 | 0.58 |  |  |  |  |  |
| 3 | 134.23-134.48 | 3 | | s68368.1 | 134,234,489 | 0.66 | 134.23-134.49 | 2 | s68368.1 | 134,234,489 | 6.7e-07 |
| 3 |  |  | |  |  |  | 177.16 | 1 | AR3_190128314.1 | 177,157,519 | 1.2e-05 |
| 4 |  |  | |  |  |  | 100.88 | 1 | OAR4_107327897.1 | 100,878,221 | 2.7e-06 |
| 4 |  |  | |  |  |  | 117.31 | 1 | s60036.1 | 117,312,951 | 2.0e-06 |
| 5 | 1.66 | 1 | | OAR5_1793077.1 | 1,666,891 | 0.58 |  |  |  |  |  |
| 5 |  |  | |  |  |  | 76.93 | 1 | OAR5_84553099.1 | 76,934,651 | 1.5e-06 |
| 6 | 20.99-24.00 | 9 | | s27506.1 | 22,323,737 | 0.66 | 21.00-22.85 | 5 | s27506.1 | 22,323,737 | 5.1e-08 |
| 6 | 24.97-32.04 | 28 | | s24937.1 | 26,058,630 | 0.73 | 24.97–31.62 | 12 | s24937.1 | 26,058,630 | 1.8e-10 |
| 6 | 54.26-55.67 | 2 | | OAR6_59629965.1 | 54,261,462 | 0.55 | 54.26 | 1 | OAR6_59629965.1 | 54,261,462 | 5.8e-06 |
| 7 | 96.99 | 1 | | OAR7_105725274.1 | 96,995,379 | 0.58 | 96.99 | 1 | OAR7_105725274.1 | 96,995,379 | 2.9e-07 |
| 8 | 62.73-62.78 | 2 | | OAR8_67529714.1 | 62,735,336 | 0.57 | 62.62-62.83 | 5 | OAR8_67529714.1 | 62,735,336 | 6.8e-08 |
| 8 | 70.36 | 1 | | OAR8_75501256.1 | 70,361,288 | 0.59 |  |  |  |  |  |
| 8 | 73.54-75.47 | 3 | | s70959.1 | 74,292,186 | 0.59 |  |  |  |  |  |
| 8 | 78.36-80.60 | 3 | | s66278.1 | 78,364,891 | 0.67 | 80.60 | 1 | OAR8_86871896.1 | 80,605,889 | 5.2e-09 |
| 10 | 30.61-30.70 | 2 | | OAR10_30746533.1 | 30,700,853 | 0.80 | 27.65–31.64 | 11 | OAR10_30746533.1 | 30,700,853 | 8.4e-15 |
| 10 | 33.75-36.23 | 4 | | OAR10_36999163.1 | 36,238,012 | 0.66 | 33.75-36.24 | 5 | OAR10_34110882.1 | 33,752,924 | 1.2e-07 |
| 10 |  |  | |  |  |  | 56.53-58.12 | 4 | OAR10_57689327.1 | 56,534, 362 | 7.9e-06 |
| 10 |  |  | |  |  |  | 78.45 | 1 | OAR10_85663398.1 | 78,450,809 | 4.4e-06 |
| 12 | 58.25-58.98 | 2 | | OAR12_64628319.1 | 58,250,035 | 0.61 |  |  |  |  |  |
| 14 |  |  | |  |  |  | 26.43 | 1 | s10054.1 | 26,432,138 | 5.1e-08 |
| 17 | 8.13-8.74 | 2 | | OAR17_9701703.1 | 8,739,357 | 0.66 | 8.74 | 1 | OAR17_9701703.1 | 8,739,357 | 1.9e-06 |
| 17 | 45.64 | 1 | | s61559.1 | 45,647,697 | 0.56 |  |  |  |  |  |
| 19 |  |  | |  |  |  | 30.60-32.77 | 3 | OAR17_33278780.1 | 31,599,394 | 9.2e-06 |
| 19 | 53.20 | 1 | | s18532.1 | 53,198,678 | 0.56 |  |  |  |  |  |
| 20 | 37.51 | 1 | | OAR20_40967781.1 | 37,515,740 | 0.57 | 37.51 | 1 | OAR20_40967781.1 | 37,515,740 | 2.5e-06 |
| 21 |  |  | |  |  |  | 7.48-8.72 | 3 | OAR21_8817818.1 | 7,479,728 | 2.6e-06 |
| 23 | 17.31 | 1 | | OAR23_18329080.1 | 17,314,647 | 0.55 |  |  |  |  |  |
| 25 | 7.39-7.51 | 2 | | s25195.1 | 7,392,689 | 0.59 | 6.65-8.66 | 5 | s25195.1 | 7,392,689 | 4.7e-08 |
| 25 | 10.63 | 1 | | OAR25_10698230.1 | 10,627,436 | 0.62 |  |  |  |  |  |
| 26 | 18.54 | 1 | | OAR26_21898409.1 | 18,549,191 | 0.60 |  |  |  |  |  |
| 26 | 36.58 | 1 | | s03724.1 | 36,580,518 | 0.58 | 36.58 | 1 | s03724.1 | 36,580,518 | 5.4e-06 |

* Number of SNPs in the region from the 0.1% and the 0.5% cohorts.

SNPs were ranked according to their *P*-values, and the selected regions included the top 0.1% markers and their neighboring (1.5 Mb upstream and 1.5 Mb downstream) markers ranked in the top 0.5% of the markers. Two or more genomic regions were combined if they overlapped. Region position was determined using sheep genome assembly version 3.1. Regions identified by both analyses are marked in gray.
