# Supplementary material for: Unveiling genomic regions that underlie differences between Afec-Assaf sheep and its parental Awassi breed
Source: Genet Sel Evol. 2017 Feb 10;49:19. doi: 10.1186/s12711-017-0296-3 (PMC5301402; doi:10.1186/s12711-017-0296-3)
Supplement: Supplementary file 4 — Additional file 4: Figure S1. Different horn phenotypes in Awassi (A to D) and Afec-Assaf (E to H) sheep. (A) Polled, female. (B) Knobs, female. (C) Horns, female. (D) Horns, male. (E) Polled, female. (F) Knobs, male. (G) Knobs, male. (H) Scurs, male. [file 12711_2017_296_MOESM4_ESM.docx]

| 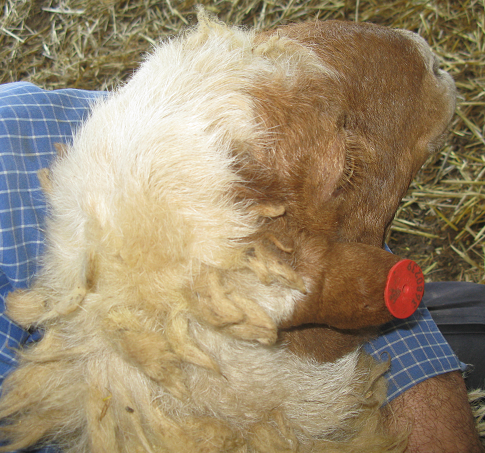 | 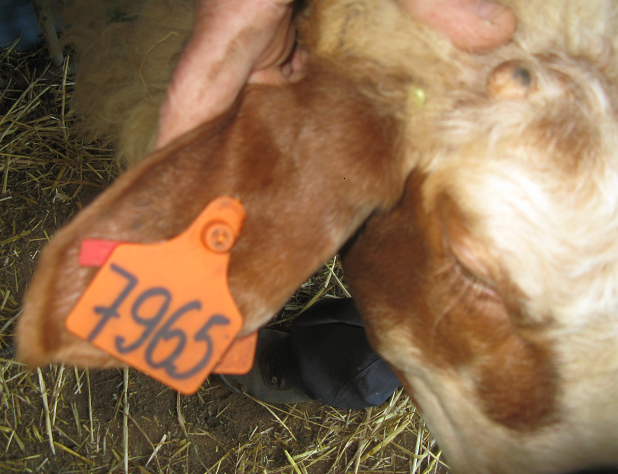 | 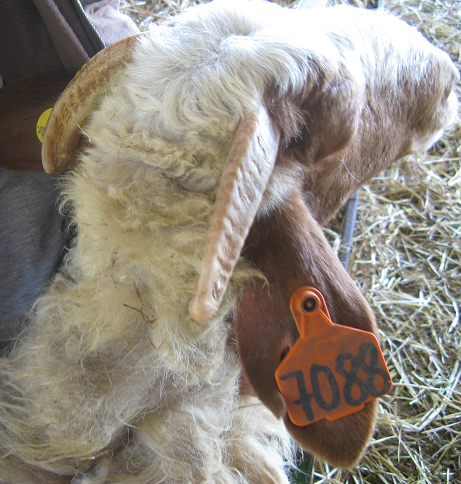 | 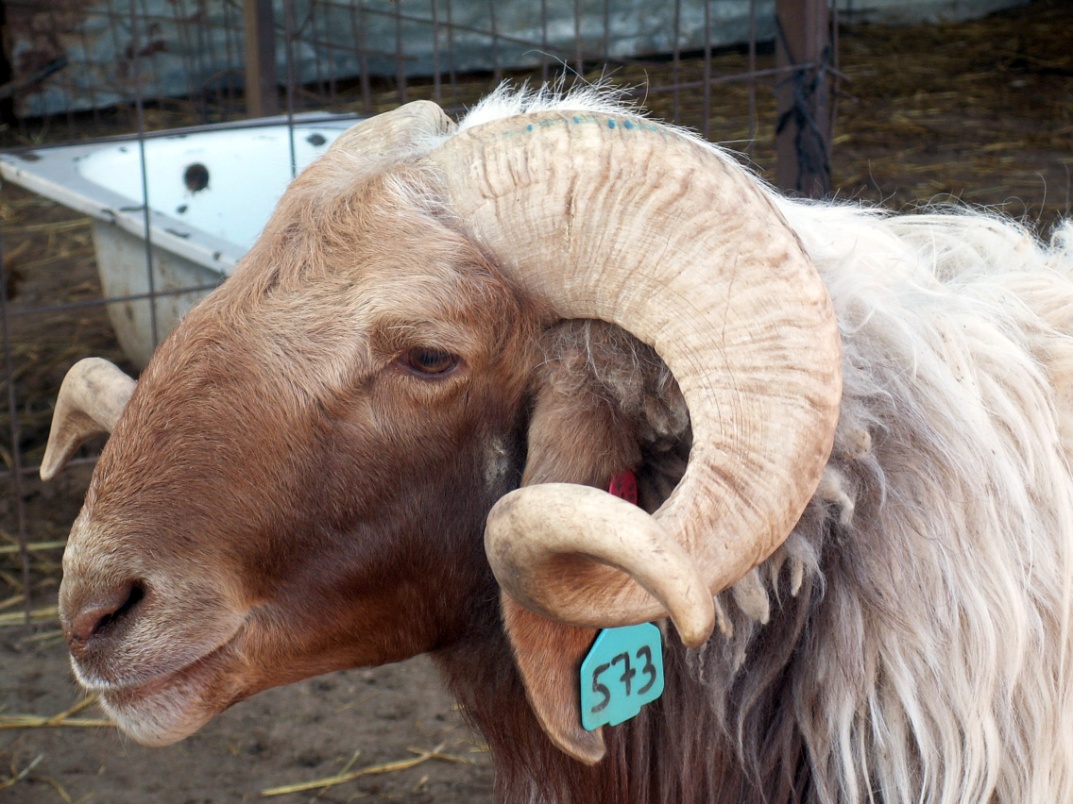 |
| --- | --- | --- | --- |
| A | B | C | D |
| 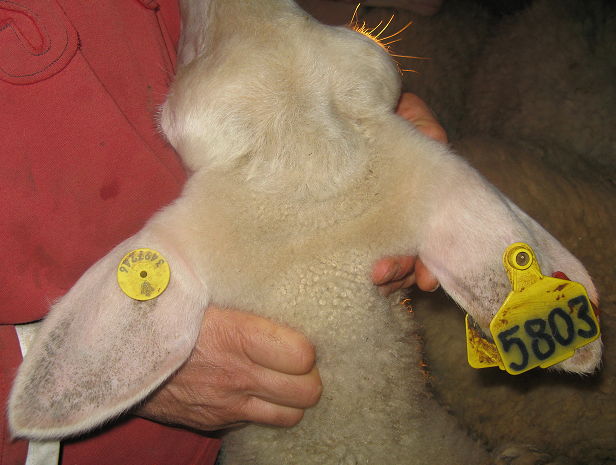 | 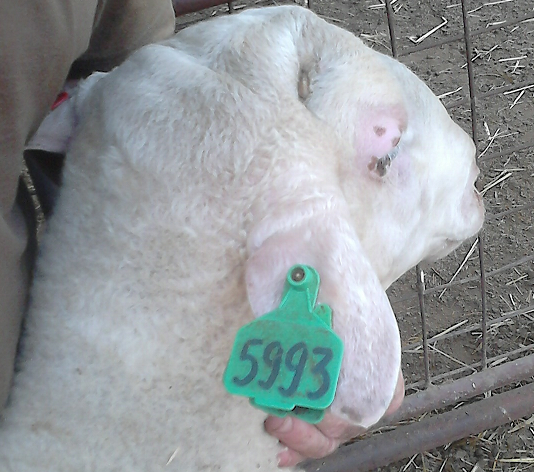 | 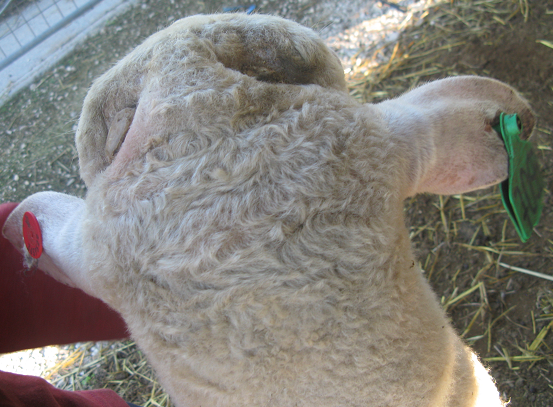 | 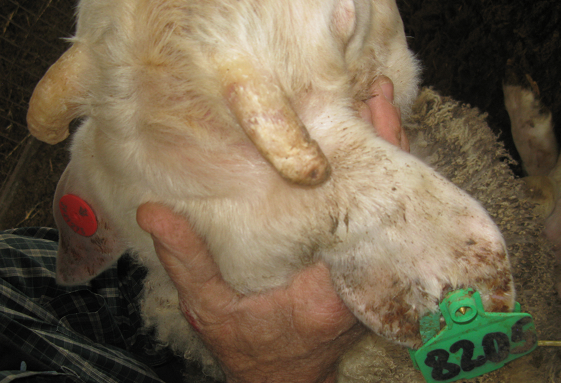 |
| E | F | G | H |

**Figure S1.** Different horn phenotypes in Awassi (A–D) and Afec-Assaf (E–H) sheep

(A) Polled, female. (B) Knobs, female. (C) Horns, female. (D) Horns, male. (E) Polled, female. (F) Knobs, male. (G) Knobs, male. (H) Scurs, male.
