# Supplementary material for: Unveiling genomic regions that underlie differences between Afec-Assaf sheep and its parental Awassi breed
Source: Genet Sel Evol. 2017 Feb 10;49:19. doi: 10.1186/s12711-017-0296-3 (PMC5301402; doi:10.1186/s12711-017-0296-3)
Supplement: Supplementary file 5 — Additional file 5: Table S4. Association between presence of a 1.8-kb insertion at the 3′-UTR of RXFP2 and horn phenotype in Awassi and Afec-Assaf sheep. A 428-bp PCR product indicates the presence of the insertion [19]. [file 12711_2017_296_MOESM5_ESM.docx]

**Table S4.** Gender-specific association between presence of 1.8-kb insertion at the 3’-UTR of the *RXFP2* gene and horn phenotype in Awassi and Afec-Assaf sheep

|  |  |  | Horn phenotype (n) | |
| --- | --- | --- | --- | --- |
| PCR product (bp) | Breed | Sex | Normal horns | Scurs, knobs, polled |
|  |  |  |  |  |
| 466/466 | Awassi | M | 5 | 0 |
|  |  | F | 4 | 0 |
|  | Afec-Assaf | M | 4 | 0 |
|  |  | F | 1 | 0 |
|  |  |  |  |  |
| 428/466 | Awassi | M | 5 | 2 |
|  |  | F | 0 | 5 |
|  | Afec- Assaf | M | 2 | 3 |
|  |  | F | 0 | 5 |
|  |  |  |  |  |
| 428/428 | Awassi | M | 0 | 3 |
|  |  | F | 0 | 1 |
|  | Afec-Assaf | M | 0 | 13 |
|  |  | F | 0 | 8 |
| **Totals** |  |  |  |  |
| 466/466 |  | F&M | 14 | 0 |
| 428/466 |  | M | 7 | 5 |
|  |  | F | 0 | 10 |
| 428/428 |  | F&M | 0 | 25 |

A 428-bp PCR product indicates the presence of the insertion, according to [19].
