# Supplementary material for: Unveiling genomic regions that underlie differences between Afec-Assaf sheep and its parental Awassi breed
Source: Genet Sel Evol. 2017 Feb 10;49:19. doi: 10.1186/s12711-017-0296-3 (PMC5301402; doi:10.1186/s12711-017-0296-3)
Supplement: Supplementary file 6 — Additional file 6: Table S5. Candidate genes for sheep coat colors and patterns [10, 11, 16, 34, 35, 38, 45, 52–54, 96, 97]. [file 12711_2017_296_MOESM6_ESM.docx]

**Table S**5**.**  Candidate genes for sheep coat colors and patterns

| Gene | | OAR | Position^*^ | References | Affiliation to a genomic region in the Awassi- Afec-Assaf GWAS (#) |
| --- | --- | --- | --- | --- | --- |
| Name | Symbol |  |  |  |  |
| *Tyrosinase-related protein 1* | *TYRP1* | **2** | 80,602,298 - 80,623,437 | [52-54] | Close to 6 |
| *Basonuclin 2* | *BNC2* | **2** | 84,290,339 – 84,688,738 | [11] | 6 |
| *KIT ligand* | *KITLG (MGF)* | **3** | 124,669,594 -124,762,432 |  |  |
| *Transcription factor SOX-10* | *SOX10* | **3** | 213,746,459 – 213,756,298 | [11] |  |
| *v-kit Hardy-Zuckerman 4 feline sarcoma viral oncogene homolog* | *KIT* | **6** | 70,189,729 - 70,234,612 | [54, 11, 10] |  |
| *Furry homolog* | *FRY* | **10** | 28,986,741- 29,188,660 | [35, 38, 45] | 15 |
| *Dopachrome tautomerase (tyrosine-related protein 2)* | *DCT (TYRP2)* | **10** | 69,452,774 – 69,492,896 | [54] |  |
| *Endothelin 3* | *EDN3* | **13** | 56,388,549 – 56,412,278 | [11] |  |
| Agouti signalling protein (*Aguti* locus) | *ASIP* | **13** | 63,237,431 - 63,242,627 | [54, 11, 10, 34, 96] |  |
| *Melanocortin 1 receptor* (*Extension* locus) | *MC1R* | **14** | 14,231,363 - 14,232,541 | [54, ,11, 16, 97] |  |
| *Micropthalmia transcription factor* | *MITF* | **19** | 31,583,798 - 31,811,540 | [54, 11, 34] |  |

*According to UCSC genome browser tool on ISAC Oar_v3.1 assembly (http://genome-euro.ucsc.edu/).
