# Supplementary material for: Unveiling genomic regions that underlie differences between Afec-Assaf sheep and its parental Awassi breed
Source: Genet Sel Evol. 2017 Feb 10;49:19. doi: 10.1186/s12711-017-0296-3 (PMC5301402; doi:10.1186/s12711-017-0296-3)
Supplement: Supplementary file 7 — Additional file 7. Candidate gene sequences. This file contains sequences of candidate genes in a FASTA-like format. Each sequence header consists of the gene symbol followed by its reference GenBank accession number and the breed name. Polymorphic nucleotide positions are indicated in blue and red font colors. [file 12711_2017_296_MOESM7_ESM.docx]

**Additional file 7**

File contains sequences of candidate genes in a FASTA-like format. Each sequence header consists of the gene symbol followed by its reference GenBank accession and the breed name. Polymorphic nucleotide positions are indicated by blue and red font colors.

>ALOX5AP_XM_004018330_AWASSI

TGTTTCTACTTGTTTCCTTGCACTGATATTATCATCCTGTAACTTGAGCTTCTAGAGAACTGAGGTCTTATCTGCCCACGCGATTTTGATTTGTGAGCCATCTCGCCGATTGCCCTGAGCACATGGGCTTTCATCACGAGCGCCGGGGTTGCAGCACACTCACTGCAGTGCTGCAGCCTCGGTTTTCTCACCCTCCAGTGGCATGAGGGTCCCTCTCTGTCCAGCTCAGTTCAGTGGGAAGCCACAGGCTGCTCATGAAGTCCTCCTCCTAATGGACGCTGAGGGCAACCTTGGCTGGAGAGCAGAAGGAAGAGACGTCATGAACCGGCAACACGCAGCTCGAGGGTTCTTTGCCCACAAGGTGGAGCACGAAAGCAAGACCCACAATGGGCGGAGCTTCCAGAGGACTGGGCCGCTGGCCTTTGAGCGGGTCTACACTGCCAACCAGAACTGTGTGGATGCCTACCCCACCTTCCTTGTCATGCTCTGGAG[T/C]GCCGGGCTTCTCTGCAGCCAAGTTCCTGCTGCCTTTGCTGGACTGATGTATCTGTTCGTGAGGCAGAAGTACTTTGTTGGCTACCTGGGGGAGAGAACTCAGAGCACACCTGGTTACATATTTGGGAAACGCATTATCCTGTTCCTATTCGCCATGTCCCTCGCTGGGATACTCAACTATTTCCTCATCGCCTTTTTCGGAAGTGACTTTGAAAACTACATCAAGACCGTCACCACCACCATCTCCCCTCTCCTTCTCATCCCTTGAGTCTCTGCTGAATTCCGGTTGGTGTGCTCAGCTAATCGATACCTAGAAGCCATCAAAGCGCAACCCTTTAAAAGAGGCTGTAAATCTAATGACCTGCTGGGCTTTGCAGCTTGAGTGAACCTTGCTTTTCCTGGAAGAGGAAATTTTCACATCAGCCTCGCCCTCTGAACAAGGCCAAGGCCCCACATTTGCAAATTCAGATCCCTTCTGTGTGTTTCGTGACTTATCCTGCTTTCTGAAGACGTTTTGTGACCCAGTTTATGTTTTCCTAAAATAAAACACAGACATATGTTTGAGGCTGG

>ALOX5AP_XM_004018330_Afec ASSAF

TGTTTCTACTTGTTTCCTTGCACTGATATTATCATCCTGTAACTTGAGCTTCTAGAGAACTGAGGTCTTATCTGCCCACGCGATTTTGATTTGTGAGCCATCTCGCCGATTGCCCTGAGCACATGGGCTTTCATCACGAGCGCCGGGGTTGCAGCACACTCACTGCAGTGCTGCAGCCTCGGTTTTCTCACCCTCCAGTGGCATGAGGGTCCCTCTCTGTCCAGCTCAGTTCAGTGGGAAGCCACAGGCTGCTCATGAAGTCCTCCTCCTAATGGACGCTGAGGGCAACCTTGGCTGGAGAGCAGAAGGAAGAGACGTCATGAACCGGCAACACGCAGCTCGAGGGTTCTTTGCCCACAAGGTGGAGCACGAAAGCAAGACCCACAATGGGCGGAGCTTCCAGAGGACTGGGCCGCTGGCCTTTGAGCGGGTCTACACTGCCAACCAGAACTGTGTGGATGCCTACCCCACCTTCCTTGTCATGCTCTGGAGCGCCGGGCTTCTCTGCAGCCAAGTTCCTGCTGCCTTTGCTGGACTGATGTATCTGTTCGTGAGGCAGAAGTACTTTGTTGGCTACCTGGGGGAGAGAACTCAGAGCACACCTGGTTACATATTTGGGAAACGCATTATCCTGTTCCTATTCGCCATGTCCCTCGCTGGGATACTCAACTATTTCCTCATCGCCTTTTTCGGAAGTGACTTTGAAAACTACATCAAGACCGTCACCACCACCATCTCCCCTCTCCTTCTCATCCCTTGAGTCTCTGCTGAATTCCGGTTGGTGTGCTCAGCTAATCGATACC[C/T]TAGAAGCCATCAAAGCGCAACCCTTTAAAAGAGGCTGTAAATCTAATGACCTGCTGGGCTTTGCAGCTTGAGTGAACCTTGCTTTTCCTGGAAGAGGAAATTTTCACATCAGCCTCGCCCTCTGAACAAGGCCAAGGCCCCACATTTGCAAATTCAGATCCCTTCTGTGTGTTTCGTGACTTATCCTGCTTTCTGAAGACGTTTTGTGACCCAGTTTATGTTTTCCTAAAATAAAACACAGACATATGTTTGAGGCTGG

_______________________________________________________________________________________

>FRY_XM_012185002_AWASSI

GACCGGCGGCCGGCGTCGCTGGCACTGGCAGACGCCTCCCAGGGCTAGAGATCCGCACCCAGCGAATAAGCAGCCAGTCCTGGACCGGGAGGAGGAGCGGCCGAGCATCCCTCTCCCGCTCCGCCGCGTCCTTCTCATGAGGACTCGCCGGAGGTGGACCCGCAGAGCCAGCCTCCCGGCCGCTGACCCCGCTTACATCACGAACCTGTGACCAGCGCATCCCTCCCACGCCCGGTACCTGCTCCGCGCGGCTCACGGTCCAGCCCCGGGAGCAGGCTCTTTGTATGCCGCCGACATGGCCAGCCAGCAGGATTCCGGCTTCTTCGAGATCAGTATCAAATATTTACTGAAATCCTGGAGTAATACTTCTCCAGTTGGCAACGGTTATATCAAGCCTCCAGTTCCACCTGTGTCTGGCGCACAGAAGGAGAAAGGGCCCCCAGCCATGTTACCCATCAGTGTGGACCCAGACAGCAAACCCGGGGAATACGTCCTCAAGAGTTTATTTGTGAACTTTACCACTCAGGCTGAGCGCAAGATTCGCATCATTATGGCAGAACCCCTGGAAAAGCCATTGACAAAATCTCTACAACGTGGAGAAGACCCCCAGTTTGATCAAGTCAT[C/T]AGTTCAATGAGCTCCCTTTCTGAATACTGCCTGCCTTCCATTCTACGTACATTATTTGACTGGTATAAAAGGCAAAATGGCATTGAGGATGAATCACATGAGTACAGACCAAGAACCAGCAATAAATCCAAAAGTGATGAACAGCAGCGAGATTATTTAATGGAAAGACGGGACCTCGCCATTGATTTTATTTTTTCTTTAGTATTAATAGAAGTTTTGAAACAGATTCCACTTCATCCTGTAATAGACAGTTTAATACATGATGTTATTAACTTGGCTTTCAAGCACTTTAAGTACAAAGAAGGGTACCTTGGTCCTAACACTGGCAATATGCACATTGTGGCAGACCTGTATGCAGAAGTCATTGGCGTGTTGGCACAAGCTAAATTCCCTGCTGTAAAGAAGAAATTTATGGCAGAGCTCAAAGAATTACGGCACAAAGAGCAGAGCCCATATATTGTTCAAAGCATCATCAGCTTGATAATGGGGATGAAGTTCTTTCGAATTAAGATGTATCCAGTGGAGGACTTTGAGGCCTCTCTTCAGTTTATGCAGGAATGTGCACATTACTTTCTTGAGGTCAAAGACAAAGATATCAAGCATGCCTTGGCTGGGCTGTTTGTTGAAATTCTTGTCCCAGTGGCTGCTGCTGTTAAAAATGAAGTTAATGTTCCCTGCCTCAGAAACTTCGTTGAAAGCCTGTATGATACCACGCTGGAACTTTCTTCTCGAAAGAAGCATTCCTTGGCCTTGTACCCACTGGTGACCTGTCTGCTCTGCGTCAGCCAGAAACAGCTATTCCTGAACAGGTGGCACGTTTTCCTCAACAACTGCTTATCCAACCTTAAAAATAAAGACCCCAAGATGGCTCGAGTTGCACTGGAATCTCTGTACAGATTACTCTGGGTTTACATGATCCGAATTAAATGTGAAAGCAACACGGCTACTCAGAGCCGTCTTATAACCATCATCACAACACTTTTCCCCAAAGGGTCCCGTGGTGTGGTGCCAAGGGATATGCCTCTCAACATCTTTGTGAAAATCATCCAGTTCATCGCCCAGGAACGTTTAGATTTTGCAATGAAAGAAATCATTTTTGATTTCCTTTGTGTGGGAAAACCAGCAAAAGCTTTCAGTCTCAATCCAGAGAGAATGAATATTGGCTTAAGGGCGTTCTTGGTCATAGCCGACAGCTTGCAGCAGAAAGATGGAGAACCTCCCATGCCGGTTACCGGGGCTGTTCTCCCTTCAGGAAACACACTGAGGGTGAAGAAAAC[G/A]TATCTGAGTAAGACACTAACTGAAGAGGAAGCCAAAATGATAGGCATGTCATTATATTACTCTCAAGTACGAAAAGCTGTGGACAACATTTTAAGGCACCTTGATAAAGAAGTAGGAAGGTGTATGATGCTGACTAACGTCCAGATGTTAAACAAAGAACCCGAAGACATGATCACGGGTGAGAGAAAGCCAAAAATAGATCTTTTCAGGACCTGTGTTGCTGCTATTCCTCGACTGCTTCCTGATGGGATGTCAAAACTTGAACTTATTGACCTGCTGGCGAGGCTCTCTATTCATATGGATGATGAACTGCGACATATTGCCCAAAATTCTCTTCAGGGTTTACTTGTCGACTTCTCAGACTGGAGGGAAGATGTCCTGTTTGGTTTCACCAACTTCCTGCTTCGGGAAGTAAATGACATGCATCACACGCTCCTCGACTCGTCTCTCAAGCTCCTGCTCCAGCTGCTCACCCAGTGGAAACTGGTCATACAAACGCAAGGCAAAGTCTACGAACAGGCCAACAAAATCAGAAATGCAGAG[C/G]TAATTGCCAATGGCTCCAGCCACCGAATCCAGTCGGAGCGAGGTCCCCACTGCAGTGTACTCCATGCTGTGGAAGGTTTTGCCCTGGTTTTACTCTGCAGTTTCCAGGTGGCCACACGCAAACTGTCTGTTTTAATACTCAAGGAAATTCGAGCTTTATTTGTTGCTCTGGGTCAGCCTGAGGACGATGACAGACCTATGATTGATGTCATGGATCAGCTGAGTTCTTCCATCCTCGAAAGTTTTATTCACGTAGCAGTTTCGGATTCAGCGACCCTGCCCTTGACCCACAGTGTGGACCTGCAGTGGCTGGTGGAGTGGAGTGCTGTCCTGGTCAACAGCCATTACGACGTGAAGAGCCCTTCCCACGTCTGGATTTTCGCCCAGTCTGTCAAGGATCCCTGGGTGCTCTGCCTCTTCAGCTTCCTCCGGCAAGAGAACCTGCCCAAGCACTGCCCCACTGCCCTCAGCTATGCCTGGCCCTACGCCTTCAC[A/G]CGGCTGCAGTCTGTGATGCCCCTGGTGGACCCGAATAGCCCAATTAATGCCAAGAAAACCAGCAC[G/A]GCCGGCAG[C/T]GGAGACAACTACGTTACGTTGTGGAGAAATTACCTTATTCTTTGCTTTGGAGTTGCAAAACCCAGTATTATGAGCCCAGGACACTTACGAGCTTCCACTCCAGAAATAATGGCCACGACACCTGATGGTACAGTGAGCTACGATAACAAGGCCATAGGCACCCCGTCGGTGGGCGTTCTGTTAAAGCAGTTGGTGCCTTTGATGAGACTTGAGGGCATTGAGATCACAGAGTCCTTAGTTTTAGGATTTGGAAGGACAAATTCCCTTGTCTTCAGAGAATTGGTAGAAGAACTTCATCCGTTAATGAAAGAGGCTCTGGAACGAAGGCCAGAGAACAAAAAACGCCGAGAACGGCGAGACTTGTTAAGGCTACAGCTGCTTCGAATTTTTGAACTTCTGGCTGATGCTGGGGTCATAAGTGACAGCACCAATGGAGCCTTAGAACGGGACACTTTAGCCCTGGGAGCTTTGTTCTTAGAATACGTGGACCTGACCCGCATGCTCTTAGAAGCCGAAAATGATAAAGAAGTGGAAATTCTTAAAGATATCCGGGCACATTTTAGTGCCATGGTTGCCAACTTGATTCAGCGTGTTCCAGTTCACCACCGAAGATTTCTCTTCCCCCAGCAAAGCCTGAGGCATCATCTTTTCATCTTATTTAGCCAGTGGGCAGGACCCTTCAGCATTATGTTCACTCCCTTGGATCGTTACAGTGACAGAAATCATCAGATTACAAGATACCAGTATTGTGCGTTAAAGGCAATGTCAGCAGTGTTGTGCTGTGGCCCGGTGTTTGACAACGTGGGCCTTTCCCCAGATGGCTACCTATACAAGTGGCTTGACAACATTCTGGCTTGTCAAGATTTACGAGTTCATCAGCTTGGCTGCGAGGTTGTC[G/A]TCTTGCTACTGGAACTAAATCCTGACCAAATAAATCTTTTCAACTGGGCGATTGACCGGTGCTACACTGGTTCCTACCAACTTGCGTCCGGTTGCTTCAAGGCCATAGCTACTGTGTGTGGAAGCAGGAACTATCCCTTTGACATAGTGACGCTGTTAAACCTTGTTCTGTTCAAGGCCTCTGACACCAACAGAGAGATTTATGAAATCTCCATGCAGCTCATGCAGATCCTTGAAGCAAAGCTTTTTGTGTACTCGAAGAAAGTAGCCGAACAAAGACCTGGCAGTATCCTGTATGGGACGCACGGCCCACTGCCACCCCTCTACAGCGTGTCGCTGGCCTTCTTGTCGTGCGAGCTGGCCAGGATGTACCCCGAGCTCACTCTTCCTCTCTTCTCAGAAGTCAGCCAGCGCTTCCCCACAACGCATCCCAACGGGCGTCAGATCATGCTCACCTACCTGCTGCCCTGGCTGCACAACATCGAGCTGGTGGACAGCAGGCTGCTCCTCCCGGGGTCCAGCCCCAGCAGCCCCGAGGACGATGCCAAGGACCG[C/T]GAGGGAGACATGAC[G/A]GCTTCTCAGGGGCTGAAAGGCAGTGGCTGGGGCTCCCCGGAGGCGACGTCCCTGGTGCTGAACAACCTCATGTACATGACGGCCAAGTATGGGGATGAAGTTCCTGGACCGGAGATGGAAAATGCCTGGAATGCATTAGCTAACAACGAGAAATGGAGCAACAACCTGCGGATCACCTTGCAGTTCCTGATCAGCCTGTGCGGGGTCAGCAGCGACACGCTCCTCCTGCCCTATATCAAGAAGGTGGCCGTCTACCTGTGCCGGAACAACACCATTCAGACCATGGAGGAGCTGCT[G/C]TT[C/T]GAGCTGCAGCAGACGGAGCCGGTGAACCCCATTGTCCAGCACTGCGACAACCCGCCCTTTTACCGCTTCACAGCCAGCAGCAAGGCCTCGGCAGCGGCCTCCGGAACCACCTCCAGCAGCAATACAGTGGTTGCCGGCCAGGACAGTTTTCCAGACGCTGAGGAGAGCAAGATACTGAAAGAATCTGATGAGAGGTTTAGTAACGTCATCAGAGCCCATACGCGTCTGGAGTCAAGATACAGCAACAGCTCAGGAGGCTCATACGATGAAGATAAAAATGACCCAATTTCTCCCTACACCGGCTGGCTTCTGACTATCACAGAGACCAAACAGCCGCAGCCCCTACCCATGCCTTGCACGGGAGGGTGCTGGGCCCCCCTGGTGGACTACCTCCCAGAGACCATCACCCCCCGGGGGCCGCTCCACAGGTGCAATGTTGCTGTCATCTTTATGACTGAGATGGTGGTGGACCACAGTGTCAGAGAGGACTGGGCTCTCCATCTCCC[A/T]CTGCTGCTCCATGCTGTCTTCCTAGGTGGCTTCGACTTCCTGAGAGAGGATCAGTCATCCCCGGTGCCAGACTCAGGGCTCAGTTCCAGCTCCACTTCATCCAGCATCAGTCTGGGAGGCAGTAGTGGGAACCTCCCACAGATGACCCAGGAGGGAGAAGATGCAGAC[G/T]CGGCTGCCGACTCAGATGAGAAGGCAAACAAGCTCATTGAGTTTCTGACGACCAGGGCATTTGGTCCACTTTGGTGCCATGAGGACATCACACCCAAAAATCAGAATTCAAAGAGTGCTGAACAACTCACCAATTTTCTACGTCATGTGGTATC[C/T]GTGTTCAGAGATTCCAAATCAGGTTTCCACCTGGAGCAGCACCTGAGTGAAGTGGCGCTGCAGACGGCCCTCGCCAGCTCTTCCCGGCACTATGCCGGCCGGTCCTTCCAGATATTCCGGGCCCTCAAACAACCTCTGTCAGCACACGCCTTGTCGGACCTCCTCTCCAGGCTGGTGGAGGTGAT[T/C]GGCGAGCACGGAGATGAGATTCAGGGCTATGTCATGGAAGCCCTTCTTACCCTGGAAGCTGCCGTGGACAACTTGTCTGACTGCTTGAAGAACACTGATCTCTTAACTGTACTGTCTCGCTCTTCATCACCAGACTTAAGCTCTAACACTAAACTGACAGCTAGCAGAAAGAGCACAGGACAGCTCAATGTGAACCCTGGGGCAGCTAGCGGCAACACAGCAACTGCAGAACGCAGCCGGCATCAAAGGAGCTTCTCTGTGCCCAAGAAGTTCGGTGTTGTTGACCGGTCCTCAGACCCACCTCGGAGTGCCACCCTGGACAGAATTCAGGCCTGTACCCAACAAGGCCTCTCCTCAAAAACCAGAAGCTCATCCTCCTTGAAGGACAGCCTCACTGACCCATCACACATAAACCATCCAACCAACCTGTTGGCCACCATCTTTTGGGTCACGGTGGCTTTGATGGAATCCGATTTTGAGTTTGAATACCTAATGGCCCTGAGGCTGTTGAACAGACTGCTGGCTCACATGCCACTTGATAAAGCTGAGAACCGAGAAAAGCTAGAGAAACTCCAGGCGCAGCTCAAGTGGGCAGACTTTTCAGGGTTGCAGCAGCTGCTGTTGAAGGGGTTCACCTCCCTCACCACCACCGACCTTACCTTGCAGCTGTTCAGTTTGCTGACACCAGTGTCCAAAGTATCCATGGTGGACTCATCTCAAGCCATTGGGTTTCCACTGAATGTCTTATGTCTTCTGCCCCAGCTGATACAGCATTTTGAAAACCCCAACCAGTTCTGTAAGGATGTAGCTGAGAGAATCGCTCAGGTTTGTTTGGAAGAGAAGAACCCCAAACTTTCCAATCTTGCACACGTCATGACTCTTTATAAAACACACAGCTACACGAGGGACTGTGCCACGTGGGTCAATGTGGTCTGCCGGTACCTTCATGAAGCCTATGCTGACATCACCTTGAATATGGTTACCTACCTGGCAGAGCTTCTGGAGAAGGGCCTCCCCAGCATGCAGCAGCCCCTCCTGCAGGTGATCTACAGCCTGCTCAGCTACATGGACCTTTCTGTCGTTCCTGTAAAGCAGTTCAACGTGGAAGTTCTGAAGAC[A/C]ATTGAAAAATACGTGCAAAGCATTCACTGGAGAGAAGCTTTGAATATTTTGAAGCTGGTGGTTTCTCGATCTGCCAGCCTAGTTTTGCCTTCCTACCAACACAGTGACCTCTCAAAGATAGAAATACATCGAGTGTGGACGAGCTCATCCAAGGAATTACCTGGAAAAACCCTAGACTTCCACTTTGATATTTCAGAGACGCCTATCATCGGGAGGCGGTATGATGAACTGCAGAACTCCTCTGGGCGTGACGGGAAGCCCAGGCCCATGGCCGTCACCCGGAGTGCATCCTCCACCTCCTCGGGCTCCAACTCCAACGTCCTTGTCCCTGTCAGCTGGAAAAGGCCCCAGTACTCCCAGAAGAGGACCAAAGAGAAGTTGGTGCACGTCCTTTCTCTGTGTGGCCAGGAAGTAGGATTAAGCAAGAATCCATCAGTGATCTTCTCGTCGTGCGGGGACCTGGACCTGCTGGAGCATCAGACGAGCCTGGTGTCATCCGAGGACGGGGCCCGGGAGCAGGAGAACATGGATGACACCAACAGCGAGCAGCAGTTCCGGGTCTTCAGAGACTTCGACTTCCTGGACGTGGAGCTAGAGGACGGAGAGGGCGAGAGTATGGACAACTTCAACTGGGGAGTCCGCAGGCGTTCTTTGGACAGTCTGGACAAGTGTGACCTGCAGCTGCTGGAGGAGCGCCAGCTCTCAGGAAGCTCTCCCAGCCTCAATAAAATGAACCACGAGGATTCTGATGAGTCCTCTGAGGAGGAGGACCTCACCACCAGCCAGATCCTGGAGCACTCTGACCTCATCATGAATCTCTCTCCTTCGGAGGAGACTAACCCCATGGAATCACTCACGACCGCCTGCGATCTGACCCCTGCTGACCCTCACTCCTTTAACACCAGGATGGCTAGCTTTGAAGCTGCTTTGCCTGATATGAATAATCTTCAGATTTCTGAGGGTTCAAAGGCCGATGCCGTCCAGGAAGAGGAGGACGCCACTGTGCAGGAGGATGACCTTTCCAGCTCCATCAATGAACTCCCAGCCACTTTCGAATGCAGCGACAGCTTTAGCCTGGATATGACTGAGGCAGAAGAAAAAGGCAGTCGAGCGCTGGACCACTTCACTCTTGCCAGCTTCGGGGAAGGTGAGAGGGGAGTGTCGCCCCCTCCCTCGCCCTTCTTCTCGGCCATCCTTGCCGCCTTTCAGCCCACGGCCTGTGACGACGCCGAGGAGGCCTGGCGCAGCCACATCAACCAGCTCATGTGCGACTCAGACGGCTCCTGTGCTGTGTACACATTCCACGTGTTCTCCTCCTTGTTTAAGAATATTCAGAAAAGGTTCTGCTTCCTAACCTGTGATGCAGCCAGTTACCTTGGTGATAACCTCCGGGGCATTGGATCCAAGTTTGTCAGCTCTTCCCAGATGCTTACCTCCTGCTCCGAGTGCCCCACGCTCTTCGTGGATGCCGAGACCCTCCTTTCTTGTGGACTTCTGGACAAGCTCAAGTTCAGTGTTTTAGAACTGCAAGAATATTTGGATACCTACAACAACAGAAAAGAGGCCACTCTCTCATGGCTTGCAAATTGTAAGGCGACATTTGCAGGAGGATCAAGAGATGGAGTAATTACCTGTCAACCAGGGGACTCAGAGGAAAAGCAACTGGAACTGTGTCAGAGGTTGTATAAGCTACACTTCCAGCTGCTGCTGCTTTTTCAGTCCTACTGTAAGCTCATCGGCCAGGTGCACGAAGTCAGCTCCATGCCAGAGCTGCTAAATATGTCCAGGGAACTGAGTGACCTAAAGAAAAACCTGAAGGAGGCCACTGCAGCCATTGCAGCTGACCCTCTTTACATCGAGGGGGCGTGGTCCGAGCCAACCTTCACATCCACTGAGGCAGCCATCCAGTCCATGCTTGAGTGCTTGAAGAACAACGAACTTGGCAAGGCTTTACGGCAAATAAGGGAATGCAGAAGTCTGTGGCCCAATGACATCTTTGGAAGCAGTTCTGACGATGAGGTCCAGACACTGCTGAATATTTATTTCCGCCATCAAACTCTAGGACAGACGGGCACTTACGCATTGGTGGGGTCTAACCAGAGCCTGACTGAGATCTGCACCAAGCTGATGGAGCTGAACATGGAGATCCGGGACATGATCCGCAGGGCCCAGAGCTACCGGGTCCTCACCGCCGTCCTTCCCGACTCCAGCGTGTCCGGCACTAGCCTCTGACAGGAGCCTCCCATCCCCCATGGGCGCCACGCTGGGGGTGCTGCCGTGCTGGGGTGACGTCATTCAGTGTCTTCTCAGCCTTCAAAAGGCTTGGTCAGACTGTTCTCCCTCTTGTTGCCTGTAGGACTTTTCATAAAGAGGATGGCAGAACTTCCCACGTGTAGCAATACTCTTAAGAACCAAGGTAGCATAGGACTGCTCTGGAACAGCCTCCATCCATCATCCTGTGTGACACAAACGCATTATCCTTTTACTGAGCAAAAACTCACTACTGAAGGCGTGACTTCCATTGCATACCAAAGCCAACTACTCTGAACAATACCTTCCTTTCTAGAAACAATTTTAGATTGGCAAAAGTGCAATGTTTTCTTCACTAAAAAATATTTTATATTCTAAAACTTGTACATTCTTTTTCATTTTTCTCTGCCTTTGTTTTGTGTGTGTGTGTGTGAGGTGAGCTGAGGAAGGAGCAGGCAGAATGAAGCTGGCCATGGAAAACTGTAAGACGGTCAAAAGCTGACAGCCTGTGTATGTGAAAAGAGAACTGTAAATGGACAATTCAATGTACACTGAAATTTGAATACCATTACTGTGTCTAAAAGGAGCTGCTATGAAGTACCTTTCTTATGTTGCTAGGCTACTGTCTCGGAAAGCCCTGGATCTCTTTGCAGGGGTCTGTGATTAAGACCCTCGCACCAAAAATGGTCCAGACAGACATTTTTTTAAGGATCTTGGCTGCTTTTTATTAGAATGTTGCTTTTATGAACATATTTATACTACAGAAGGATGAGTGTTAATTTTAACCAACTTTGCCATTTTATAGAATAAAGTTAAAGACGTCCACATTATAAATTCCAAGTCTTTCCACCTGTTACGCATGCATATTTCAATATCCACGTGGATAGCTGGAAATAGGACATAAAGGTAAATATGAGTTGTCACTGTTTCTTAAGAAATGTCATTTTATCTGTAATATATATGTAAAGGGCCATTCTTAAGTTTTCCCCTTAAATTTAATGCTGTCAAGTGTTAGCTGTGTGCATGTAAACCTGTTGCACATCAGAAGCATATTCAAAGTTTATCTATGTAACTTATTCACTCTGTAAATACATTTAAAGTTTTTGTGATGTAATCTCGGTTGACATTCTGTTTAGAACTGCCTTTAAAGTAAAAAAAAAAAAAATCATACTGACCACAGGTTTTATTTCTATTTAATGAAGATTTTTGCTTTGATTTATATTTTTGCATGTGGGTTGCCATGATAACAACCCCAAGGAGTACCACACGCGTGTGTTCCATGACTGGCAGCCAGGGGGTTGTGAAGCAAGTACCATGACCCTGGGAAGCAGGAGGTCTCTGTAACCTCTTAAGATGGACACCAAAGACTGTAACCCTCAACCAGCTCTCACTATAATCAGAAGCAAGAAACCAGAAGACAAGAGCTCTCATTATCCACAGCCTATCATTAGGTTTTCATTCTTAAAATTTTGAAAGAGAACCATTAATGATAAGGAAAAAGTAGTGAGTTCTATTCTTTCACCACAACCCATCTATTCTTCACCCAACTCAAAAACGGGATCATGGAAACACACAACAATCTAATTTCTATGGTCTTCCTATTCATACTCCTTAGATTAACCAACGTAACTTGCTTCCTAATATACAACTAAATCAGAAGTAGTAATTTTCTAGACGTATCCACAACAGAGTCTGATCCAAGATTTTAATTTTACCACAGATACAATTATTACCTGGGTTTTAATGACTTTCTGGACATCGCTGTTAACATACTATTTTGATGATTTTCAACTTACCCTAAAGGTGATTAACCATAATCTGTAGGAAAAAACTGAATCACAATCTCTCTGGGGGGAAAAATTTTTCACAAAATGCAGGTTTCTGTGGTTAACAACACTAGGCAACTCTGAGTTCTGCTGGGACCCCCACTATAAAGGACTGATTAGTCTTGACTGAATTTTCCACCATGGCCTCTTACCCAGCAAAGAACCACCATGATTGGGTTATTCTTTTAACAAGAGTGTTTTTGAGTGATATCATCTGTAGTCATGAGAAGCAGTGGGGTTTTTTTTTTCTGTTTTCTTATTTTTTTTAAACAGGTTAATGTGAAAGAGTTCTCCTTTTCCTGTTTCTTAACCTCCTCTCCCTTAGTGAAGCAGAAGGGCCCTCTGAGGCTGCAGACAGGCCCCAGAGGAGCCCAACAATCAGTGACTTGAGAGCTGCCCACAAGCCTCCTGGGGTATCGGGTTCTCCCTCTGTTACTGAAGCCTTAATGTTCACTTCCCCAGGGGCAGGCACAGGAGGACGAGTAGGATATCTGCCCTCTCCTTTAGATGCCTATCTCCTCGCCTCTGAGGAGGAACACCTCCTCCTACCACCACGCTTTTTAGCCCTAGAGTGTCACACACTTTATCCACTGGCAAAAAAAAAAAAGCAATTCAGGCAGCTGTACTGCAACTT

>FRY_XM_012185002_Afec ASSAF

GGGCCGAGCGTGTGGGCGACCGGCGGCCGGCGTCGCTGGCACTGGCAGACGCCTCCCAGGGCTAGAGATCCGCACCCAGCGAATAAGCAGCCAGTCCTGGACCGGGAGGAGGAGCGGCCGAGCATCCCTCTCCCGCTCCGCCGCGTCCTTCTCATGAGGACTCGCCGGAGGTGGACCC[G/A]CAGAGCCAGCCTCCCGGCCGCTGACCCCGCTTACATCACGAACCTGTGACCAGCGCATCCCTCCCACGCCCGGTACCTGCTCCGCGCGGCTCACGGTCCAGCCCCGGGAGCAGGCTCTTTGTATGCCGCCGACATGGCCAGCCAGCAGGATTCCGGCTTCTTCGAGATCAGTATCAAATATTTACTGAAATCCTGGAGTAATACTTCTCCAGTTGGCAACGGTTATATCAAGCCTCCAGTTCCACCTGTGTCTGGCGCACAGAAGGAGAAAGGGCCCCCAGCCATGTTACCCATCAGTGTGGACCCAGACAGCAAACCCGGGGAATACGTCCTCAAGAGTTTATTTGTGAACTTTACCACTCAGGCTGAGCGCAAGATTCGCATCATTATGGCAGAACCCCTGGAAAAGCCATTGACAAAATCTCTACAACGTGGAGAAGACCCCCAGTTTGATCAAGTCAT[C/T]AGTTCAATGAGCTCCCTTTCTGAATACTGCCTGCCTTCCATTCTACGTACATTATTTGACTGGTATAAAAGGCAAAATGGCATTGAGGATGAATCACATGAGTACAGACCAAGAACCAGCAATAAATCCAAAAGTGATGAACAGCAGCGAGATTATTTAATGGAAAGACGGGACCTCGCCATTGATTTTATTTTTTCTTTAGTATTAATAGAAGTTTTGAAACAGATTCCACTTCATCCTGTAATAGACAGTTTAATACATGATGTTATTAACTTGGCTTTCAAGCACTTTAAGTACAAAGAAGGGTACCTTGGTCCTAACACTGGCAATATGCACATTGTGGCAGACCTGTATGCAGAAGTCATTGGCGTGTTGGCACAAGCTAAATTCCCTGCTGTAAAGAAGAAATTTATGGCAGAGCTCAAAGAATTACGGCACAAAGAGCAGAGCCCATATATTGTTCAAAGCATCATCAGCTTGATAATGGGGATGAAGTTCTTTCGAATTAAGATGTATCCAGTGGAGGACTTTGAGGCCTCTCTTCAGTTTATGCAGGAATGTGCACATTACTTTCTTGAGGTCAAAGACAAAGATATCAAGCATGCCTTGGCTGGGCTGTTTGTTGAAATTCTTGTCCCAGTGGCTGCTGCTGTTAAAAATGAAGTTAATGTTCCCTGCCTCAGAAACTTCGTTGAAAGCCTGTATGATACCACGCTGGAACTTTCTTCTCGAAAGAAGCATTCCTTGGCCTTGTACCCACTGGTGACCTGTCTGCTCTGCGTCAGCCAGAAACAGCTATTCCTGAACAGGTGGCAC[G/A]TTTTCCTCAACAACTGCTTATCCAACCTTAAAAATAAAGACCCCAAGATGGCTCGAGTTGCACTGGAATCTCTGTACAGATTACTCTGGGTTTACATGATCCGAATTAAATGTGAAAGCAACACGGCTACTCAGAGCCGTCTTATAACCATCATCACAACACTTTTCCCCAAAGGGTCCCGTGGTGTGGTGCCAAGGGATATGCCTCTCAACATCTTTGTGAAAATCATCCAGTTCATCGCCCAGGAACGTTTAGATTTTGCAATGAAAGAAATCATTTTTGATTTCCTTTGTGTGGGAAAACCAGCAAAAGCTTTCAGTCTCAATCCAGAGAGAATGAATATTGGCTTAAGGGCGTTCTTGGTCATAGCCGACAGCTTGCAGCAGAAAGATGGAGAACCTCCCATGCCGGTTACCGGGGCTGTTCTCCCTTCAGGAAACACACTGAGGGTGAAGAAAAC[G/A]TATCTGAGTAAGACACTAACTGAAGAGGAAGCCAAAATGATAGGCATGTCATTATATTACTCTCAAGTACGAAAAGCTGTGGACAACATTTTAAGGCACCTTGATAAAGAAGTAGGAAG[A/G]TGTATGATGCTGACTAACGTCCAGATGTTAAACAAAGAACCCGAAGACATGATCACGGGTGAGAGAAAGCCAAAAATAGATCTTTTCAGGACCTGTGTTGCTGCTATTCCTCGACTGCTTCCTGATGGGATGTCAAAACTTGAACTTATTGACCTGCTGGCGAGGCTCTCTATTCATATGGATGATGAACTGCGACATATTGCCCAAAATTCTCTTCAGGGTTTACTTGTCGACTTCTCAGACTGGAGGGAAGATGTCCTGTTTGG[C/T]TTCACCAACTTCCTGCTTCGGGAAGTAAATGACATGCATCACACGCTCCTCGACTCGTCTCTCAAGCTCCTGCTCCAGCTGCTCACCCAGTGGAAACTGGTCATACAAACGCAAGGCAAAGTCTACGAACAGGCCAACAAAATCAGAAATGCAGAG[C/G]TAATTGCCAATGGCTCCAGCCACCGAATCCAGTCGGAGCGAGGTCCCCACTGCAGTGTACTCCATGCTGTGGAAGGTTTTGCCCTGGTTTTACTCTGCAGTTTCCAGGTGGCCACA[G/C]GCAAACTGTCTGTTTTAATACTCAAGGAAATTCGAGCTTTATTTGTTGCTCTGGGTCAGCCTGAGGACGATGACAGACCTATGATTGATGTCATGGATCAGCTGAGTTCTTCCATCCTCGAAAGTTTTATTCACGTAGCAGTTTCGGATTCAGCGACCCTGCCCTTGACCCACAGTGTGGACCTGCAGTGGCTGGTGGAGTGGAGTGCTGTCCTGGTCAACAGCCATTACGACGTGAAGAGCCCTTCCCACGTCTGGATTTTCGCCCAGTCTGTCAAGGATCCCTGGGTGCTCTGCCTCTTCAGCTTCCTCCGGCAAGAGAACCTGCCCAAGCACTGCCCCACTGCCCTCAGCTATGCCTGGCCCTACGCCTTCACGCGGCTGCAGTCTGTGATGCCCCTGGTGGACCCGAATAGCCCAATTAATGCCAAGAAAACCAGCACGGCCGGCAG[C/T]GGAGACAACTACGTTACGTTGTGGAGAAATTACCTTATTCTTTGCTTTGGAGTTGCAAAACCCAGTATTATGAGCCCAGGACACTTACGAGCTTCCACTCCAGAAATAATGGCCACGACACCTGATGGTACAGTGAGCTACGATAACAAGGCCATAGGCACCCCGTCGGTGGGCGTTCTGTTAAAGCAGTTGGTGCCTTTGATGAGACTTGAGGGCATTGAGATCACAGAGTCCTTAGTTTTAGGATTTGGAAGGACAAATTCCCTTGTCTTCAGAGAATTGGTAGAAGAACTTCATCC[A/G]TTAATGAAAGAGGCCCTGGAACGAAGGCCAGAGAACAAAAAACGCCGAGAACGGCGAGACTTGTTAAGGCTACAGCTGCTTCGAATTTTTGAACTTCTGGCTGATGCTGGGGTCATAAGTGACAGCACCAATGGAGCCTTAGAACGGGACACTTTAGCCCTGGGAGCTTTGTTCTTAGAATACGTGGACCTGACCCGCATGCTCTTAGAAGCCGAAAATGATAAAGAAGTGGAAATTCTTAAAGATATCCGGGCACATTTTAGTGCCATGGTTGCCAACTTGATTCAGCGTGTTCCAGTTCACCACCGAAGATTTCTCTTCCCCCAGCAAAGCCTGAGGCATCATCTTTTCATCTTATTTAGCCAGTGGGCAGGACCCTTCAGCATTATGTTCACTCCCTTGGATCGTTACAGTGACAGAAATCATCAGATTACAAGATACCAGTATTGTGCGTTAAAGGCAATGTCAGCAGTGTTGTGCTGTGGCCCGGTGTTTGACAACGTGGGCCTTTCCCCAGATGGCTACCTATACAAGTGGCTTGACAACATTCTGGCTTGTCAAGATTTACGAGTTCATCAGCTTGGCTGCGAGGTTGTCGTCTTGCTACTGGAACTAAATCCTGACCAAATAAATCTTTTCAACTGGGCGATTGACCGGTGCTACACTGGTTCCTACCAACTTGCGTCCGGTTGCTTCAAGGCCATAGCTACTGTGTGTGGAAGCAGGAACTATCCCTTTGACATAGTGACGCTGTTAAACCTTGTTCTGTTCAAGGCCTCTGACACCAACAGAGAGATTTATGAAATCTCCATGCAGCTCATGCAGATCCTTGAAGCAAAGCTTTTTGTGTACTCGAAGAAAGTAGCCGAACAAAGACCTGGCAGTATCCTGTATGGGACGCACGGCCCACTGCCACCCCTCTACAGCGTGTCGCTGGCCTTCTTGTCGTGCGAGCTGGCCAGGATGTACCCCGAGCTCACTCTTCCTCTCTTCTCAGAAGTCAGCCAGCGCTTCCCCACAACGCATCCCAACGGGCGTCAGATCATGCTCACCTACCTGCTGCCCTGGCTGCACAACATCGAGCTGGTGGACAGCAGGCT[G/A]CTCCTCCCGGGGTCCAGCCCCAGCAGCCCCGAGGACGATGCCAAGGACCGTGAGGGAGACATGACGGCTTCTCAGGGGCTGAAAGGCAGTGGCTGGGGCTCCCCGGAGGCGACGTCCCTGGTGCTGAACAACCTCATGTACATGACGGCCAAGTATGGGGATGAAGTTCCTGGACCGGAGATGGAAAATGCCTGGAATGCATTAGCTAACAACGAGAAATGGAGCAACAACCTGCGGATCACCTTGCAGTTCCTGATCAGCCTGTGCGGGGTCAGCAGCGACACGCTCCTCCTGCCCTATATCAAGAAGGTGGC[C/T]GTCTACCTGTGCCGGAACAACACCATTCAGACCATGGAGGAGCTGCT[G/C]TT[C/T]GAGCTGCAGCAGACGGAGCCGGTGAACCCCATTGTCCAGCACTGCGACAACCCGCCCTTTTACCGCTTCACAGCCAGCAGCAAGGCCTCGGCAGCGGCCTCCGGAACCACCTCCAGCAGCAATACAGTGGTTGCCGGCCAGGACAGTTTTCCAGACGCTGAGGAGAGCAAGATACTGAAAGAATCTGATGAGAGGTTTAGTAACGTCATCAGAGCCCATACGCGTCTGGAGTCAAGATACAGCAACAGCTCAGGAGGCTCATACGATGAAGATAAAAATGACCCAATTTCTCCCTACACCGGCTGGCTTCTGACTATCACAGAGACCAAACAGCCGCAGCCCCTACCCATGCCTTGCACGGGAGGGTGCTGGGCCCCCCTGGTGGACTACCTCCCAGAGACCATCACCCCCCGGGGGCCGCTCCACAGGTGCAATGTTGCTGTCATCTTTATGACTGAGATGGTGGTGGACCACAGTGTCAGAGAGGACTGGGCTCTCCATCTCCCGCTGCTGCTCCATGCTGTCTTCCTAGGTGGCTTCGACTTCCTGAGAGAGGATCAGTCATCCCCGGTGCCAGACTCAGGGCTCAGTTCCAGCTCCACTTCATCCAGCATCAGTCTGGGAGGCAGTAGTGGGAACCTCCCACAGATGACCCAGGAGGGAGAAGATGCAGACGCGGCTGCCGACTCAGATGAGAAGGCAAACAAGCTCATTGAGTTTCTGACGACCAGGGCATTTGGTCCACTTTGGTGCCATGAGGACATCACACCCAAAAATCAGAATTCAAAGAGTGCTGAACAACTCACCAATTTTCTACGTCATGTGGTATCCGTGTTCAGAGATTCCAAATCAGGTTTCCACCTGGAGCAGCACCTGAGTGAAGTGGCGCTGCAGACGGCCCTCGCCAGCTCTTCCCGGCACTATGCCGGCCGGTCCTTCCAGATATTCCGGGCCCTCAAACAACCTCTGTCAGCACACGCCTTGTCGGA[C/T]CTCCTCTCCAGGCTGGTGGAGGTGAT[T/C]GGCGAGCACGGAGATGAGATTCAGGGCTATGTCATGGAAGCCCTTCTTACCCTGGAAGCTGCCGTGGACAACTTGTCTGACTGCTTGAAGAA[T/C]ACTGATCTCTTAACTGTACTGTCTCGCTCTTCATCACCAGACTTAAGCTCTAACACTAAACT[A/G]ACAGCTAGCAGAAAGAGCACAGGACAGCTCAATGTGAACCCTGGGGCAGCTAGCGGCAACACAGCAACTGCAGAACGCAGCCGGCATCAAAGGAGCTTCTCTGTGCCCAAGAAGTTCGGTGTTGTTGACCGGTCCTCAGACCCACCTCGGAGTGCCACCCTGGACAGAATTCAGGCCTGTACCCAACAAGGCCTCTCCTCAAAAACCAGAAGCTCATCCTCCTTGAAGGACAGCCTCACTGACCCATCACACATAAACCATCCAACCAACCTGTTGGCCACCATCTTTTGGGTCACGGTGGCTTTGATGGAATCCGATTTTGAGTTTGAATACCTAATGGCCCTGAGGCTGTTGAACAGACTGCTGGCTCACATGCCACTTGATAAAGCTGAGAACCGAGAAAAGCTAGAGAAACTCCAGGCGCAGCTCAAGTGGGCAGACTTTTCAGGGTTGCAGCAGCTGCTGTTGAAGGGGTTCACCTCCCTCACCACCACCGACCTTACCTTGCAGCTGTTCAGTTTGCTGACACCAGTGTCCAAAGTATCCATGGTGGACTCATCTCAAGCCATTGGGTTTCCACTGAATGTCTTATGTCTTCTGCCCCAGCTGATACAGCATTTTGAAAACCCCAACCAGTTCTGTAAGGATGTAGCTGAGAGAATCGCTCAGGTTTGTTTGGAAGAGAAGAACCCCAAACTTTCCAATCTTGCACACGTCATGACTCTTTATAAAACACACAGCTACACGAGGGACTGTGCCACGTGGGTCAATGTGGTCTGCCGGTACCTTCATGAAGCCTATGCTGACATCACCTTGAATATGGTTACCTACCTGGCAGAGCTTCTGGAGAAGGGCCTCCCCAGCATGCAGCAGCCCCTCCTGCAGGTGATCTACAGCCTGCTCAGCTACATGGACCTTTCTGTCGTTCCTGTAAAGCAGTTCAACGTGGAAGTTCTGAAGACCATTGAAAAATACGTGCAAAGCATTCACTGGAGAGAAGCTTTGAATATTTTGAAGCTGGTGGTTTCTCGATCTGCCAGCCTAGTTTTGCCTTCCTACCAACACAGTGACCTCTCAAAGATAGAAATACATCGAGTGTGGACGAGCTCATCCAAGGAATTACCTGGAAAAACCCTAGACTTCCACTTTGATATTTCAGAGACGCCTATCATCGGGAGGCGGTATGATGAACTGCAGAACTCCTCTGGGCGTGACGGGAAGCCCAGGCCCATGGCCGTCACCCGGAGTGCATCCTCCACCTCCTCGGGCTCCAACTCCAACGTCCTTGTCCCTGTCAGCTGGAAAAGGCCCCAGTACTCCCAGAAGAGGACCAAAGAGAAGTTGGTGCACGTCCTTTCTCTGTGTGGCCAGGAAGTAGGATTAAGCAAGAATCCATCAGTGATCTTCTCGTCGTGCGGGGACCTGGACCTGCTGGAGCATCAGACGAGCCTGGTGTCATCCGAGGACGGGGCCCGGGAGCAGGAGAACATGGATGACACCAACAGCGAGCAGCAGTTCCGGGTCTTCAGAGACTTCGACTTCCTGGACGTGGAGCTAGAGGACGGAGAGGGCGAGAGTATGGACAACTTCAACTGGGGAGTCCGCAGGCGTTCTTTGGACAGTCTGGACAAGTGTGACCTGCAGCTGCTGGAGGAGCGCCAGCTCTCAGGAAGCTCTCCCAGCCTCAATAAAATGAACCACGAGGATTCTGATGAGTCCTCTGAGGAGGAGGACCTCACCACCAGCCAGATCCTGGAGCACTCTGACCTCATCATGAATCTCTCCCCTTCGGAGGAGACTAACCCCATGGAATCACTCACGACCGCCTGCGATCTGACCCCTGCTGACCCTCACTCCTTTAACACCAGGATGGCTAGCTTTGAAGCTGCTTTGCCTGATATGAATAATCTTCAGATTTCTGAGGGTTCAAAGGCCGATGCCGTCCAGGAAGAGGAGGACGCCACTGTGCAGGAGGATGACCTTTCCAGCTCCATCAATGAACTCCCAGCCACTTTCGAATGCAGCGACAGCTTTAGCCTGGATATGACTGAGGCAGAAGAAAAAGGCAGTCGAGCGCTGGACCACTTCACTCTTGCCAGCTTCGGGGAAGGTGAGAGGGGAGTGTCGCCCCCTCCCTCGCCCTTCTTCTCGGCCATCCTTGCCGCCTTTCAGCCCACGGCCTGTGACGACGCCGAGGAGGCCTGGCGCAGCCACATCAACCAGCTCATGTGCGACTCAGACGGCTCCTGTGCTGTGTACACATTCCACGTGTTCTCCTCCTTGTTTAAGAATATTCAGAAAAGGTTCTGCTTCCTAACCTGTGATGCAGCCAGTTACCTTGGTGATAACCTCCGGGGCATTGGATCCAAGTTTGTCAGCTCTTCCCAGATGCTTACCTCCTGCTCCGAGTGCCCCACGCTCTTTGTGGATGCCGAGACCCTCCTTTCTTGTGGACTTCTGGACAAGCTCAAGTTCAGTGTTTTAGAACTGCAAGAATATTTGGATACCTACAACAACAGAAAAGAGGCCACTCTCTCATGGCTTGCAAATTGTAAGGCGACATTTGCAGGAGGATCAAGAGATGGAGTAATTACCTGTCAACCAGGGGACTCAGAGGAAAAGCAACTGGAACTGTGTCAGAGGTTGTATAAGCTACACTTCCAGCTGCTGCTGCTTTTTCAGTCCTACTGTAAGCTCATCGGCCAGGTGCACGAAGTCAGCTCCATGCCAGAGCTGCTAAATATGTCCAGGGAACTGAGTGACCTAAAGAAAAACCTGAAGGAGGCCACTGCAGCCATTGCAGCTGACCCTCTTTACATCGAGGGGGCGTGGTCCGAGCCAACCTTCACATCCACTGAGGCAGCCATCCAGTCCATGCTTGAGTGCTTGAAGAACAACGAACTTGGCAAGGCTTTACGGCAAATAAGGGAATGCAGAAGTCTGTGGCCCAATGACATCTTTGGAAGCAGTTCTGACGATGAGGTCCAGACACTGCTGAATATTTATTTCCGCCATCAAACTCTAGGACAGACGGGCACTTACGCATTGGTGGGGTCTAACCAGAGCCTGACTGAGATCTGCACCAAGCTGATGGAGCTGAACATGGAGATCCGGGACATGATCCGCAGGGCCCAGAGCTACCGGGTCCTCACCGCCGTCCTTCCCGACTCCAGCGTGTCCGGCACTAGCCTCTGACAGGAGCCTCCCATCCCCCATGGGCGCCACGCTGGGGGTGCTGCCGTGCTGGGGTGACGTCATTCAGTGTCTTCTCAGCCTTCAAAAGGCTTGGTCAGACTGTTCTCCCTCTTGTTGCCTGTAGGACTTTTCATAAAGAGGATGGCAGAACTTCCCACGTGTAGCAATACTCTTAAGAACCAAGGTAGCATAGGACTGCTCTGGAACAGCCTCCATCCATCATCCTGTGTGACACAAACGCATTATCCTTTTACTGAGCAAAAACTCACTACTGAAGGCGTGACTTCCATTGCATACCAAAGCCAACTACTCTGAACAATACCTTCCTTTCTAGAAACAATTTTAGATTGGCAAAAGTGCAATGTTTTCTTCACTAAAAAATATTTTATATTCTAAAACTTGTACATTCTTTTTCATTTTTCTCTGCCTTTGTTTTGTGTGTGTGTGTGTGAGGTGAGCTGAGGAAGGAGCAGGCAGAATGAAGCTGGCCATGGAAAACTGTAAGACGGTCAAAAGCTGACAGCCTGTGTATGTGAAAAGAGAACTGTAAATGGACAATTCAATGTACACTGAAATTTGAATACCATTACTGTGTCTAAAAGGAGCTGCTATGAAGTACCTTTCTTATGTTGCTAGGCTACTGTCTCGGAAAGCCCTGGATCTCT[C/T]TGCAGGGGTCTGTGATTAAGACCCTCGCACCAAAAATGGTCCAGACAGACATTTTTTTAAGGATCTTGGCTGCTTTTTATTAGAATGTTGCTTTTATGAACATATTTATACTACAGAAGGATGAGTGTTAATTTTAACCAACTTTGCCATTTTATAGAATAAAGTTAAAGACGTCCACATTATAAATTCCAAGTCTTTCCACCTGTTACGCATGCATATTTCAATATCCACGTGGATAGCTGGAAATAGGACATAAAGGTAAATATGAGTTGTCACTGTTTCTTAAGAAATGTCATTTTATCTGTAATATATATGTAAAGGGCCATTCTTAAGTTTTCCCCTTAAATTTAATGCTGTCAAGTGTTAGCTGTGTGCATGTAAACCTGTTGCACATCAGAAGCATATTCAAAGTTTATCTATGTAACTTATTCACTCTGTAAATACATTTAAAGTTTTTGTGATGTAATCTCGGTTGACATTCTGTTTAGAACTGCCTTTAAAGTAAAAAAAAAAAAAATCATACTGACCACAGGTTTTATTTCTATTTAATGAAGATTTTTGCTTTGATTTATATTTTTGCATGTGGGTTGCCATGATAACAACCCCAAGGAGTACCACACGCGTGTGTTCCATGACTGGCAGCCAGGGGGTTGTGAAGCAAGTACCATGACCCTGGGAAGCAGGAGGTCTCTGTAACCTCTTAAGATGGACACCAAAGACTGTAACCCTCAACCAGCTCTCACTATAATCAGAAGCAAGAAAC[C/T]AGAAGACAAGAGCTCTCATTATCCACAGCCTATCATTAGGTTTTCATTCTTAAAAATTTGAAAGAGAACCATTAATGATAAGGAAAAAGTAGTGAGTTCTATTCTTTCACCACAACCCATCTATTCTTCACCCAACTCAAAAACGGGATCATGGAAACACACAACAATCTAATTTCTATGGTCTTCCTATTCATACTCCTTAGATTAACCAACGTAACTTGCTTCCTAATATACAACTAAATCAGAAGTAGTAATTTTCTAGACGTATCCACAACAGAGTCTGATCCAAGATTTTAATTTTACCACAGATACAATTATTACCTGGGTTTTAATGACTTTCTGGACATCGCTGTTAACATACTATTTTGATGATTTTCAACTTACCCTAAAGGTGATTAACCATAATCTGTAGGAAAAAACTGAATCACAATCTCTCTGGGGGGAAAAAATTTTCACAAAATGCAGGTTTCTGTGGTTAACAACACTAGGCAACTCTGAGTTCTGCTGGGACCCCCACTATAAAGGACTGATTAGTCTTGACTGAATTTTCCACCATGGCCTCTTACCCAGCAAAGAACCACCATGATTGGGTTATTCTTTTAACAAGAGTGTTTTTGAGTGATATCATCTGTAGTCATGAGAAGCAGTGGGT[TTTT]TTTTTTTTTTCTGTTTTCTTATTTTTTTTAAACAGGTTAATGTGAAAGAGTTCTCCTTTTCCTGTTTCTTAACCTCCTCTCCCTTAGTGAAGCAGAAGGGCCCTCTGAGGCTGCAGACAGGCCCCAGAGGAGCCCAACAATCAGTGACTTGAGAGCTGCCCACAAGCCTCCTGGGGTATCGGGTTCTCCCTCTGTTACTGAAGCCTTAATGTTCACTTCCCCAGGGGCAGGCACAGGAGGACGAGTAGGATATCTGCCCTCTCCTTTAGATGCCTATCTCCTCGCCTCTGAGGAGGAACACCTCCTCCTACCACCACGCTTTTTAGCCCTA[A/G]AGTGTCACACACTTTATCCACTGGCAAAAAAAAAAAAGCAATTCAGGCAGCTGTACTGCAACTT

_______________________________________________________________________________

>BNC2_XM_012126366_AWASSI

ATGTCAGAAGAGGCAGAAGTGGATGTGAGAGAAAGAGAGACACAGAGAGACAGAGAGCCAAAGAGGGCAAGAGACTTGACTTTAAGAGACTCCTGTACTGACAACTCCATGCAGTTCGGAACCAGAGCGACTCCAGCTGAACCAGGGTTCATGGGAACATGGCAAAACGCTGATACTAACCTCTTATTCAGAATGTCCCAACAGGTCCCAGTGGCATGTGCTGGCAGAGTGCTGGGTGCAGACTTCTGCCCAAACCTGGAGGAGCCAGACCAGAGGCTGGAAGTCCAGGCCATCCGCTGCACACTGGTGAACTGCACATGTGAGTGTTTCCAGCCGGGGAAGATTAACCTGAGGACTTGCGATCAGTGTAAACATGGCTGGGTGGCACACGCCTTGGATAAGCTCAGCACACAGCACCTGTACCACCCCACCCAGGTGGAGATCGTGCAGTCCAATGTGGTGTTCGACATCAGCAGTCTGATGCTCTACGGCACACAGGCGGTGCCCGTGCGGCTCAAGATCCTACTGGACCGTCTCTTCAGCGTCCTGAAGCAAGAGGAGGTGCTCCACATCCTGCATGGCCTGGGCTGGACGCTGCGCGACTACGTCCGGGGGTACATCCTTCAGGATGCTGCCGGCAAGGTGCTGGACCGCTGGGCCATCATGTCTCGAGAAGAGGAAATCATCACCCTTCAGCAGTTTCTGCGGTTCGGAGAAACCAAATCCATCGTGGAGCTGATGGCGATTCAGGAGAAAGAAGGGCAGGCCGTAGCTGTACCATCTTCAAAGACAGACTCAGACATCAGAACTTTCATTGAGAGCAATAATCGCACCAGGAGTCCTAGCCTCCTCGCCCATCTAGAGAACAGCAACCCTTCCAGCATTCATCACTTTGAAAACATCCCCAACAGCCTTGCCTTCCTACTGCCATTCCAGTACATAAACCCGGTCTCAGCCCCGCTGCTAGGATTGCCTCCAAACGGGCTGCTGTTAGAACAACCAGGACTGAGGCTGCGGGAGCCCAGCCTTTCGACCCAGAATGAATATAATGAGAGCAGTGAGTCTGAAGTCTCCCCCACGCCCTATAAGAACGATCAAACGCCCAATAGAAATGCCCTGACCAGCATCACTAACGTGGAGCCCAAAACCGAGCCAGCCTGCGTGTCCCCCATTCAGAATTCCGCCCCAGTTAGTGATCTGTCCAAAACTGAACACCCGAAGAGCTCATTCCGGATCCACCGGATGAGAAGGATGGGGTCAGCCTCCCGGAAGGGAAGAGTGTTCTGTAACGCGTGTGGGAAGACGTTCTATGACAAAGGTACTCTCAAAATCCATTATAACGCCGTTCACCTGAAGATCAAACATCGGTGCACCATTGAAGGCTGCAATATGGTCTTTAGCTCCCTCCGCAGCCGGAACCGCCACAGTGCGAACCCTAACCCTCGCCTTCACATGCCTATGCTAAGGAACAACCGAGACAAAGATTTAATCCGGGCCACGTCCGGGGCCGCCACCCCCGTCATAGCAAGTACAAAATCGAATCTCACACTCACGAGCCCTGGCCGGCCCCCAATGGGTTTTACCACTCCCCCACTAGACCCCGTGTTACAGAACCCTCTCCCTAGCCAGCTGGTGTTCTCTGGACTAAAGACTGTCCAACCAGTTCCTCCATTTTATAGAAGTTTACTCACTCCAGGGGAAATGGTGAGTCCTCCGACCTCCCTCCCGACCAGTCCTATCATTCCAACCAGTGGTACCATAGAGCAGCACCCCCCACCCTCCTCTGAGCCAGCAGCGCCAGTAGTGATGATGGCCACTCATGAGCCCAGTGCCGACCTGGCGCCCAAGAAGAAGCCCAGGAAGTCCAGCATGCCTGTGAAGATCGAGAAGGAAATTATCGACACCGCCGATGAGTTTGACGATGACGATGACGACCCCAACGACAGCGGGGCCGTGGTCAACGATGGGGGCCATGACAATCACTGCCACTCCCAGGAGGAGATGAGTCCAGGCATGTCGGTGAAGGACTTTTCTAAGCATAGCAGGACCCGGTGCATTTCAAGGACAGAAATAAGAAGGGCTGACAGCATGACTTCTGAGGACCAAGAACCTGAGCGGGACTATGAGAATGAGTCTGAGTCTTCGGAGCCCAAACTCGGTGAGGAATCCATGGAGGGGGATGAGCACATGCATGGTGAGGTGAGCGAGAAAGTCCTGATGAGCAGTGAGAGGCCGGACGAGAACCACAGTGAGCCCTCTCACCAGGATGTCATCAAGGTGAAGGAGGAGTTTACAGATCCCACTTATGACATGTTTTACATGAGCCAGTATGGACTGTACAACGGCGGGGGTGCCAGCATGGCAGCCCTGCACGAGAGTTTTACATCGTCTCTGAATTACGGCAGCCCTCAGAAGTTCTCCCCAGAAGGGGACCTGTGTTCTAGCCCAGACCCTAAAATCTGTTATGTGTGCAAGAAGAGTTTCAAAAGCTCCTACAGCGTGAAGCTTCACTACAGAAACGTTCACTTGAAAGAGATGCATGTCTGCACGGTGGCTGGCTGCAACGCTGCCTTCCCCTCTCGCCGAAGCAGAGACAGACACAGTGCCAACATAAATCTGCATCGTAAACTGTTGACCAAAGAACTCGATGACATGGGCCTGGACTCATCACAGCCCTCCCTTAGCAAGGACCTCCGGGATGAATTTTTGATGAAGATCTATGGTGCCCAGCACCCCCTGGGGCTTGACATCAGGGAAGACGCCTCCTCTCCCGCGGGGACGGAAGACTCCCACCTGAACGGGTATGGGAGGGGCATGGCGGAGGACTACATGGTCCTGGACCTGAGCACCACCTCCAGCCTCCAGTCCAGCAGCAGCATCCATTCCTCCAGAGAATCCGATGCCGGCAGCGATGAGGGGATTCTCCTGGACGACATTGACGGGGCGAGTGACAGCGGGGAGTCGGCGCACAAGGCTGAGGCCCCCACCCTCCCTGGCGGCCTCGGGGCTGACGTTTCAGGGTCTCTGATGTTCAACAGCTTGTCCGGGAGCACTGGTGGGATCATGTGTAACATTTGCCACAAGATGTACAGCAACAAGGGGACGCTGAGGGTGCACTACAAAACCGTGCATCTGCGAGAGATGCACAAGTGCAAAGTCCCCGGTTGCAATATGATGTTTTCCTCTGTGCGAAGCCGGAATCGGCACAGTCAGAACCCTAACCTCCACAAAAACATTCCCTTCACTGCAGTAGATTAG

>BNC2_XM_012126366_Afec ASSAF

ATGTCAGAAGAGGCAGAAGTGGATGTGAGAGAAAGAGAGACACAGAGAGACAGAGAGCCAAAGAGGGCAAGAGACTTGACTTTAAGAGACTCCTGTACTGACAACTCCATGCAGTTCGGAACCAGAGCGACTCCAGCTGAACCAGGGTTCATGGGAACATGGCAAAACGCTGATACTAACCTCTTATTCAGAATGTCCCAACAGGTCCCAGTGGCATGTGCTGGCAGAGTGCTGGGTGCAGACTTCTGCCCAAACCTGGAGGAGCCAGACCAGAGGCTGGAAGTCCAGGCCATCCGCTGCACACTGGTGAACTGCACATGTGAGTGTTTCCAGCCGGGGAAGATTAACCTGAGGACTTGCGATCAGTGTAAACATGGCTGGGTGGCACACGCCTTGGATAAGCTCAGCACACAGCACCTGTACCACCCCACCCAGGTGGAGATCGTGCAGTCCAATGTGGTGTTCGACATCAGCAGTCTGATGCTCTACGGCACACAGGCGGTGCCCGTGTGGCTCAAGATCCTACTGGACCGTCTCTTCAGCGTCCTGAAGCAAGAGGAGGTGCTCCACATCCTGCATGGCCTGGGCTGGACGCTGCGCGACTACGTCCGGGGGTACATCCTTCAGGATGCTGCCGGCAAGGTGCTGGACCGCTGGGCCATCATGTCTCGAGAAGAGGAAATCATCACCCTTCAGCAGTTTCTGCGGTTCGGAGAAACCAAATCCATCGTGGAGCTGATGGCGATTCAGGAGAAAGAAGGGCAGGCCGTAGCTGTACCATCTTCAAAGACAGACTCAGACATCAGAACTTTCATTGAGAGCAATAATCGCACCAGGAGTCCTAGCCTCCTCGCCCATCTAGAGAACAGCAACCCTTCCAGCATTCATCACTTTGAAAACATCCCCAACAGCCTTGCCTTCCTACTGCCATTCCAGTACATAAACCCGGTCTCAGCCCCGCTGCTAGGATTGCCTCCAAACGGGCTGCTGTTAGAACAACCAGGACTGAGGCTGCGGGAGCCCAGCCTTTCGACCCAGAATGAATATAATGAGAGCAGTGAGTCTGAAGTCTCCCCCACGCCCTATAAGAACGATCAAACGCCCAATAGAAATGCCCTGACCAGCATCACTAACGTGGAGCCCAAAACCGAGCCAGCCTGCGTGTCCCCCATTCAGAATTCCGCCCCAGTTAGTGATCTGTCCAAAACTGAACACCCGAAGAGCTCATTCCGGATCCACCGGATGAGAAGGATGGGGTCAGCCTCCCGGAAGGGAAGAGTGTTCTGTAACGCATGTGGGAAGACGTTCTATGACAAAGGTACTCTCAAAATCCATTATAACGCCGTTCACCTGAAGATCAAACATCGGTGCACCATTGAAGGCTGCAATATGGTCTTTAGCTCCCTCCGCAGCCGGAACCGCCACAGTGCGAACCCTAACCCTCGCCTTCACATGCCTATGCTAAGGAACAACCGAGACAAAGATTTAATCCGGGCCACGTCCGGGGCCGCCACCCCCGTCATAGCAAGTACAAAATCGAATCTCACACTCACGAGCCCTGGCCGGCCCCCAATGGGTTTTACCACTCCCCCACTAGACCCTGTGTTACAGAACCCTCTCCCTAGCCAGCTGGTGTTCTCTGGACTAAAGACTGTCCAACCAGTTCCTCCATTTTATAGAAGTTTACTCACTCCAGGGGAAATGGTGAGTCCTCCGACCTCCCTCCCGACCAGTCCTATCATTCCAACCAGTGGTACCATAGAGCAGCACCCCCCACCCTCCTCTGAGCCAGCAGCGCCAGTAGTGATGATGGCCACTCATGAGCCCAGTGCCGACCTGGCGCCCAAGAAGAAGCCCAGGAAGTCCAGCATGCCTGTGAAGATCGAGAAGGAAATTATCGACACCGCCGATGAGTTTGACGATGACGATGACGACCCCAACGACAGCGGGGCCGTGGTCAACGATGGGGGCCATGACAATCACTGCCACTCCCAGGAGGAGATGAGTCCAGGCATGTCGGTGAAGGACTTTTCTAAGCATAGCAGGACCCGGTGCATTTCAAGGACAGAAATAAGAAGGGCTGACAGCATGACTTCTGAGGACCAAGAACCTGAGCGGGACTATGAGAATGAGTCTGAGTCTTCGGAGCCCAAACTCGGTGAGGAATCCATGGAGGGGGATGAGCACATGCATGGTGAGGTGAGCGAGAAAGTCCTGATGAGCAGTGAGAGGCCGGACGAGAACCACAGTGAGCCCTCTCACCAGGATGTCATCAAGGTGAAGGAGGAGTTTACAGATCCCACTTATGACATGTTTTACATGAGCCAGTATGGACTGTACAACGGCGGGGGTGCCAGCATGGCAGCCCTGCACGAGAGTTTTACATCGTCTCTGAATTACGGCAGCCCTCAGAAGTTCTCCCCAGAAGGGGACCTGTGTTCTAGCCCAGACCCTAAAATCTGTTATGTGTGCAAGAAGAGTTTCAAAAGCTCCTACAGCGTGAAGCTTCACTACAGAAACGTTCACTTGAAAGAGATGCATGTCTGCACGGTGGCTGGCTGCAACGCTGCCTTCCCCTCTCGCCGAAGCAGAGACAGACACAGTGCCAACATAAATCTGCATCGTAAACTGTTGACCAAAGAACTCGATGACATGGGCCTGGACTCATCACAGCCCTCCCTTAGCAAGGACCTCCGGGATGAATTTTTGATGAAGATCTATGGTGCCCAGCACCCCCTGGGGCTTGACATCAGGGAAGACGCCTCCTCTCCCGCGGGGACGGAAGACTCCCACCTGAACGGGTATGGGAGGGGCATGGCGGAGGACTACATGGTCCTGGACCTGAGCACCACCTCCAGCCTCCAGTCCAGCAGCAGCATCCATTCCTCCAGAGAATCCGATGCCGGCAGCGATGAGGGGATTCTCCTGGACGACATTGACGGGGCGAGTGACAGCGGGGAGTCGGCGCACAAGGCTGAGGCCCCCACCCTCCCTGGCGGCCTCGGGGCTGACGTTTCAGGGTCTCTGATGTTCAACAGCTTGTCCGGGAGCACTGGTGGGATCATGTGTAACATTTGCCACAAGATGTACAGCAACAAGGGGACGCTGAGGGTGCACTACAAAACCGTGCATCTGCGAGAGATGCACAAGTGCAAAGTCCCCGGTTGCAATATGATGTTTTCCTCTGTGCGAAGCCGGAATCGGCACAGTCAGAACCCTAACCTCCACAAAAACATTCCCTTCACTGCAGTAGATTAG

_______________________________________________________________________________________

>TYRP1_ NM_001130023_AWASSI

GCATTCTTGCTTCAAGCAGAATGAAATCTCCTACACTCCTCTCTCTGGGTTACATGTTCCTGGTCCTGCTTTTCTTCCAGCAGGCCTGGGCTCAGTTCCCGAGAGAGTGTGCTACCATTGAGGCTTTGAGGAATGGCGTGTGTTGCCCAGACCTGTCCCCACTGTCTGGGCCTGGGTCTGACCGCTGCGGTTTCTCATCAGGGAGGGGTAGATGTGAGGTGGCGATAGCCGACTCCCGGCCCCACAGCCACCACTACCCACATGATGGCCGAGACGACCGAGAGGCCTGGCCCACACGCTTCTTCAACAGGACATGCCGCTGCAGTGGCAATTTCTCAGGACACAACTGTGGAACCTGCCGCCCTGGCTGGGGAGGGGCTGCCTGTGACCAGAGGGTTCTCACAGTCAGGAGAAACCTTCTGGACTTAAGCACAGAAGAAAAGAACCGCTTTGTCCAGGCTCTGGATATGGCAAAGCACACCACTCACCCTCAGTTTGTCATCGCCACCAGGAGGTCAGAAGAAATATTGGGGCCAGATGGCAACACACCACAGTTTGAGAACATCTCCATTTATAACTACTTTGTTTGGACACACTACTACTCAGTCAAAAAGACTTTCCTGGGAGCAGGACAGGAAAGCTTTGGTGAAGTGGATTTCTCTCACGAGGGACCAGCATTTCTCACATGGCACAGGTACCACCTGCTGCAGCTGGAGAGAGACATCCAGGAAATGTTGCAGGATCCTTCTTTCTCCCTCCCTTACTGGAATTTTGCCACTGGGAAGAACACCTGCGACATTTGCACCGATGACTTGATGGGATCAAGAAGCAACTTTGATTCCACTCTTATAAGCCCGAACTCTGTCTTTTCTCAATGGCGAGTAGTCTGCGAATCCTTGGAAGATTATGATACCCTGGGAACCCTATGCAACAGCACTGAGGGTGGGCCAATTAAGAGAAACCCAGCTGGAAATGTGGCCAGACCAATGGTGCAACGTCTTCCTGAACCACAGGATATCGCTCAGTGCTTAGAAGTTGGTTCATTTGACACACCTCCTTTTTATTCTAATTCTACAAACAGCTTCCGAAACACAGTGGAAGGTTACAGTGATCCCACAGGAAGGTATGACCCTGCTGTGCGAAGCCTTCACAATTTGGCTCATCTATTCCTGAATGGAACAGGGGGACAAACTCATTTATCTCCCAATGATCCTATTTTTGTCCTCCTACATACATTCACTGACGCGGTATTTGATGAATGGCTGAGGCGATACAATGCTGATATATCCACATTTCCACTGGAAAATGCCCCTATTGGACATAATAGACAATACAACATGGTACCATTTTGGCCTCCAGTTACCAACATAGAAATGTTTGTTACTGCTCCAGACAACCTGGGCTATACTTACGAAGTTCAATGGCCAGGTCGGAGTTTTAGTATTCCTGAGATTGTCACCATAGCAGTAGTGGCTGCGTTATTACTGGTCGCGGTCATTTTTGCGGGCGCCTCTTGTCTGATTCGTGCCAGAAGCAACATGGATGAAGCAAATCAGCCTCTCCTTACTGATCAGTATCAACACTACATTGAAGAAAATGAAAAAATCCATAATCCTAATCAGTCTATGGTCTAATGACAAGTGTAAATTCTCATATGCAT

>TYRP1_ NM_001130023_Afec-ASSAF

GCATTCTTGCTTCAAGCAGAATGAAATCTCCTACACTCCTCTCTCTGGGTTACATGTTCCTGGTCCTGCTTTTCTTCCAGCAGGCCTGGGCTCAGTTCCCGAGAGAGTGTGCTACCATTGAGGCTTTGAGGAATGGCGTGTGTTGCCCAGACCTGTCCCCACTGTCTGGGCCTGGGTCTGACCGCTGCGGTTTCTCATCAGGGAGGGGTAGATGTGAGGTGGCGATAGCCGACTCCCGGCCCCACAGCCACCACTACCCACATGATGGCCGAGACGACCGAGAGGCCTGGCCCACACGCTTCTTCAACAGGACATGCCGCTGCAGTGGCAATTTCTCAGGACACAACTGTGGAACCTGCCGCCCTGGCTGGGGAGGGGCTGCCTGTGACCAGAGGGTTCTCACAGTCAGGAGAAACCTTCTGGACTTAAGCACAGAAGAAAAGAACCGCTTTGTCCAGGCTCTGGATATGGCAAAGCACACCACTCACCCTCAGTTTGTCATCGCCACCAGGAGGTCAGAAGAAATATTGGGGCCAGATGGCAACACACCACAGTTTGAGAACATCTCCATTTATAACTACTTTGTTTGGACACACTACTACTCAGTCAAAAAGACTTTCCTGGGAGCAGGACAGGAAAGCTTTGGTGAAGTGGATTTCTCTCACGAGGGACCAGCATTTCTCACATGGCACAGGTACCACCTGCTGCAGCTGGAGAGAGACATCCAGGAAATGTTGCAGGATCCTTCTTTCTCCCTCCCTTACTGGAATTTTGCCACTGGGAAGAACACCTGCGACATTTGCACCGATGACTTGATGGGATCAAGAAGCAACTTTGATTCCACTCTTATAAGCCCGAACTCTGTCTTTTCTCAATGGCGAGTAGTCTGCGAATCCTTGGAAGATTATGATACCCTGGGAACCCTATGCAACAGCACTGAGGGTGGGCCAATTAAGAGAAACCCAGCTGGAAATGTGGCCAGACCAATGGTGCAACGTCTTCCTGAACCACAGGATATCGCTCAGTGCTTAGAAGTTGGTTCATTTGACACACCTCC[T/C]TTTTATTCTAATTCTACAAACAGCTTCCGAAACACAGTGGAAGGTTACAGTGATCCCACAGGAAGGTACGACCCTGCTGTGCGAAGCCTTCACAATTTGGCTCATCTATTCCTGAATGGAACAGGGGGACAAACTCATTTATCTCCCAATGATCCTATTTTTGTCCTCCTACATACATTCACTGACGCGGTATTTGATGAATGGCTGAGGCGATACAATGCTGATATATCCACATTTCCACTGGAAAATGCCCCTATTGGACATAATAGACAATACAATATGGTACCATTTTGGCCTCCAATTACCAACATAGAAATGTTTGTTACTGCTCCAGACAACCTGGGCTATACTTACGAAGTTCAATGGCCAGGTCGGAGTTTTAGTATTCCTGAGATTGTCACCATAGCAGTAGTGGCTGCGTTATTACTGGTAGCGGTCATTTTTGCGGGCGCCTCTTGTCTGATTCGTGCCAGAAGCAACATGGATGAAGCAAATCAGCCTCTCCTTACTGATCAGTATCAACACTACATTGAAGAAAATGAAAAAATCCATAATCCTAATCAGTCTATGGTCTAATGACAAGTGTAAATTCTCATATGCAT

_______________________________________________________________________________________

>CALCRL_XM_012131514_AWASSI

AATAAAAAAGGAGTGCTGCTTTCCTGGGGTATGCCATTTGAGAAAGAAAGTAACCTAACACTAACTGCAGGCGGGGTCAGAGTGATGTAACAGGAAGCTCTGATGTTTCCCTTCTGCTGCTGAGCACATACAATGTGACAACCCTTCCAGTCCACTCAGAACAACACTCTCTCCAGCAGTGTCACCTCCTGCCTGAGGATCACCAAGCTCTGCTAACTGGACCTCACCCTGCCTGCAGGATCATATTGCAAGGCTTTCACCCTTCCCCACCTTGCCTGGGGGTACATTTCTTCTGCGGAATCTCAGAAAATCAAATTCCATCCTAAGAATAGTTCACCAAGAATTTCCTTAGGAGCTGTTCTGGGGTCCTACGATGATACACCAACCACTTGTGCTATCACCAGTCAAAACTATGGATGGCCCTACCTTCAGGGGATCTTCCTGACCCAGGGCTTGAAATCACATCTCTTGCACTGCAAGCGGAATCTTTACCATTGGAGGCACCAGGAAAGCGCAATAGAAGTAGAGGTGGAAATATTTAGTACTTCGATCACAGCTTGGAATTGAGTCATGACCTCCAGATTTAAGACATTCTTCAAGACAATTTTGAATATGATCCAAGAGAAAATGTGATTTGAGTCTGGAGACAATTGTGAATTCAGTGTAGCTGCCTGAAAAGAAAACATTATTTGGAAGACTGCTACAATAAAAAGAGAAGTTTCTTTTAGTTTGATTATATATATAGCATATTTCATTTTGGCTTTAATGATGGAGAAAAAGTTTTTCCTGTCTTTTCTGTTCCTCTTGCCTTTTTTCATGATTCTTGTTATAGCAGAATC[T/C]GAAGAAGAGAATCCCGATGACTTAATTCAGCTGGGTGTTACTAGAAATAAAATCATGACAGCTCAATATGAATGTTACCAGAAAATTATGCAAGACCCTGTTCAACAAACAGAAGGCATTTATTGTAACAGAACCTGGGATGGATGGCTGTGCTGGAATGATGTTGCTGCAGGAACGGAATCAATGCAGCACTGCCCTGATTACTTTCAGGATTTCGATCCTTCAGAAAAAGTTACAAAAATCTGTGACCAAGATGGAAACTGGTTTAGACATCCAGCAAGCAACAGAACATGGACAAATTATACCCAGTGTAATGTTAACACGCATGAGAAAGTGAAGACTGCACTGAATTTGTTTTACCTGACTATAATTGGACATGTATTATCGATTGCATCACTGCTTATCTCACTTGGCATATTCTTTTATTTCAAGAGCCTAAGTTGCCAAAGGATTACCCTGCACAAAAATTTGTTCTTCTCTTTTGTTTGTAATTCT[A/G]TCATAACCATCATTCATCTCACTGCAGTGGCCAACAACCAGGCCTTAGTGGCCACAAATCCTGTTAGTTGTAAAGTGTCCCAGTTCATTCATCTTTACCTGATGGGCTGTAACTACTTTTGGATGCTTTGTGAAGGCATTTACCTACATACGCTTGTTGTAGTGGCTGTATTTGCAGAGAAGCAGCACTTGATGTGGTATTATTTTCTTGGCTGGGGATTTCCACTGATTCCTGCTTGTATTCACGCTGTTGCCAGAAGATTATATTACAATGACAACTGCTGGATCAGTTCTGATACGCAACTTCTCTACATTATTCATGGCCCAATTTGTGCTGCTTTATTGGTGAATCTTTTTTTCCTATTAAATATTGTACGTGTTCTTATCACCAAGTTAAAAGTTACTCACCAAGCAGAATCCAATCTCTACATGAAAGCTGTGAGAGCTACGCTTATCCTGGTGCCATTACTTGGCATTGAATTTGTGCTGATTCCATGGCGACCTGAAGGGAAGATTGCAGAAGAGATATATGATTACATCATAAACATCCTCATGCACTATCAGGGTCTCTTGGTATCTACAATTTTCTGCTTCTTTAATGGAGAGGTTCAAGCAATTCTGAGAAGAAACTGGAATCAATATAAAATCCAATTTGGAAACAACTTTTCCCACTCAGATACTCTCCGTAGTGCATCTTACACAGTTTCAACAAT[C/T]AGTGACGGTACAGGTTACAGTCATGACTGTCTAAGCGAACACTTAAATGGAAAAAGCATCCACGATACTGAAAACGTGGTCATAAAACCGGAAAAGTTATACGATTGATAATAGAGGGTGTATTGCTGAACTATTTTTTGCCACTCCTAACTCAAGGACTTGGATCCATGACTTTACAACCACAAGACTTGAGTATTAGATGAATTATTTGAAGGTGACAAAGAAAACCCTCATGAATTCAGTAGTCTGTTGATAAATGTTTCACACTAGCTCCATGGGGGAAAATTGTCTTCATCTATGATTTTTGCCAGTTTTTATGTTGTAAATACTCCCACCATGACTTATTTCCAGCTATCAACTTAACATCACTGCATGTGGAACTGGGTAAAGATGAGCACATTCTATTCCTGTAGGTTGGTTTGCGCCACCTCCAGGACACTACTGCATGAATCCACAAATAGTTATAAAAGTTCATAGAAACGGTGGGCACAGTTCCACTCCTGTTGCCTAAAGAGGCCTAGTGAAGGCTTATAGATTTAGAGGGAAAATGACTTTTCATTTATTTTTAAAATCTTTTTCCCAAGTTCATTGCTGCAGTTGATTTTTTACCCAGAAATTGTTCTAGCTCTTTTGTTTTTATTTTTTAAAATGGACCTGATAATTAATTATTCCTCTTTGCTTACTGTCTTCTCCCTAGAAAAAATATTGAGCAGAATTGCTGTTGGCTACGTATTTGCTGATTCATCACAGTTATACCCTTTGTCATATGCTTTTTTGAGACTTGATGAATAATATATATGACATGCAATCCTGCTTTGGTATCATTAGGGAGACATCTTGGTTGATACTACAAAACATGTTGTCAACAACTTTCCCATCTTACTACACAAATGGGAAGGAATTCATGTCCATGTACCTGTAAATAGAAACCTTGCCCCTTCCATTTCTACTATATGGACAAATTAGCAATTATTTTACATGAAGGAAATCAGTGAAGGGTTTATTATTTCCTCAAAAGTTTGTACAAATTGTGAAAAATGAGCTTGTAAATACTCTATCATTCTGTTTTATAGCCTCAAATATATACAATCTACCTAATTTTTAAAACAAATGCTTAATGTAACAAAGTATGCATGCTAATATCTGATACTGTCTCTGGGCTAATTTTATAATAAAATAGAATCTGGAATTCTATACTTGTTAAATATTTTAAAGACAACCAGATGTCAGCATCAGAAGTTTGAGAACTAGGTTTGAGAAATATCTATGATAAGATATAAAATATTTATTTAAAAATCCCTCAAGGCCATTGTTTTATTGAATATATTTGCTTTGGTGATTCAAATCTTAATAATAGATATGTTTGACATATTTTTCCTTTTATTTTGACGATGAATTTTCATTCTAATCCAGAAAATTTAAATGCCTTCTGTAACAAATGCCTGTTTGTTACTCTTTGGGTTTTTATCCCATTAAAAATGTTACTTTTCTGACTTACTATAAGATAGTTTTGGAAATACTCCAGTCTGTTTTTTAAAAGTAGACAAATATATATCATATAAAATGTTTTAATATTTTAATAGAGCTAGTATATATGATGATATAATACAATGAAAGACCTGAATGTAACAGTGGGTAAGAATTTTTGTTGGAACATTTATGGAATAATGAAATTAAGATGAAAGAAATCTTCCCTAATCAATTAAAGTGATATTTAAATGGGATTCGTTTTCCCTAATGTTATTTTCCACTGAAATTTCTGAAATTCATTAAGTCAATTTGGGCAGGAGAATTCATTATTTTTCTTTTTTGTGTGTGGTGTGTCAATCTGCTTAAAATAGATTAGACTAATGACATAGTCATCACAGGTTTTTTCTTAAGCTGTAAATAAACAGTAAGTTCAGAAGGCCTCTGAAATTGTCCAGGAAAATCATCGAAATTAATAAGAATTAACTTATTAAAAGGCCTGATCAATGGATTTATGATTTATCTATGCACATGTAAAAGCTGTGTTGTTAGTCTCAGATTTATGGATTTCAAAATAAGTTGTCATAAAAAGGTATGCAGGCTGTTCTGGACTTTCTAATGCTAAATAATATAATTCCTTTAAATAAACTTA[T/C]GTTTAGCTATTTATTATTCAAAGACACAGATTCAAGTCCTTTGCTGGCACTGTTCATATGTTTCAGCATTCTACCAGACAAACTATCAAGTCTCAACCATAAATGACAATAGAAAATGTCAAATTATCAGCAAGCGTCATATTAAAAATACATGGTAACTTTCAAGTATTTTTACTGTGCTTTTATCATATTCGGTTTAGATCAGATTTCCTTTTTGGCCAGGATTTATCTATTGTACCACCTTTAGGCGTAGACAGTCCACGATTAATCAGTGTTAGTTTTGTACCCATATATTTTTGGAAGTAATATTTTTAAGTATTAGACTTCTCTAAAGCTGAAGTACTAATAATTAAGATCTCCTTTGT[A/G]TTTTACTTATGAAGAAAAAGTAAAATATATAATTTTTAAAAACAAACTGCTTTGGTATCTAAAAGCTTACTGGGGAATTTCTAACTTTTAAAATGTAAATCTGCTACTTTTCTGCAAGTACTTGCAAAAGTCATGTTAATTTTAAAAAATTCTAGTCTGTCATTTTTTTTAAATTCTAAATTTATTTAGCGTGTCTATCCATAGAATATGCATTCTGGCATTTAGTGTGTGTTCTAATGTAATCCAATGTACATAGAAGTAGTGGTAGCTGTGGTGCTGTATTTATTGCTTGATAAC[T/A]GTTTTACAATGTGAACTTATTTTAATTTTCACTGGTTTTAATATGCTAATAAAAACTATCATTTCTTTTATTTCTAAAACATACCCAAGATATTCGGTTCAATTAACTCATGTTATGCCTACTTAATGTATATCTCTATATTTTGATTGTATGAAAATATTAAAGTTATGAGCTAAAGTTGATTTTCACTCATGCTTACTGGAGTGCTGA[G/A]TAATCTAAATTATAATGATTTTTAATTCTTTACAGCAACCAGTGAAACACTTAAAATTGATGAGTATATCTCAGTCATCCTCTTTGAAGTCTTCCATAATCCTATAAATCATTCTATAAAGCAATTTGTTTTGTAAGCTAGAAAATTCTAAATAAACTACAATGTATGCAAATTCCTTCTTAGACTGAAAGAAAGGAAAAGATGCCCAGGGATAAGAAAATGCAGAGATTTGTCTCCTTTCTCCACTATGCTCCATTGGAATAGTTTTGTATTGAATAATAGAAAGGAAAAGAACTCTTTAAGTATACCGTTCACTCATTTCAAGTTGTTCTTAGTGTATTATATTAGTGTTGTC[A/G]CCACAGTCAGTTTTAGTACATTTTCATGACCTCAGGAGTTAACCACCTGTCCTCGCCATCACTTCTCTCATCCTGTCCCTGCTGTCACCTGAGCCCCAAGAACACGAACCTACTTTTTTTCTCAGTAGAC[T/G]TCCCTATTCTGACTTTCATATGAATGGAATCATATGGAATATGGTGTTTTGTGACTGACTTTTTTCACCTGTAATATTTTCAGGGTTCATCATGTTTTAAATTGTCTCATCATTACATTCCTTTCTTTGCCTGAATTATTAAACTGTATGGATTTACCAAA

>CALCRL_XM_012131514_Afec ASSAF

AATAAAAAAGGAGTGCTGCTTTCCTGGGGTATGCCATTTGAGAAAGAAAGTAACCTAACACTAACTGCAGGCGGGGTCAGAGTGATGTAACAGGAAGCTCTGATGTTTCCCTTCTGCTGCTGAGCACATACAATGTGACAACCCTTCCAGTCCACTCAGAACAACACTCTCTCCAGCAGTGTCACCTCCTGCCTGAGGATCACCAAGCTCTGCTAACTGGACCTCACCCTGCCTGCAGGATCATATTGCAAGGCTTTCACCCTTCCCCACCTTGCCTGGGGGTACATTTCTTCTGCGGAATCTCAGAAAATCAAATTCCATCCTAAGAATAGTTCACCAAGAATTTCCTTAGGAGCTGTTCTGGGGTCCTACGATGATACACCAACCACTTGTGCTATCACCAGTCAAAACTATGGATGGCCCTACCTTCAGGGGATCTTCCTGACCCAGGGCTTGAAATCACATCTCTTGCACTGCAAGCGGAATCTTTACCATTGGAGGCACCAGGAAAGCGCAATAGAAGTAGAGGTGGAAATATTTAGTACTTCGATCACAGCTTGGAATTGAGTCATGACCTCCAGATTTAAGACATTCTTCAAGACAATTTTGAATATGATCCAAGAGAAAATGTGATTTGAGTCTGGAGACAATTGTGAATTCAGTGTAGCTGCCTGAAAAGAAAACATTATTTGGAAGACTGCTACAATAAAAAGAGAAGTTTCTTTTAGTTTGATTATATATATAGCATATTTCATTTTGGCTTTAATGATGGAGAAAAAGTTTTTCCTGTCTTTTCTGTTCCTCTTGCCTTTTTTCATGATTCTTGTTATAGCAGAATCCGAAGAAGAGAATCCCGATGACTTAATTCAGCTGGGTGTTACTAGAAATAAAATCATGACAGCTCAATATGAATGTTACCAGAAAATTATGCAAGACCCTGTTCAACAAACAGAAGGCATTTATTGTAACAGAACCTGGGATGGATGGCTGTGCTGGAATGATGTTGCTGCAGGAACGGAATCAATGCAGCACTGCCCTGATTACTTTCAGGATTTCGATCCTTCAGAAAAAGTTACAAAAATCTGTGACCAAGATGGAAACTGGTTTAGACATCCAGCAAGCAACAGAACATGGACAAATTATACCCAGTGTAATGTTAACACGCATGAGAAAGTGAAGACTGCACTGAATTTGTTTTACCTGACTATAATTGGACATGTATTATCGATTGCATCACTGCTTATCTCACTTGGCATATTCTTTTATTTCAAGAGCCTAAGTTGCCAAAGGATTACCCTGCACAAAAATTTGTTCTTCTCTTTTGTTTGTAATTCT[A/G]TCATAACCATCATTCATCTCACTGCAGTGGCCAACAACCAGGCCTTAGTGGCCACAAATCCTGTTAGTTGTAAAGTGTCCCAGTTCATTCATCTTTACCTGATGGGCTGTAACTACTTTTGGATGCTTTGTGAAGGCATTTACCTACATACGCTTGTTGTAGTGGCTGTATTTGCAGAGAAGCAGCACTTGATGTGGTATTATTTTCTTGGCTGGGGATTTCCACTGATTCCTGCTTGTATTCACGCTGTTGCCAGAAGATTATATTACAATGACAACTGCTGGATCAGTTCTGATACGCAACTTCTCTACATTATTCATGGCCCAATTTGTGCTGCTTTATTGGTGAATCTTTTTTTCCTATTAAATATTGTACGTGTTCTTATCACCAAGTTAAAAGTTACTCACCAAGCAGAATCCAATCTCTACATGAAAGCTGTGAGAGCTACGCTTATCCTGGTGCCATTACTTGGCATTGAATTTGTGCTGATTCCATGGCGACCTGAAGGGAAGATTGCAGAAGAGATATATGATTACATCATAAACATCCTCATGCACTATCAGGGTCTCTTGGTATCTACAATTTTCTGCTTCTTTAATGGAGAGGTTCAAGCAATTCTGAGAAGAAACTGGAATCAATATAAAATCCAATTTGGAAACAACTTTTCCCACTCAGATACTCTCCGTAGTGCATCTTACACAGTTTCAACAATTAGTGACGGTACAGGTTACAGTCATGACTGTCTAAGCGAACACTTAAATGGAAAAAGCATCCACGATACTGAAAACGTGGTCATAAAACCGGAAAAGTTATACGATTGATAATAGAGGGTGTATTGCTGAACTATTTTTTGCCACTCCTAACTCAAGGACTTGGATCCATGACTTTACAACCACAAGACTTGAGTATTAGATGAATTATTTGAAGGTGACAAAGAAAACCCTCATGAATTCAGTAGTCTGTTGATAAATGTTTCACACTAGCTCCATGGGGGAAAATTGTCTTCATCTATGATTTTTGCCAGTTTTTATGTTGTAAATACTCCCACCATGACTTATTTCCAGCTATCAACTTAACATCACTGCATGTGGAACTGGGTAAAGATGAGCACATTCTATTCCTGTAGGTTGGTTTGCGCCACCTCCAGGACACTACTGCATGAATCCACAAATAGTTATAAAAGTTCATAGAAACGGTGGGCACAGTTCCACTCCTGTTGCCTAAAGAGGCCTAGTGAAGGCTTATAGATTTAGAGGGAAAATGACTTTTCATTTATTTTTAAAATCTTTTTCCCAAGTTCATTGCTGCAGTTGATTTTTTACCCAGAAATTGTTCTAGCTCTTTTGTTTTTATTTTTT[T/A]AAA[C/T]GGACCTGATAATTAATTATTCCTCTTTGCTTACTGTCTTCTCCCTAGAAAAAATATTGAGCAGAATTGCTGTTGGCTACG[A/T]ATTTGCTGATTCATCACAGTTATACCCTTTGTCATATGCTTTTTTGAGACTTGATGAATAATATATATGACATGCAATCCTG[G/C]TTTGGTATCATTAGGGAGACATCTTGGTTGATACTACAAAACATGTTGTCAACAACTTTCCCATCTTACTACACAAATGGGAAGGAATTCATGTCCATGTACCTGTAAATAGAAACCTTGCCCCTTCCATTTCTACTATATGGA[C/A]AAATTAGCAATTATTTTACATGAAGGAAATCAGTGAAGGGTTTATTATTTCCTCAAAAGTTTGTACAAATTGTGAAAAATGAGCTTGTAAATA[G/C]TCT[G/A]TCATTCT[A/G]TTTTATAGCCTCAAATATATACAATCTACCTAATTTTTAAAACAAATGCTTAATGTAACAAAGTATGCATGCTAATATCTGATACTGTCTCTGGGCTAATTTTATAATAAAATAGAATCTGGAATTCTATACTTGTTAAATATTTTAAAGACAACCAGATGTCAGCATCAGAAGTTTGAGAACTAGGTTTGAGAAATATCTATGATAAGATATAAAATATTTATTTAAAAATCCCTCAAGGCCATTGTTTTATTGAATATATTTGCTTTGGTGATTCAAATCTTAATAATAGATATGTTTGACATATTTTTCCTTTTATTTTGACGATGAATTTTCATTCTAATCCAGAAAATTTAAATGCCTTCTGTAACAAATGCCTGTTTGTTACTCTTTGGGTTTTTATCCCATTAAAAATGTTACTTTTCTGACTTACTATAAGATAGTTTTGGAAATACTCCAGTCTGTTTTTTAAAAGTAGACAAATATATATCATATAAAATGTTTTAATATTTTAATAGAGCTAGTATATATGATGATA[C/T]AATACAATGAAAGACCTGAATGTAACAGTGGGTAAGAATTTTTGTTGGAACATTTATGGAATAATGAAATTAAGATGAAAGAAATCTTCCCTAATCAATTAAAGTGATATTTAAATGGGATTCGTTTTCCCTAATGTTATTTTCCACTGAAATTTCTGAAATTCATTAAG[C/T]CAATTTGGGCAGGAGAATTCATTATTTTTCTTTTTTGTGTGTGGTGTGTCAATCTGCTTAAAATAGATTAGACTAATGAC[A/T]TAGTCATCACAGGTTTTTTCTTAAGCTGTAAATAAACAGTAAGTTCAGAAG[C/G]CCTCTGAAATTGTCCAGGAAAATCATCGAAATTAATAAGAATTAACTTATTAAAAGGCCTGATCAATGGATTTATGATTTATCTAT[A/G]CAC[G/A]TGTAAAAGCTGTGTTGTTAGTCTCAGATTTATGGATTTCAAAATAAGTTGTCATAAAAAGGTATGCAGGCTGTTCTGGACTTTCTAATGCTAAATAATATAATTCCTTTAAATAAACTTATGTTTAGCTATTTATTATTCAAAGACACAGATTCAAGTCCTTTGCTGGCACTGTTCATATGTTTC[C/A]GCATTCTACCAGACAAACTATCAAGTCTCAACCATAAATGACAATAGAAAATGTCAAATTATCAGCAAGCGTCATATTAAAAATACATGGTAACTTTCAAGTATTTTTACTGTGCTTTTATCATATTCGGTTTAGATCAGATTTCCTTTTTGGCCAGGATTTATCTATTGTACCACCTTTAGGCGTAGACAGTCCACGATTAATCAGTGTTAGTTTTGTACCCATATATTTTTGGAAGTAATATTTTTAAGTATTAGACTTCTCTAAAGCTGAAGTACTAATAATTAAGATCTCCTTTGTATTTTACTTATGAAGAAAAAGTAAAATATATAATTTTTAAAAACAAACTGCTTTGGTATCTAAAAGCTTACTGGGGAATTTCTAACTTTTAAAATGTAAATCTGCTACTTTTCTGCAAGTACTTGCAAAAGTCATGTTAATTTTAAAAAATTCTAGTCTGTCATTTTTTTTAAATTCTAAATTTATTTAGCGTGTCTATCCATAGAATATGCATTCTGGCATTTAGTGTGTGTTCTAATGTAATCCAATGTACATAGAAGTAGTGGTAGCTGTGGTGCTGTATTTATTGCTTGATAACTGTTTTACAATGTGAACTTATTTTAATTTTCACTGGTTTTAATATGCTAATAAAAACTATCATTTCTTTTATTTC[G/T]AAAACATACCCAAGATATTCGGTTCAATTAACTCATGTTATGCCTACTTAATGTATATCTCTATATTTTGATTGTATGAAAATATTAAAG[C/T]TATGAGCTAAAGTTGATTTTCACTCATGCTTACTGGAGTGCTGA[G/A]TAATCTAAATTATAATGATTTTTAATTCTTTACAGCAACCAGTGAAACACTTAAAATTGATGAGTATATCTCAGTCATCCTCTTTGAAGTCTTCCATAATCCTATAAATCATTCTATAAAGCAATTTGTTTTGTAAGCTAGAAAATTCTAAATAAACTACAATGTATGCAAATTCCTTCTTAGACTGAAAGAAAGGAAAAGATGCCCAGGGATAAGAAAATGCAGAGATTTGTCTCCTTTCTCCACTATGCTCCATTGGAATAGTTTTGTATTGAATAATAGAAAGGAAAAGAACTCTTTAAGTATACCGTTCACTCATTTCAAGTTGTTCTTAGTGTATTATATTAGTGTTGTCACCACAGTCAGTTTTAGTACATTTTCATGACCTCAGGAGTTAACCACCTGTCCTCGCCATCACTTCT[A/C]TCATCC[C/T]GTCCCTGCTGTCACCTGAGCCCCAAGAACACGAACCTACTTTTTTTCTCAGTAGAC[T/G]TCCCTATTCTGACTTTCATATGAATGGAATCATATGGAATATGGTGTTTTGTGACTGACTTTTTTCACCTGTAATATTTTCAGGGTTCATCATGTTTTAAATTGTCTCATCATTACATTCCTTTCTTTGCCTGAATTATTAAACTGTATGGATTTACCAAA

_______________________________________________________________________________________

>GRB14_XM_004004651_AWASSI

ATGACCACTTCCCTGCAAGATGGGCAGAGCGCCGCGGGTCGGGCGGCTGCCCGGGACTCGCCGCTGGCCGCCCAGGTGTGCGGCGCTGCCCAGGGGAGGGGCGACGCCCGCGACCTGGCGCCGGGCCCCTGGCTGCACGCGCGGGCACTCCTGCCCCCTCCGGACGCGACCCGCGGCTGTGCGGCAGACAGGAGAAAAAAGAAAGATCTTGATGTTCTGGAAATGCCATCTATTCCAAATCCTTTTCCTGAGCTATGCTGCTCTCCATTTACATCTGTGTTGTCAGCAGGCCTGTTTCCCAAAGCAAATTCAAGGAAAAAACAGGTAATTAAAGTATACAGTGAAGATGAAACCAGCAGAGCTTTAGAGGTACCCAGTGACATAACTGCCCGAGATGTTTGCCAGCTGTTGATCCTGAAGAATCATTACATTGATGACCACAGCTGGACCCTTTTTGAGCACCTGCCTCATGTAGGTCTAGAAAGAACAATAGAAGACCACGAGCTGGTGATTGAAGTGCTATCTAACTGGGGAATGGAAGAAGAAAATAAGCTATACTTTAGGAAAAATTATGCCAAATATGAATTCTTTAAAAACCCAATGTATTTTTTTCCAGAGCATATGGTGTCTTTTGCAACTGAAACCAATGGTGAAATATCCCCCACACAGATTTTGCAGATGTTTCTAAGTTCAAGCACATATCCTGAAATCCATGGCTTCTTACATGCAAAAGAACAGGGAAAGAAGTCCTGGAAAAAGATTTATTTCCTTTTAAGAAGATCCGGTTTATATTTTTCTACTAAAGGGACATCAAAGGAACCACGGCATTTGCAGTTTTTCAGCGAATTTGGCAATAGT[A/G]ATATTTATGTGTCACTGGCAGGCAAAAAAAAACACGGAGCACCGACTAACTATGGATTCTGCTTTAAGCCTAACAAAGCGGGAGGGCCCCGAGACCTGAAAATGCTCTGTGCAGAAGAAGAGCAGAGTAGGACGTGCTGGGTGACCGCGATTAGATTGCTTAAGTATGGCATGCAGCTGTACCAGAATTACATGCATCCATATCAAGGCAGAAGTGGCTACAGTTCTCAGAGTATATCTCCCATGAGAAGTATATCAGAGAATTCCCTGGTAGCAATGGACTTCTCAGGCCAGAAAACAAGAGTTATAGAAAATCCCACTGAAGCCCTTTCTGTTGCAGTTGAAGAAGGACTAGCTTGGAGGAAAAAAGGATGTTTGCGCCTGGGCAGTCAC[T/G]GTAGCCCCACTGCATCCTCACAGAGCTCTGCCACAAGCATGGCTATCCACCGGTCCCAACCATGGTTTCACCACAAAATTTCTAGAGAGGAAGCTCAGCGATTGATTATTCAGCAAGGTCTAGTGGATGGAGTTTTCTTGGTACGGGATAGTCAGAGTAACCCCAAAACTTTTGTACTGTCAATGAGTCATGGACAAAAAATAAAGCACTTTCAAATTATACCAGTGGAAGATGATGGTGAGATGTTCCACACCCTAGATGATGGACATACAAGATTTACGGATCTAATCCAGTTGGTGGAGTTCTATCAACTCAATAAGGGAGTTCTTCCTTGCAAGCTGAAACATTATTGTGCTAGGATTGCACTTTAG

>GRB14_XM_004004651_Afec ASSAF

ATGACCACTTCCCTGCAAGATGGGCAGAGCGCCGCGGGTCGGGCGGCTGCCCGGGACTCGCCGCTGGCCGCCCAGGTGTGCGGCGCTGCCCAGGGGAGGGGCGACGCCCGCGACCTGGCGCCGGGCCCCTGGCTGCACGCGCGGGCACTCCTGCCCCCTCCGGACGCGACCCGCGGCTGTGCGGCAGACAGGAGAAAAAAGAAAGATCTTGATGTTCTGGAAATGCCATCTATTCCAAATCCTTTTCCTGAGCTATGCTGCTCTCCATTTACATCTGTGTTGTCAGCAGGCCTGTTTCCCAAAGCAAATTCAAGGAAAAAACAGGTAATTAAAGTATACAGTGAAGATGAAACCAGCAGAGCTTTAGAGGTACCCAGTGACATAACTGCCCGAGATGTTTGCCAGCTGTTGATCCTGAAGAATCATTACATTGATGACCACAGCTGGACCCTTTTTGAGCACCTGCCTCATGTAGGTCTAGAAAGAACAATAGAAGACCACGAGCTGGTGATTGAAGTGCTATCTAACTGGGGAATGGAAGAAGAAAATAAGCTATACTTTAGGAAAAATTATGCCAAATATGAATTCTTTAAAAACCCAATGTATTTTTTTCCAGAGCATATGGTGTCTTTTGCAACTGAAACCAATGGTGAAATATCCCCCACACAGATTTTGCAGATGTTTCTAAGTTCAAGCACATATCCTGAAATCCATGGCTTCTTACATGCAAAAGAACAGGGAAAGAAGTCCTGGAAAAAGATTTATTTCCTTTTAAGAAGATCCGGTTTATATTTTTCTACTAAAGGGACATCAAAGGAACCACGGCATTTGCAGTTTTTCAGCGAATTTGGCAATAGT[A/G]ATATTTATGTGTCACTGGCAGGCAAAAAAAAACACGGAGCACCGACTAACTATGGATTCTGCTTTAAGCCTAACAAAGCGGGAGGGCCCCGAGACCTGAAAATGCTCTGTGCAGAAGAAGAGCAGAGTAGGACGTGCTGGGTGACCGCGATTAGATTGCTTAAGTATGGCATGCAGCTGTACCAGAATTACATGCATCCATATCAAGGCAGAAGTGGCTACAGTTCTCAGAGTATATCTCCCATGAGAAGTATATCAGAGAATTCCCTGGTAGCAATGGACTTCTCAGGCCAGAAAACAAGAGTTATAGAAAATCCCACTGAAGCCCTTTCTGTTGCAGTTGAAGAAGGACTAGCTTGGAGGAAAAAAGGATGTTTGCGCCTGGGCAGTCACGGTAGCCCCACTGCATCCTCACAGAGCTCTGCCACAAGCATGGCTATCCACCGGTCCCAACCATGGTTTCACCACAAAATTTCTAGAGAGGAAGCTCAGCGATTGATTATTCAGCAAGGTCTAGTGGATGGAGTTTTCTTGGTACGGGATAGTCAGAGTAACCCCAAAACTTTTGTACTGTCAATGAGTCATGGACAAAAAATAAAGCACTTTCAAATTATACCAGTGGAAGATGATGGTGAGATGTTCCACACCCTAGATGATGGACATACAAGATTTAC[A/G]GATCTAATCCAGTTGGTGGAGTTCTATCAACTCAATAAGGGCGTTCTTCCTTGCAAGCTGAAACATTATTGTGCTAGGATTGCACTTTAG

_______________________________________________________________________________________

>COBLL1_XM_012156034_AWASSI

ATGGATCAGAAAGAGAACATGATGGATAAAGACATTGAACTCTTGGTGGTTCTACCTGGAGATATTATCAAGTCTACTACTGTTAATGGCAGTAAACCTATGATGGACTTGTTGATTTTCCTCTGTGCACAATATCACTTAAATCCATCAAGTCACACAATTGATCTGCTCTCAGCTGAAAAGAACCCCATTAAATTTAAGCCAAACACACCAATAGGAATGTTGGAGGTGGAGAAAGTAATTTTAAAGCCAAAAACTCTGGATAAGAAAAAACCTACACCTCTAATACCAGAGAAAACTGTGAGAGTAGTGATCAATTTTAAGAAAACGCAGAAGACAATAGTGAGAGTTAGCCCACACGCACCACTTCAAGAACTTCTCGCCATTATATGCAGCAAATGTGAGTTTGATCCATTACACACACAGTTGCTGAAGGACTATCAGTCTCAGGAGCCCCTTGACTTGACAAAATCTCTTAATGAACTGGGACTGAGAGAATTATATGCCATCGATGTCAGCAAAGCCACTTCTGTTACTGCCTTCAATAAGTCATCTTTACAAGAGTCCTGCCAAATATCACAAAACCTAGACCTTATGAAGGATAAAGAAAGTAAAGGGATTTTCAGCTTATTTCAACGCAGTAAGAAAAAGCGGGAGCAAACTGTCAGCGCCCCTGCGACTCCTTTAGTAAGTAAGCACCGGCCAACTTTTACAAAGAGCAATACCATTTCCAAGCAGTATATTTCAAACACCCTGCCGTCAGATGCTCCCAAGAAGAGGCGTGCTCCACTGCCTCCAATGCCAGGGCCTCAGAGTGCCCCCCAGGACCCTGCACACATTCAGGAGAGGCCAGCTTCTTGTATGGTGAAATCCCCGAGTGTGGATGAAACAGAGGAGAGTTCCTATGGAGCAGGCAGGGTGAGGACGGGTTCTCTGCAGCTCAGTAGCACATCAGCTGGGAATTCATCTTTGAAAAGGACAAAGCGAAAAGCACCTTCCCCGCCCTCGACAACAGTCCTGCTGCAAAGCAATGAGAACAGTCATGTGACTGCCACACAGTCAGTACCTGCAGTTCCTACAGACAGTGGCATAGAAGTGAGCTCTTCTGAGGGCCTGTCCAGCCCAGAAGCCTCCCTTGGCCCTGGGAACGAGCAGTGCGCTGTGTCCCAGCTGCCCGC[T/C]GAAGCCCCTGTCTCGGGGTGCCCTGGAACGCCTGAGGCTGCTGCCACATCTCTGCCCTCTGGAATAAGCTCTGATTATAGCCTTGAAGAGATAGATGAAAAGGAAGAACTGAGTGAAATGCCTAAAGATGAGGCTGAAAATACTTTTCTGAAGTCACAAGACATTCCTACTGTATCTACTGATATAATAAATACACTGAAAAATGATCCTGACTCAGCCCCTGGCAGTGCTACTGGAGAGTCCTCACAAAACTCCAAAGAAGAAAAACAAGGAACTAGAAACACAGATGAACAAAGGCCACACATTGTGGTATATAATTCAAGCGACAAAGAGACGGTAGTTGACAGTGTAAGAAACTTTAAGTCACTGGACCCAAACCAAGAGAAAGCAGATCAACATGAAATAATTGTCATTCCAACAAACAGAGAAAATAATATGAAAAATGGAGTGAGGGGAACAGAAATCAATGTAGCAGGCGTTGCCAAAAATAACAACGTGGACATAGAAGTTGACAGACTATCAAATTATCAGGCATATAAAACTGATACTGCTGGATGTTACAAAGAAAATCTTTCTGTTTCATCAGCACCAGATCAAAACCTGATTCAACCCAGTGCAGAAAAGACAAAAATGCAAGATGCGGCAATTCAGACAATCCCTTCCTGTGACAGTTTTGATGGGGATCAGCAAGATCATAATTTGTCTGACGTCAAAGTTGATGAAAGTGTGCAAACTTCAAGTAACAACAAATCAACTCAACGCTTATCTTTGAGCCCACAAGATTCTGTAGATATCTCAGGAGAATTCAGGAGTCAGGGCCCCCTTGTACATACAGAGGAGCAACTCACCATAAAAGATCCATCTTGTGCAGGTGGTAATGACAATCTTTTGCCTCCTGTAGATGGAACTGATAAAAATTCAACAGCTTCTTATGTAAAGAATTATCCATTTTACAGACAAGATTATATTCCCAAGCCAAAACCTTCAAATGAAATTACAAGAGAGTACATACCCAAAATTGGGATGACTACTTATAAAATAGTGCCTCCCCGATCCCTAGAAATATCCAAAGATTGGGAATCGGAAACCA[C/T]AGGGTATAAAAATGATCAAGAGATACATACTTTAGGGAAAAAGGACACTTATGAGAATGTGAAAGAAACGACAGTCCAAACAGAAGATCTCCTCATTTCTGAAAGCCCAAAGGAGTCACAAGCAGACCTGAAATCAAAGCCTACCCTGAGAACAGAGCAGCAGATGCAAAGTGAGGAGAGCTTAACCCGCAGCAGAATGGTGAATCCTCTGAAACCTCCCAGAATGACGAGTGACACTGGCACGGCTCCTTTTGCGCCAAAATTGGAAGATATAAACAATATTTTGGAGTCAAAATTTAAATCGCGGGTTTCAAATCCCCACTCCAAACCAAGCGCTTTTTTCTTGCAGATGCAAAAAAGAGTTTCAGATCACTATGTGACATCTGCAGCCGCCAAGAGTGTTCAAGCTGCCTCTAATCCTGCTCCAAAGGAATTAATAAAGAAAGAGGTGGAAAGAGATACAATACCTCCTCCAGAGCCAGCTCTTTCTCCCTTAAGTAAAACTATTCAATCTCTTCCACAGACACACATTCAAAACACTGATGATGATAGCAATCAGAAGCCTACTGAAACCTCTCCTCCTGTGGCCTCTCCGCCGCCTCCACCTCCCCCTCCTCCCGTGGCCGCCCCGCTTCTCCCTCCTCCCGTGGCCTCCCCCCTTCCCCCCCCCCCCCTGTTCCTCGGGCATCTACTCTGGGTTCCTCTGCCCAAGAGTCAGTTAGCAATGCTGAATCTGAGAACTCTGAAGACTTTTGGTGCCCCACGACCATACTCCAGCTCTGCTCCTTCACCGTTTGCCCTCGCTGTGGTGAAACGGTCACAGTCTTTCAGCAAAAGCCCTACAGAGTCATGCAGCGAGGAGGTCAAGG[G/T]TGCCTCTGCCAGAACTCCAACTGATGCAGAGAAAGGGAAGATTCCTTC[T/C]GTAAATACATCTGGGAACATGCCACAACTAGGTGTGAGTGATAAGG[T/A]AAATAACTCTGCGCATAATGAACAGAATCCTCAAATACCATCTCCAACTGACTGCCCGTCGG[C/T]CACCCTTAAGAGGCAAAGTTCTGTGACATTCCAAAGCTCTGACCCAGAACAGATCCGACAGAGCTTGCTGACTGCAATTCGTTCTGGAGAGGCTGCTGCCAAACTGAAAAGGGTTACAGTTCAATCAAATACAATATATGTGAATGGAAAGTCAAGACTCAGCCGTTCTGTGTCCCTTGATTCCCCGGGTAACCGTTAA

>COBLL1_XM_012156034_Afec ASSAF

ATGGATCAGAAAGAGAACATGATGGATAAAGACATTGAACTCTTGGTGGTTCTACCTGGAGATATTATCAAGTCTACTACTGTTAATGGCAGTAAACCTATGATGGACTTGTTGATTTTCCTCTGTGCACAATATCACTTAAATCCATCAAGTCACACAATTGATCTGCTCTCAGCTGAAAAGAACCCCATTAAATTTAAGCCAAACACACCAATAGGAATGTTGGAGGTGGAGAAAGTAATTTTAAAGCCAAAAACTCTGGATAAGAAAAAACCTACACCTCTAATACCAGAGAAAACTGTGAGAGTAGTGATCAATTTTAAGAAAACGCAGAAGACAATAGTGAGAGTTAGCCCGCACGCACCACTTCAAGAACTTCTCGCCATTATATGCAGCAAATGTGAGTTTGATCCATTACACACACAGTTGCTGAAGGACTATCAGTCTCAGGAGCCCCTTGACTTGACAAAATCTCTTAATGAACTGGGACTGAGAGAATTATATGCCATCGATGTCAGCAAAGCCACTTCTGTTACTGCCTTCAATAAGTCATCTTTACAAGAGTCCTGCCAAATATCACAAAACCTAGACCTTATGAAGGATAAAGAAAGTAAAGGGATTTTCAGCTTATTTCAACGCAGTAAGAAAAAGCGGGAGCAAACTGTCAGCGCCCCTGCGACTCCTCTAGTAAGTAAGCACCGGCCAACTTTTACAAAGAGCAATACCATTTCCAAGCAGTATATTTCAAACACCCTGCCGTCAGATGCTCCCAAGAAGAGGCGTGCTCCACTGCCTCCAATGCCAGGGCCTCAGAGTGCCCCCCAGGACCCTGCACACATTCAGGAGAGGCCAGCCTCTTGTATGGTGAAATCCCCGAGTGTGGATGAAACAGAGGAGAGTTCCTATGGAGCAGGCAGGGTGAGGACGGGTTCTCTGCAGCTCAGCAGCACATCTGCTGGGAATTCATCTTTGAAAAGGACAAAGCGAAAAGCACCTTCCCCGCCCTCGACAACAGTCCTGCTGCAAAGCAATGAGAACAGTCATGTGACTGCCACACAGTCAGTACCTGCAGTTCCTACAGACAGTGGCATAGAAGTGAGCTCTTCTGAGGGCCTGTCCAGCCCAGAAGCCTCCCTTGGCCCTGGGAACGAGCAGTGCGCTGTGTCCCAGCTGCCCGCTGAAGCCCCTGTCTCGGGGTGCCCTGGAACGCCTGAGGCTGCTGCCACATCTCTGCCCTCTGGAATAAGCTCTGATTATAGCCTTGAAGAGATAGATGAAAAGGAAGAACTGAGTGAAATGCCTAAAGATGAGGCTGAAAATACTTTTCTGAAGTCACAAGACAGTCCTACTGTATCTACTGATATAATAAATACACTGAAAAATGATCCTGACTCAGCCCCTGGCAGTGCTACTGGAGAGTCCTCACAAAACTCCAAAGAAGAAAAACAAGGAACTAGAAACACAGATGAACAAAGGCCACACATTGTGGTATATAATTCAAGCGACAAAGAGACGGTAGTTGACAGTGTAAGAAACTTTAAGTCACTGGACCCAAACCAAGAGAAAGCAGATCAACATGAAATAATTGTCATTCCAACAAACAGAGAAAATAATATGAAAAATGGAGTGAGGGGAACAGAAATCAATGTAGCAGGCGTTGCCAAAAATAACAACGTGGACATAGAAGTTGACAGACTATCAAATTATCAGGCATATAAAACTGATACTGCTGGATGTTACAAAGAAAATCTTTCTGTTTCATCAGCACCAGATCAAAACCTGATTCAACCCAGTGCAGAAAAGACAAAAATGCAAGATGCGGCAATTCAGACAATCCCTTCCTGTGACAGTTTTGATGGGGATCAGCAAGATCATAATTTGTCTGACGTCAAAGTTGATGAAAGTGTGCAAACTTCAAGTAACAACAAATCAACTCAACGCTTATCTTTGAGCCCACAAGATTCTGTAGATATCTCAGGAGAATTCAGGAGTCAGGGCCCCCTTGTACATACAGAGGAGCAACTCAC[T/C]ATAAAAGATCCATCTTGTGCAGGTGGTAATGACAATCTTTTGCCTCCTGTAGATGGAACTGATAAAAATTCAACAGCTTCTTATGTAAAGAATTATCCATTTTACAGACAAGATTATATTCCCAAGCCAAAACCTTCAAATGAAATTACAAGAGAGTACATACCCAAAATTGGGATGACTACTTATAAAATAGTGCCTCCCCGATCCCTAGAAATATCCAAAGATTGGGAATCGGAAACCA[C/T]AGGGTATAAAAATGATCAAGAGATACATACTTTAGGGAAAAAGGACACTTATGAGAATGTGAAAGAAACGACAGTCCAAACAGAAGATCTCCTCATTTCTGAAAGCCCAAAGGAGTCACAAGCAGACCTGAAATCAAAGCCTACCCTGAGAACAGAGCAGCAGATGCAAAGTGAGGAGAGCTTAACCCGCAGCAGAATGGTGAATCCTCTGAAACCTCCCAGAATGACGAGTGACACTGGCACGGCTCCTTTTGCGCCAAAATTGGAAGATATAAACAATATTTTGGAGTCAAAATTTAAATCGCGGGTTTCAAATCCCCACTCCAAACCAAGCGCTTTTTTCTTGCAGATGCAAAAAAGAGTTTCAGATCACTATGTGACATCTGCAGCCGCCAAGAGTGTTCAAGCTGCCTCTAATCCTGCTCCAAAGGAATTAATAAAGAAAGAGGTGGAAAGAGATACAATACCTCCTCCAGAGCCAGCTCTTTCTCCCTTAAGTAAAACTATTCAATCTCTTCCACAGACACACATTCAAAACACTGATGATGATAGCAATCAGAAGCCTACTGAAACCTCTCCTCCTGTGGCCTCTCCGCCGCCTCCACCTCCCCCTCCTCCCGTGACTGCCCCGCTTCCCCCTGCTCCCGTGGCCTCCCCCCTTTCCCCTCCTCCCGTGGCCGCCCCGCTTCCCCCTCCTCCCGTGGCCTCCAAACCCATCTACTCTGGGTTCCTCTGCCCAAGAGTCAGTTAGCAATGCTGAATCTGAGAACTCTGAAGACTTTTGGTGCCCCACGACCATACTCCAGCTCTGCTCCTTCACCGTTTGCCCTCGCTGTGGTGAAACGGTCACAGTCTTTCAGCAAAAGCCCTACAGAGTCATGCAGCGAGGAGGTCAAGG[G/T]TGCCTCTGCCAGAACTCCAACTGATGCAGAGAAAGGGAAGATTCCTTC[C/T]GTAAATACATCTGGGAACATGCCACAACTAGGTGTGAGTGATAAGG[T/A]AAATAACTCTGCGCATAATGAACAGAATCCTCAAATACCATCTCCAACTGACTGCCCGTCGG[C/T]CACCCTTAAGAGGCAAAGTTCTGTGACATTCCAAAGCTCTGACCCAGAACAGATCCGACAGAGCTTGCTGACTGCAATTCGTTCTGGAGAGGCTGCTGCCAAACTGAAAAGGGTTACAGTTCAATCAAATACAATATATGTGAATGGAAAGTCAAGACTCAGCCGTTCTGTGTCCCTTGATTCCCCGGGTAACCGTTAA

_______________________________________________________________________________________
