# Supplementary material for: Unveiling genomic regions that underlie differences between Afec-Assaf sheep and its parental Awassi breed
Source: Genet Sel Evol. 2017 Feb 10;49:19. doi: 10.1186/s12711-017-0296-3 (PMC5301402; doi:10.1186/s12711-017-0296-3)
Supplement: Supplementary file 8 — Additional file 8: Figure S2. Variability in fat-tail phenotypes in Assaf sheep. Awassi-like fat tail (A). Fat tails with different amounts of fat deposition and different lengths (B to H). [file 12711_2017_296_MOESM8_ESM.docx]

**Figure S2.** Variability in fat-tail phenotypes in Assaf sheep

| 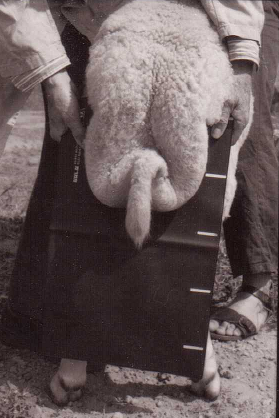 | 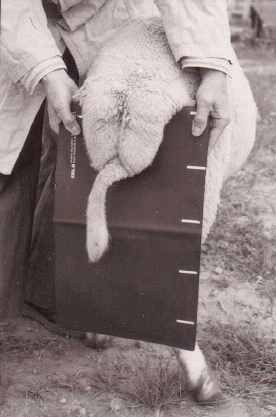 | 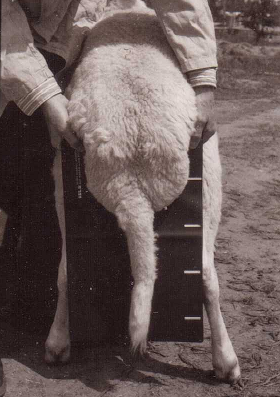 | 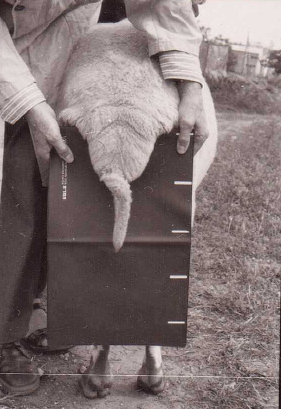 |
| --- | --- | --- | --- |
| A | B | C | D |
| 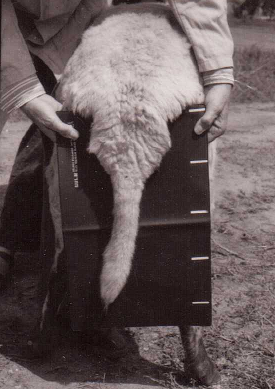 | 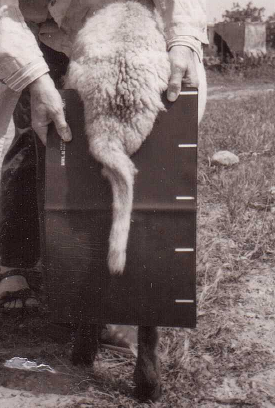 | 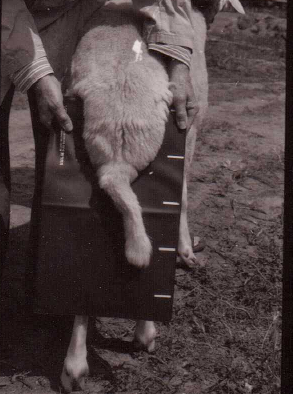 | 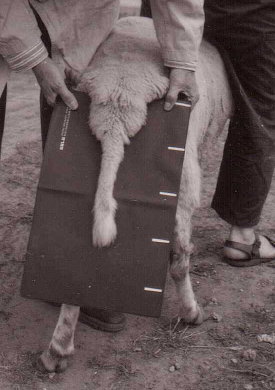 |
| E | F | G | H |

(A) Awassi-like fat tail. (B–H) Fat tails with different amounts of fat deposition and different lengths.
