# Supplementary material for: Unveiling genomic regions that underlie differences between Afec-Assaf sheep and its parental Awassi breed
Source: Genet Sel Evol. 2017 Feb 10;49:19. doi: 10.1186/s12711-017-0296-3 (PMC5301402; doi:10.1186/s12711-017-0296-3)
Supplement: Supplementary file 9 — Additional file 9: Table S6. Candidate genes and SNP markers associated with carrying fat tail in sheep [38, 39, 47, 49–51]. [file 12711_2017_296_MOESM9_ESM.docx]

**Table S6.** Candidate genes and SNP markers associated with carrying fat tail in sheep

| Ref | Breed comparison | | Candidate gene/SNP | Position on sheep genomev3.1 according to UCSC Genome browser | GWAS (#)* |
| --- | --- | --- | --- | --- | --- |
|  | Fat-tailed/fat-rumped | Thin-tailed |  |  |  |
| [50] | Lori-Bakhtiari, Chall | Zel, Zel-Atabay cross | *CAST* | OAR5: 93,354,399 - 93,484,087 |  |
| [51] | Lori-Bakhtiari | Zel | *FABP4* | OAR9: 57,356,525 - 57, 541,042 |  |
| [47] | Lori-Bakhtiari | Zel | s20468.1  s58048.1 | OAR2: 52,409,379  OAR2: 52,441,926 |  |
|  |  |  | s55322.1  OAR5_47175489.1 OAR5_47263230.1 | OAR5: 43,128,620  OAR5: 43,154,107  OAR5: 43,236,671 |  |
|  |  |  | OAR7_46642359.1 OAR7_46765080.1 OAR7_46818598.1 OAR7_46843356.1 | OAR7: 42,042,691  OAR7: 42,133,836  OAR7: 42,202,240  OAR7: 42,227,199 |  |
|  |  |  | OARX_59194976.1 OARX_59257971.1 OARX_59327581.1 OARX_59383635.1 OARX_59571364.1 OARX_59578440.1 OARX_59912586.1  s38079 | OARX: 55,788,390  OARX: 55,840,148  OARX: 55,909,305  OARX: 55,937,193  OARX: 56,153,635  OARX: 56,165,053  OARX: 56,538,556  OARX: 56,741,416 |  |
| [49] | Kazakh sheep | Tibetan sheep | *LOC101105114* | OAR1: 211,19,116–211,39,870 |  |
|  |  |  | *CBS* | [OAR1: 261,](http://genome-euro.ucsc.edu/cgi-bin/hgTracks?position=chr1:261295143-261315296&hgsid=208579372_ucqY3cZEOm8y9YFcRyhoDUkDjrgQ&ensGene=pack&hgFind.matches=ENSOART00000012104,)297,346-261,311,375 |  |
|  |  |  | *FAM206A* | OAR2: 14,285,230-14,291,163 |  |
|  |  |  | *COL15A1* | [OAR2: 48,487,007- 48,588,324](http://genome-euro.ucsc.edu/cgi-bin/hgTracks?position=chr2:48487007-48588324&hgsid=208579372_ucqY3cZEOm8y9YFcRyhoDUkDjrgQ&ensGene=pack&hgFind.matches=ENSOART00000011990,) |  |
|  |  |  | *CTSB* | [OAR2: 104,431,014-104,438,581](http://genome-euro.ucsc.edu/cgi-bin/hgTracks?position=chr2:104431014-104438581&hgsid=208579372_ucqY3cZEOm8y9YFcRyhoDUkDjrgQ&ensGene=pack&hgFind.matches=ENSOART00000016613,) |  |
|  |  |  | *ASNSD1* | OAR2: 118,521,438-118,532,607 |  |
|  |  |  | *EMG1* | OAR3: 207,439,400-207,444,991 |  |
|  |  |  | *LOC101102230* | OAR4: 40,240,378- 40,311,308 |  |
|  |  |  | *LOC101109877* | OAR5: 92,467,076-92,469,946 |  |
|  |  |  | *LASP1* | [OAR11: 39,031,450-39,073,073](http://genome-euro.ucsc.edu/cgi-bin/hgTracks?position=chr11:39031450-39073073&hgsid=208579372_ucqY3cZEOm8y9YFcRyhoDUkDjrgQ&ensGene=pack&hgFind.matches=ENSOART00000010955,) |  |
|  |  |  | *WIPI1* | OAR11: 60,653,024- 60,677,230 |  |
|  |  |  | *FMO3* | OAR12: 36,801,185-36,828,327 |  |
|  |  |  | *TMEM63A* | OAR12: 27,037,533-27,070,628 |  |
|  |  |  | *PRNP* | [OAR13: 46,](http://genome-euro.ucsc.edu/cgi-bin/hgTracks?position=chr13:46208529-46229267&hgsid=208579372_ucqY3cZEOm8y9YFcRyhoDUkDjrgQ&refGene=pack&hgFind.matches=NM_001009481,)225,267-46,226,024 |  |
|  |  |  | *PPP1CC* | [OAR17:](http://genome-euro.ucsc.edu/cgi-bin/hgTracks?position=chr13:49020246-49022223&hgsid=208579372_ucqY3cZEOm8y9YFcRyhoDUkDjrgQ&ensGene=pack&hgFind.matches=ENSOART00000020421,)  54,151,722-54,157,978 |  |
|  |  |  | *LOC101114311* | OAR18: 57,479,908-57,517,831 |  |
|  |  |  | *LYRM4* | OAR20: 48,394,335- 48,394,478 |  |
|  |  |  | *NELL1* | OAR21: 22,606,980-22676392 |  |
|  |  |  | *ZBED1* | OARX: 11,110,089-11,137,755 |  |
|  |  |  | *BEX5* | OARX: 126,243,407- 126,243,745 |  |
| [38] | Duolang, Kazakh,Lop, Ujimqin, Hu, Tong, Large-tailed Han | Plateau-type Tibetan, Valley-type Tibetan, Diqing | *PDGFD* | OAR15: 3,848,546-4,133,998 |  |
|  |  |  | *PPP1CC* | [OAR17:](http://genome-euro.ucsc.edu/cgi-bin/hgTracks?position=chr13:49020246-49022223&hgsid=208579372_ucqY3cZEOm8y9YFcRyhoDUkDjrgQ&ensGene=pack&hgFind.matches=ENSOART00000020421,) 54,151,722-54,157,978 |  |
| [39] | Laticauda, Cyprus fat-tail sheep | Alpagota, Altamurana, Appenninica, Bergamasca, Biellese, Delle, Langhe, Fabrianese, Gentile, Istrian Pramenka, Massese, Sambucana, Sarda, Sopravissana | *OAR2_115949202*; *OAR2_116017982*; *OAR2_116054738* | OAR2: 107,805,210; 107,866,879; 107,911,874 |  |
|  |  |  | *SLIT2* | OAR6: 39,939,556– 40,091,549 |  |
|  |  |  | VRTN | OAR7: 82,585,586 – 82,587,635 |  |
|  |  |  | ALOX5AP | OAR10: 30,365, 435 – 30,388,526 | **14** |
|  |  |  | FGF9 | OAR10: 35,583, 842 – 35,609,414 | **15** |
|  |  |  | BMP2 | OAR13: 48,462,232 – 48,472,599 |  |

*Affiliation to a genomic region in the Awassi-Afec-Assaf GWAS
