# Supplementary material for: Unveiling genomic regions that underlie differences between Afec-Assaf sheep and its parental Awassi breed
Source: Genet Sel Evol. 2017 Feb 10;49:19. doi: 10.1186/s12711-017-0296-3 (PMC5301402; doi:10.1186/s12711-017-0296-3)
Supplement: Supplementary file 10 — Additional file 10: Table S7. Genes known to affect coat pigmentation in humans, cattle and other mammals [55–58]. [file 12711_2017_296_MOESM10_ESM.docx]

**Table S7.** Genes known to affect coat pigmentation in humans, cattle and other mammals

| Gene symbol | OAR | Affiliation to a genomic region in the Awassi-Afec-Assaf GWAS (#) | Position according to Oar_v3.1 assembly |
| --- | --- | --- | --- |
|  |  |  |  |
| *DOCK7* | 1 |  |  |
| *FOXD3* | 1 |  |  |
| *GPR161* | 1 |  |  |
| *HPS3* | 1 |  |  |
| *HS2ST1* | 1 |  |  |
| *LMX1A* | 1 |  |  |
| *MCOLN3* | 1 |  |  |
| *MLPH* | 1 |  |  |
| *NOTCH2* | 1 |  |  |
| *NTRK1* | 1 | 3 | 105,382,487-105,400,976 |
| *RBP1* | 1 |  |  |
| *RPL24* | 1 | 4 | 164,474,247-164,479,472 |
| *SEMA4A* | 1 | 3 | 104,748,776-104,766,729 |
| *SOX2* | 1 |  |  |
| *TBX15* | 1 |  |  |
| *ECE1* | 2 |  |  |
| *EN1* | 2 |  |  |
| *GAS1* | 2 |  |  |
| *GNAQ* | 2 |  |  |
| *HERC2* | 2 |  |  |
| *IHH* | 2 |  |  |
| *MREG* | 2 |  |  |
| *OCA2* | 2 |  |  |
| *PAX3* | 2 |  |  |
| *PHACTR4* | 2 |  |  |
| *SLC31A1* | 2 |  |  |
| *VLDLR* | 2 |  |  |
| *SLC16A7* | 3 |  |  |
| *ADAM17* | 3 |  |  |
| *ADAMTS20* | 3 |  |  |
| *EDAR* | 3 |  |  |
| *KRT1* | 3 | 8 | 133,566,405-133,604,565 |
| *KRT4* | 3 | 8 | 133,453,262-133,459,729 |
| *KRT75* | 3 | 8 | 133,772,163-133,781,755 |
| *MCHR1* | 3 |  |  |
| *NOTCH1* | 3 |  |  |
| *PAH* | 3 |  |  |
| *PDGFB* | 3 |  |  |
| *PMCH* | 3 |  |  |
| *PMEL (SILV)* | 3 |  |  |
| *POMC* | 3 |  |  |
| *RXRA* | 3 |  |  |
| *TIMP3* | 3 |  |  |
| *WNT1* | 3 |  |  |
| *GLI3* | 4 |  |  |
| *GPNMB* | 4 |  |  |
| *SEMA3C* | 4 |  |  |
| *AP3D1* | 5 |  |  |
| *ATOX1* | 5 |  |  |
| *FKBP8* | 5 |  |  |
| *GNA11* | 5 |  |  |
| *S1PR2* | 5 |  |  |
| *WNT3A* | 5 |  |  |
| *BMPR1B* | 6 | 10 | 29,361,947-29,448,079 |
| *CNO* | 6 |  |  |
| *FGF5* | 6 |  |  |
| *LEF1* | 6 |  |  |
| *PDGFRA* | 6 |  |  |
| *AP3B1* | 7 |  |  |
| *APC* | 7 |  |  |
| *MAP2K1* | 7 |  |  |
| *MYO5A* | 7 |  |  |
| *OTX2* | 7 |  |  |
| *PLDN* | 7 |  |  |
| *PYGO1* | 7 |  |  |
| *RAB27A* | 7 |  |  |
| *RABGGTA* | 7 |  |  |
| *SFXN1* | 7 |  |  |
| *SLC24A5* | 7 |  |  |
| *TRPM7* | 7 |  |  |
| *VSX2* | 7 |  |  |
| *ELOVL4* | 8 |  |  |
| *FIG4* | 8 |  |  |
| *NR2E1* | 8 |  |  |
| *OSTM1* | 8 |  |  |
| *MYC* | 9 |  |  |
| *RECQL4* | 9 |  |  |
| *RPS20* | 9 |  |  |
| *SNAI2* | 9 |  |  |
| *ATP7B* | 10 |  |  |
| *EDNRB* | 10 |  |  |
| *FREM2* | 10 |  |  |
| *RB1* | 10 |  |  |
| *ZIC2* | 10 |  |  |
| *BRCA1* | 11 |  |  |
| *DPH1* | 11 |  |  |
| *FOXN1* | 11 |  |  |
| *JMJD6* | 11 |  |  |
| *KRT17* | 11 |  |  |
| *MED1* | 11 |  |  |
| *NF1* | 11 |  |  |
| *UNC119* | 11 |  |  |
| *ZBTB17* | 12 |  |  |
| *ATRN* | 13 |  |  |
| *GATA3* | 13 |  |  |
| *ITGB1* | 13 |  |  |
| *SOX18* | 13 |  |  |
| *ACD* | 14 |  |  |
| *BLOC1S3* | 14 |  |  |
| *ERCC2* | 14 |  |  |
| *MBTPS1* | 14 |  |  |
| *RPS19* | 14 |  |  |
| *TRAPPC6A* | 14 |  |  |
| *ZFP53* | 14 |  |  |
| *ARCN1* | 15 |  |  |
| *DRD2* | 15 |  |  |
| *MPZL3* | 15 |  |  |
| *MYO7A* | 15 |  |  |
| *PAX6* | 15 |  |  |
| *PTS* | 15 |  |  |
| *TRAF6* | 15 |  |  |
| *TUB* | 15 |  |  |
| *SLC45A2* | 16 |  |  |
| *GGT1* | 17 |  |  |
| *HPS4* | 17 |  |  |
| *MAB21L2* | 17 | 17 | 7,074,126-7,075,193 |
| *PDGFC* | 17 |  |  |
| *SLC7A11* | 17 |  |  |
| *SMARCA5* | 17 |  |  |
| *VPS33A* | 17 |  |  |
| *POLG* | 18 |  |  |
| *SLC24A4* | 18 |  |  |
| *TP53Bp1* | 18 |  |  |
| *EGFR* | 19 |  |  |
| *DST* | 20 |  |  |
| *DTNBP1* | 20 |  |  |
| *IRF4* | 20 |  |  |
| *POLH* | 20 |  |  |
| *TFAP2A* | 20 |  |  |
| *EED* | 21 |  |  |
| *FZD4* | 21 |  |  |
| *HPS5* | 21 |  |  |
| *RAB38* | 21 |  |  |
| *TBX10* | 21 |  |  |
| *TPCN2* | 21 |  |  |
| *ELOVL3* | 22 |  |  |
| *FAS* | 22 |  |  |
| *FGFR2* | 22 |  |  |
| *HELLS* | 22 |  |  |
| *HPS1* | 22 |  |  |
| *HPS6* | 22 |  |  |
| *OAT* | 22 |  |  |
| *PAX2* | 22 |  |  |
| *PITX3* | 22 |  |  |
| *SUFU* | 22 |  |  |
| *BCL2* | 23 |  |  |
| *SMCHD1* | 23 |  |  |
| *MGRN1* | 24 |  |  |
| *PDPK1* | 24 |  |  |
| *BMPR1A* | 25 |  |  |
| *EDARADD* | 25 | 19 | 9,116,404-9,182,219 |
| *GNPAT* | 25 |  |  |
| *LYST* | 25 |  |  |
| *PCBD1* | 25 |  |  |
| *CASP3* | 26 |  |  |
| *DEFB103 (DEFB300 or hBD3)* | 26 |  |  |
| *ATP7A* | x |  |  |
| *CITED1* | x |  |  |
| *GPC3* | x |  |  |
| *GPR143* | x |  |  |
| *HEPH* | x |  |  |
| *L1CAM* | x |  |  |
| *NDP* | x |  |  |
| *RS1* | x |  |  |
| *SHROOM2* | x |  |  |

According to refs. [55-58]. The positions of the genes annotated to the variable genomic regions (VGRs) between Awassi and Afec-Assaf are indicated.
