# Supplementary material for: Unveiling genomic regions that underlie differences between Afec-Assaf sheep and its parental Awassi breed
Source: Genet Sel Evol. 2017 Feb 10;49:19. doi: 10.1186/s12711-017-0296-3 (PMC5301402; doi:10.1186/s12711-017-0296-3)
Supplement: Supplementary file 11 — Additional file 11: Table S8. Human genes associated with fat distribution [61, 63, 64, 98–100]. [file 12711_2017_296_MOESM11_ESM.docx]

**Table S8.** Human genes associated with fat distribution

| Gene | OAR | Position Ovine v 3. 1 | Awassi –Afec Assaf GWAS | | Reference |
| --- | --- | --- | --- | --- | --- |
|  |  |  | Region (#) | Position (Mb) |  |
| *TBX15-WARS2* | 1 | 95,560,945 - 95,683,845 |  |  | [61, 98, 99, 63] |
| *WARS2* | 1 | 95,721,052 - 95,816,528 |  |  | [99] |
| *DCST2* | 1 | 103,804,834 - 103,823,273 | 3 | 103.40-103.45 | [98] |
| *LMNA* | 1 | 104,711,158 - 104,729,637 |  |  | [99] |
| *LPP* | 1 | 196,684,028 - 197,238,618 |  |  | [99] |
| *SHOX2* | 1 | 227,507,349 - 227,515,800 |  |  | [99] |
| *LEKR1* | 1 | 228,810,846 - 228,886,530 |  |  | [98] |
| *TMEM18* | 2 | 481,423 - 485,096 |  |  | [64] |
| *LHX2* | 2 | 11,688,347 - 11,707,288 |  |  | [99] |
| *ABCA1* | 2 | 18,039,778 - 18,168,466 |  |  | [98] |
| *NKX2-6* | 2 | 41,867,521 - 41,871,815 |  |  | [98] |
| *MSRA* | 2 | 102,876,397 - 103,277,001 |  |  | [61, 99] |
| *CALCRL* | 2 | 120,920,134 - 120,966,449 |  |  | [98] |
| *COBLL1* | 2 | 143,684,185 - 143,752,953 | 7 | 145.65-148.41 | [98, 99, 64] |
| *GRB14* | 2 | 143,839,555 - 143,965,488 | 7 | 145.65-148.41 | [61, 98, 63, 64] |
| *EN1* | 2 | 183,092,365 - 183,097,217 |  |  | [99] |
| *BMPR2* | 2 | 203,565,593 - 203,710,593 |  |  | [99] |
| *IRS1* | 2 | 228,093,401 - 228,097,629 |  |  | [61, 64] |
| *AGPAT2* | 3 | 2,728,311 - 2,731,922 |  |  | [99] |
| *TRIB2* | 3 | 21,432,304 - 21,459,979 |  |  | [99] |
| *APOB* | 3 | 28,671,825 - 28,710,732 |  |  | [99] |
| *MEIS1* | 3 | 41,648,874 - 41,799,894 |  |  | [98] |
| *THNSL2-FABP1* | 3 | 58,685,544 - 58,691,134 |  |  | [61] |
| *HOXC8* | 3 | 132,357,083 - 132,359,341 |  |  | [99] |
| *HOXC9* | 3 | 132,365,704 - 132,368,208 |  |  | [99] |
| *HOXC13* | 3 | 132,422,835 - 132,429,375 |  |  | [61, 98, 63] |
| *HMGA2* | 3 | 153,705,703 - 153,846,992 |  |  | [99] |
| *ITPR2-SSPN* | 3 | 188,482,290 - 188,489,681 |  |  | [61, 98, 99, 63] |
| *PLA2G6* | 3 | 213,879,369 - 213,939,800 |  |  | [64] |
| *TWIST1* | 4 | 27,866,988 - 27,868,276 |  |  | [99] |
| *IL6* | 4 | 31,412,389 - 31,416,357 |  |  | [99] |
| *CAV1* | 4 | 51,785,966 - 51,820,791 |  |  | [99] |
| *HOXA11* | 4 | 68,846,949 - 68,847,134 |  |  | [98] |
| *HOXA10* | 4 | 68,858,042 - 68,863,494 |  |  | [99] |
| *HOXA5* | 4 | 68,876,468 - 68,891,655 |  |  | [99] |
| *HOXA4* | 4 | 68,903,112 - 68,904,155 |  |  | [99] |
| *NFE2L3* | 4 | 69,731,701 - 69,740,892 |  |  | [61, 98, 63] |
| *LEP* | 4 | 92,508,289 - 92,522,182 |  |  | [99] |
| *CRTC1* | 5 | 4,205,347 - 4,325,277 |  |  | [64] |
| *JUND* | 5 | 4,674,908 - 4,675,163 |  |  | [98] |
| *APM1* | 5 | 6,541,476 - 6,559,356 |  |  | [99] |
| *FGFR4* | 5 | 36,052,758 - 36,060,143 |  |  | [98] |
| *GR-NR3C1* | 5 | 51,733,916 - 51,850,972 |  |  | [99] |
| *HSD17B4* | 5 | 31,819,802 - 31,921,872 |  |  | [99] |
| *NR2F1* | 5 | 90,397,738 - 90,406,529 |  |  | [99] |
| *TNFAIP8-HSD17B4* | 5 | 31,819,802 - 31,921,874 |  |  | [98] |
| *FAM13A* | 6 | 35,985,266 - 35,985,381 |  |  | [98] |
| *NMU* | 6 | 71,047,675 - 71,065,746 |  |  | [98] |
| *SMAD6* | 7 | 13,318,677 - 13,395,361 |  |  | [98] |
| *RFX7* | 7 | 51,216,615 - 51,356,350 |  |  | [98] |
| *NRXN3* | 7 | 86,760,256 - 87,154,967 |  |  | [61, 99] |
| *RSPO3* | 8 | 11,210,668 - 11,265,163 |  |  | [61, 98, 63] |
| *CNR1-CB1R* | 8 | 48,928,604 - 48,930,022 |  |  | [99] |
| *MSC* | 9 | 48,249,988 - 48,251,474 |  |  | [98] |
| *LHFP* | 10 | 23,066,861 - 23,125,253 |  |  | [99] |
| *SPRY2* | 10 | 55,821,641 - 55,822,306 |  |  | [64] |
| *PEMT* | 11 | 34,333,041 - 34,356,134 |  |  | [98] |
| *IGF2BP1* | 11 | 37,058,898 - 37,099,743 |  |  | [64] |
| *PTRF* | 11 | 41,994,203 - 42,005,842 |  |  | [99] |
| *FASN* | 11 | 49,940,273 - 49,955,854 |  |  | [99] |
| *KCNJ2* | 11 | 59,478,483 - 59,479,865 |  |  | [98] |
| *LYPLAL1-SLC30a10* | 12 | 20,785,849 - 20,814,579 |  |  | [61, 98, 63] |
| *GORAB* | 12 | 36,253,410 - 36,276,172 |  |  | [98] |
| *DNM3-PIGC* | 12 | 38,066,986 - 38,067,879 |  |  | [61, 98, 99, 63] |
| *SEC16B* | 12 | 57,403,574 - 57,448,222 |  |  | [64] |
| *BMP2* | 13 | 48,462,232 - 48,472,599 |  |  | [98] |
| *GDF5* | 13 | 64,242,058 - 64,245,799 |  |  | [98] |
| *SNX10* | 13 | 73,107,307 - 73,108,440 |  |  | 98] |
| *EYA2* | 13 | 75,066,765 - 75,328,455 |  |  | [98] |
| *CMIP* | 14 | 7,590,795 - 7,670,033 |  |  | [98] |
| *FTO* | 14 | 21,524,991 - 21,953,995 |  |  | [64] |
| *CEBPA* | 14 | 43,019,889 - 43,119,889 |  |  | [98] |
| *TOMM40* | 14 | 51,689,549 - 51,700,871 |  |  | [64] |
| *CPEB4* | 16 | 5,642,018 - 5,715,828 |  |  | [61, 98, 63] |
| *MAP3K1* | 16 | 22,393,215 - 22,463,925 |  |  | [98] |
| *SFRP2* | 17 | 3,719,522 - 3,727,430 |  |  | [99] |
| *SPATA5-FGF2* | 17 | 34,517,874 - 34,572,443 |  |  | [98] |
| *CCDC92* | 17 | 51,371,109 - 51,375,133 |  |  | [98] |
| *HECTD4 (C12orf51)* | 17 | 61,505,822 - 61,672,365 |  |  | [61] |
| *ZNRF3* | 17 | 67,924,280 - 67,930,559 |  |  | [61, 98, 99, 63] |
| *KLF13* | 18 | 26,515,214 - 26,702,939 |  |  | [98] |
| *ADAMTS9* | 19 | 36,783,834 - 36,945,351 |  |  | [61, 63] |
| *PBRM1* | 19 | 48,153,833 - 48,251,508 |  |  | [98] |
| *STAB1* | 19 | 48,314,839 - 48,339,163 |  |  | [99] |
| *NISCH-STAB1* | 19 | 48,342,058 - 48,400,530 |  |  | [61, 99, 63] |
| *PLXND1* | 19 | 55,994,633 -56,043,073 |  |  | [98] |
| *PPARG* | 19 | 56,552,358 - 56,652,679 |  |  | [99, 100] |
| *HMGA1* | 20 | 8,438,950 - 8,441,572 |  |  | [98] |
| *VEGFA* | 20 | 17,368,866 - 17,382,112 |  |  | [61, 98, 63] |
| *TFAP2B* | 20 | 23,153,576 - 23,178,184 |  |  | [61, 99] |
| *BTNL2* | 20 | 25,793,691 - 25,811,932 |  |  | [98, 63] |
| *RREB1* | 20 | 47,013,295 - 47,071,943 |  |  | [99] |
| *LY86* | 20 | 47,482,044 - 47,522,944 |  |  | [61, 98, 63] |
| *BSCL2* | 21 | 40,407,770 - 40,419,617 |  |  | [99] |
| *MACROD1-VEGFB* | 21 | 41,846,214 - 41,850,496 |  |  | [98] |
| *IGF2-H19* | 21 | 48,655,290 - 48,680,999 |  |  | [99] |
| *RBP4* | 22 | 14,598,701 - 14,604,427 |  |  | [99] |
| *SFXN2* | 22 | 22,476,849 - 22,485,542 |  |  | [98] |
| *MC4R* | 23 | 59,385,576 - 59,386,574 |  |  | [61, 99] |
| *BCL2* | 23 | 61,820,658 - 61,821,263 |  |  | [98] |
| *TUFM* | 24 | 25,971,607 - 25,977,898 |  |  | [64] |
| *PAI-1* | 24 | 35,490,295 - 35,496,243 |  |  | [99] |
| *SIRT1* | 25 | 24,228,851 - 24,253,902 |  |  | [99] |
| *BMPR1A* | 25 | 41,109,681 - 41,138,116 |  |  | [99] |
| *AGT* | 25 | 45,303,043 - 45,309,821 |  |  | [99] |
| *ADRB3* | 26 | 31,849,634 - 31,851,548 |  |  | [99] |
| *GPC4* | X | 96,383,593 - 96,417,364 |  |  | [99] |
